# Supplementary material for: Stereoretentive Post-Translational Protein Editing
Source: ACS Cent Sci. 2023 Feb 24;9(3):405–16. doi: 10.1021/acscentsci.2c00991 (PMC10037454; doi:10.1021/acscentsci.2c00991)
Supplement: Supplementary file 1 — oc2c00991_si_001.pdf [file oc2c00991_si_001.pdf]

SUPPLEMENTARY INFORMATION

Stereoretentive Post-Translational Protein Editing

Xia-Ping Fu[1,2]¥, Yizhi Yuan[1,2]¥, Ajay Jha[1,2], Nikita Levin[1], Andrew M. Giltrap[1,2], Jack Ren[1,3], Dimitrios Mamalis[1,3], Shabaz Mohammed[1,3], Benjamin G. Davis*[1,2,3]

[1] Rosalind Franklin Institute, Harwell, Oxfordshire, OX11 0QX, UK

[2] Department of Pharmacology, University of Oxford, Oxford, OX1 3QT UK

[3] Department of Chemistry, University of Oxford, Oxford, OX1 3TA, UK

¥ These authors contributed equally.

*Email: Ben.Davis@rfi.ac.uk

Supplementary Figures S1-S18, Supplementary Tables S1-S2, Supplementary Methods and Supplementary References.

Supplementary Figures 4
Supplementary Figure S1: MS/MS analysis of PstS-Ala178. 5
Supplementary Figure S2: MS/MS analysis of PstS-Bal178. 6
Supplementary Figure S3: MS/MS analysis of PstS-TEMPO-A178 7
Supplementary Figure S4: MS/MS analysis of PstS-SecPh178. 8
Supplementary Figure S5: MS/MS analysis of PstS-Sel178 9
Supplementary Figure S6: MS/MS analysis of PstS-Mal-L178. 10
Supplementary Figure S7: MS/MS analysis of PstS-Hag178 11
Supplementary Figure S8: MS/MS analysis of PstS-A(Ketone)178 12
Supplementary Figure S9: MS/MS analysis of PstS-Lys178 13
Supplementary Figure S10: MS/MS analysis of PstS-KAc178 14
Supplementary Figure S11: MS/MS analysis of Psts-Ser178 15
Supplementary Figure S12: Spectral signature of EDA complexation 16
Supplementary Figure S13: Spectral signature of EDA complexation on protein 17
Supplementary Figure S14: Frontier molecular orbitals from DFT Calculations 18
Supplementary Figure S15: MS/MS analysis of Psts-A(Sulphone)178 19
Supplementary Figure S16: Testing of Disulfide Compatibility in the Editing of Single Domain Ab cabVCAM to form cabVCAM-Bal118 and the Disruptive Effect of TCEP. ...20
Supplementary Figure S17: Testing of Marfey's Analysis for Detection of Configuration in edited TEV-HistoneH3-Ser2 protein. 23
Supplementary Figure S18: Possible Modes of C–S Homolysis to Putative On-protein C• Side-chain Radical Generation for Stereoretentive Sidechain Editing. 25
Supplementary Tables 27
Supplementary Table S1: Optimisation of chemical introduction of tetrafluoropyridyl-cysteine (Fpc) into proteins 28
Supplementary Table S2: Optimisation of light-mediated C_β-S_γ bond cleavage 29
Supplementary Methods 31
General Experimental Procedures 31
Protein Mass Spectrometry 31
Protein digestion and analysis by MS/MS 32
General procedure 1: Cys-tetrafluoropyridylsulfide (Fpc) formation in proteins 34
General procedure 2: Alanyl radical trapped by HAT 34
General procedure 3: Alanyl radical trapped by TEMPO 34
General procedure 4: Alanyl radical trapped by diselenides 34
General procedure 5: Alanyl radical trapped by alkenes 34
General procedure 6: Alanyl radical trapped by B_2Cat_2. 35
General procedure 7: Alanyl radical formally trapped by O_2. 35
General procedure 8: Chemical introduction of boronoalanine (Bal) into proteins. 35

Histone H3-Fpc2, -Fpc4, -Fpc9, -Fpc18, -Fpc27, Histone H4-Fpc20: protein expression, purification and Fpc generation....36
PstS -Fpc57, -Fpc103, -Fpc178, -Fpc197: protein expression, purification and Fpc generation....43
pre-SUMO1-Fpc51: protein expression, purification and Fpc generation....48
Npβ-Fpc61: protein expression, purification and Fpc generation....50
cAbVCAM1-Fpc118: protein expression, purification and Fpc generation....52
AcrA-Fpc123: protein expression, purification and Fpc generation....54
On-protein radical trapping from PstS-Fpc178....56
Widescale introduction of L-boronoalanine (L-Bal) into Proteins. 71
Characterization of the retention of native L-stereochemistry by protein ^{19}F NMR. 84
Creation of H3-L-KAc18 and Assessment of Enzymatic Processing as a Substrate using Sirt 2....85
Marfey's Analysis of TEV-HistoneH3-Ser2....85
DFT Calculations for Model PyF Containing Systems....87
Supplementary References....91

Supplementary Figures

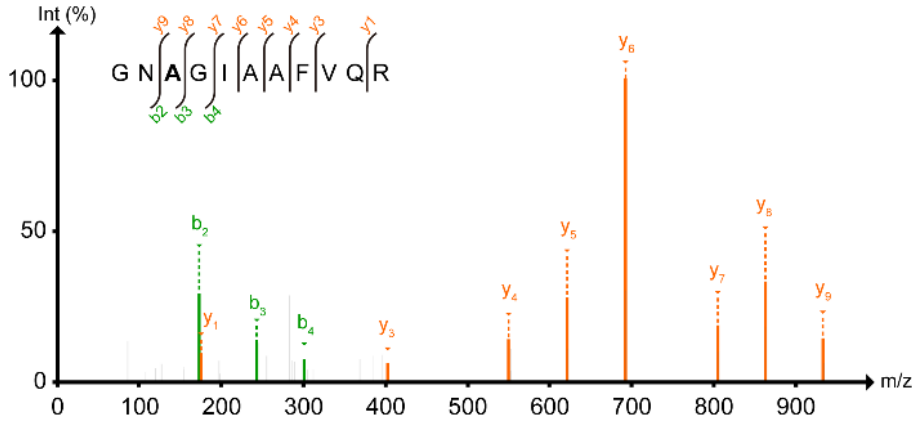

Supplementary Figure S1: MS/MS analysis of PstS-Ala178.

HCD (30% normalised collision energy) mass spectrum of $[GNAGIAAFVQR]^{2+}$, acquired in the tandem LC-MS analysis of the tryptic digest of PstS-Ala178. The peak at m/z = 932.5 Th corresponds to the mass of the fragment y_9.

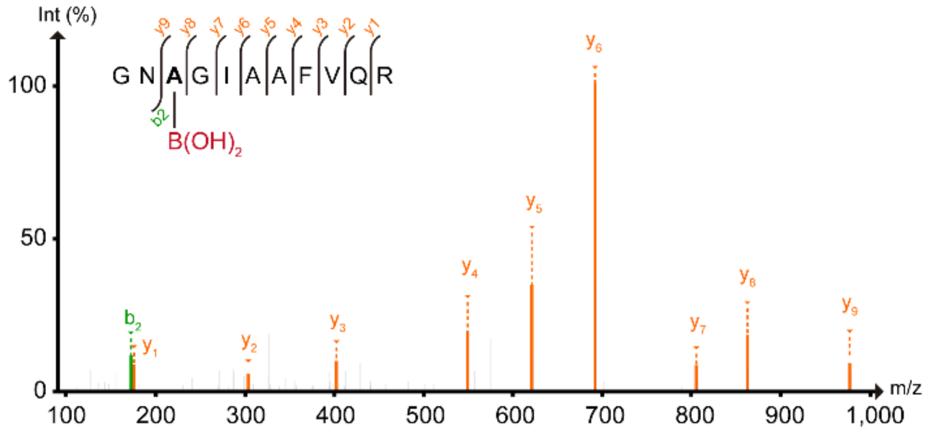

Supplementary Figure S2: MS/MS analysis of PstS-Bal178.

HCD (30% normalised collision energy) mass spectrum of [GNA(B(OH)2GIAAFVQR]$^{2+}$, acquired in the tandem LC-MS analysis of the tryptic digest of PstS-Bal178. The peak at m/z = 976.5 Th corresponds to the mass of the fragment y_9 modified by $B(OH)_2$.

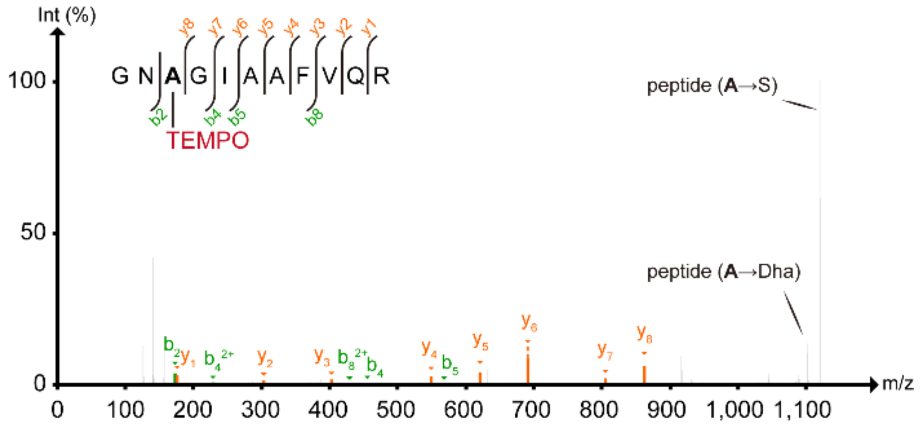

Supplementary Figure S3: MS/MS analysis of PstS-TEMPO-A178

HCD (30% normalised collision energy) mass spectrum of [GNA(TEMPO)GIAAFVQR]$^{2+}$, acquired in the tandem LC-MS analysis of the tryptic digest of PstS-TEMPO-A178. The peak at m/z = 1101.6 Th corresponds to the mass of [GN(Dha)GIAAFVQR]$^{+}$ resulting from the collisional dissociation of the C-O bond between Ala178 residue and TEMPO modification, and the peak at m/z = 1119.6 Th corresponds to the mass of [GNSGIAAFVQR]$^{+}$ resulting from the collisional dissociation of O-N bond in the TEMPO modification.

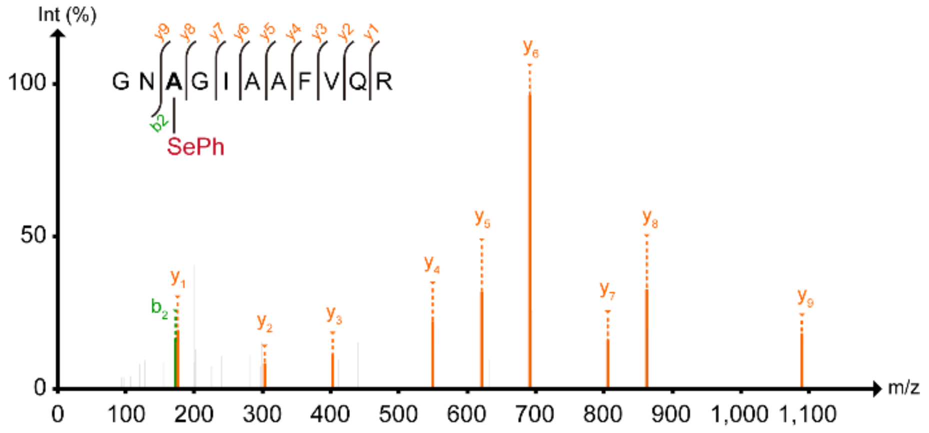

Supplementary Figure S4: MS/MS analysis of PstS-SecPh178.

HCD (30% normalised collision energy) mass spectrum of [GNA(SePh)GIAAFVQR]$^{2+}$, acquired in the tandem LC-MS analysis of the tryptic digest of PstS-SecPh178. The peak at m/z = 1088.5 Th corresponds to the mass of the fragment y_9 modified by SePh.

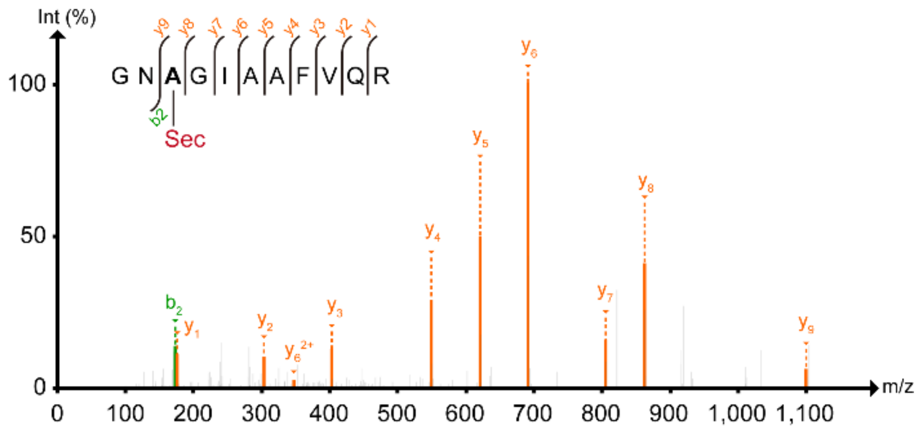

Supplementary Figure S5: MS/MS analysis of PstS-Sel178

HCD (30% normalised collision energy) mass spectrum of [GNA(Sec)GIAAFVQR]$^{2+}$, acquired in the tandem LC-MS analysis of the tryptic digest of PstS-Sel178. The peak at m/z = 1099.5 Th corresponds to the mass of the fragment y_9 modified by Sec.

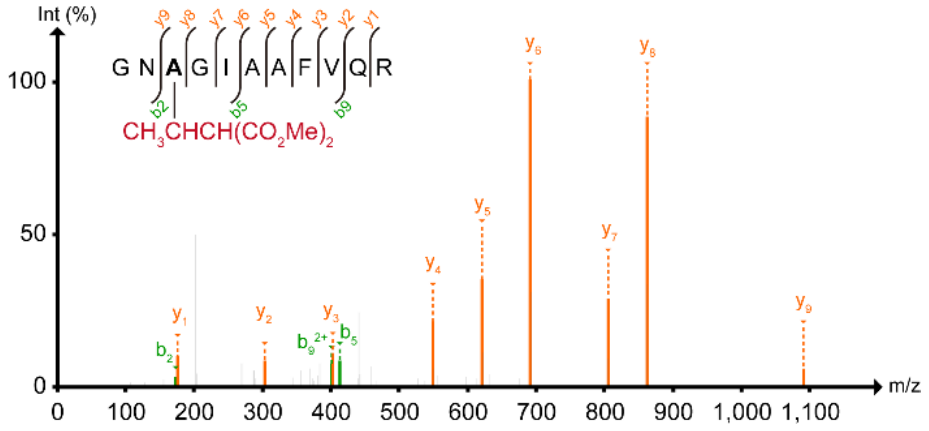

Supplementary Figure S6: MS/MS analysis of PstS-Mal-L178.

HCD (30% normalised collision energy) mass spectrum of [GNA($CH_3CHCH(CO_2Me)_2$)GIAAFVQR]$^{2+}$, acquired in the tandem LC-MS analysis of the tryptic digest of PstS-Sel178. The peak at m/z = 1090.5 Th corresponds to the mass of the fragment y_9 modified by $CH_3CHCH(CO_2Me)_2$.

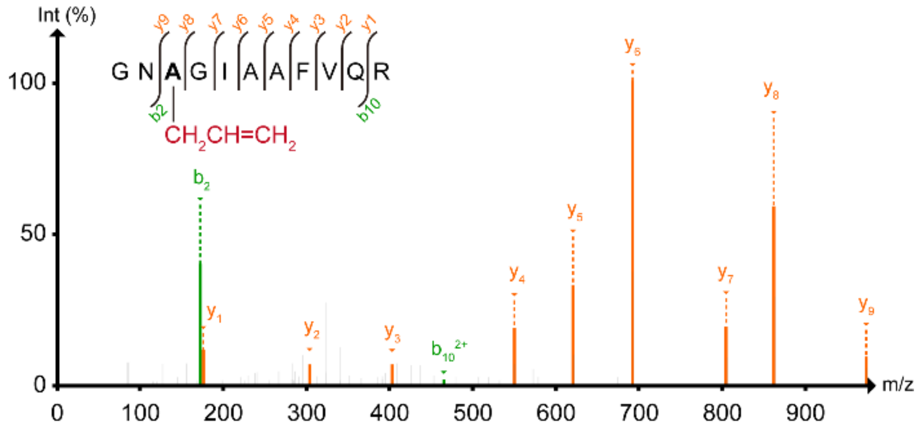

Supplementary Figure S7: MS/MS analysis of PstS-Hag178

HCD (30% normalised collision energy) mass spectrum of [GNA(CH$_2$CHCH$_2$)GIAAFVQR]$^{2+}$, acquired in the tandem LC-MS analysis of the tryptic digest of PstS-Hag178. The peak at m/z = 972.5 Th corresponds to the mass of the fragment y_9 modified by CH_2CHCH_2.

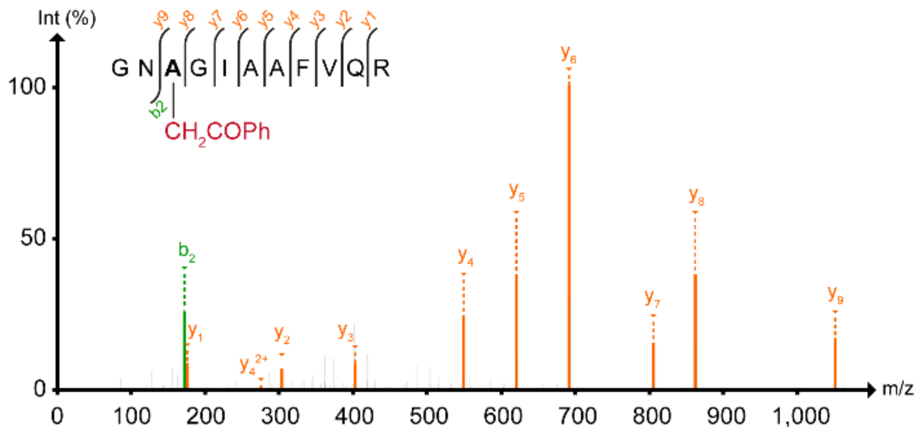

Supplementary Figure S8: MS/MS analysis of PstS-A(Ketone)178

HCD (30% normalised collision energy) mass spectrum of [GNA(CH$_2$COPh)GIAAFVQR]$^{2+}$, acquired in the tandem LC-MS analysis of the tryptic digest of PstS-A(Ketone)178. The peak at m/z = 1050.5 Th corresponds to the mass of the fragment y_9 modified by CH$_2$COPh.

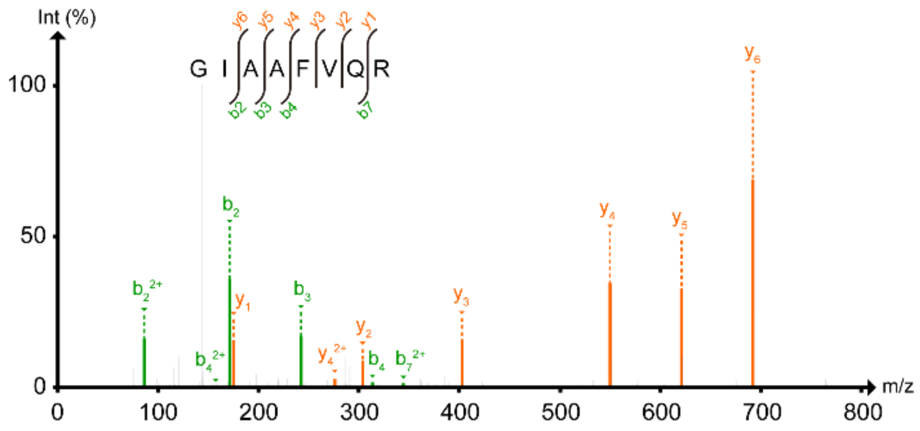

Supplementary Figure S9: MS/MS analysis of PstS-Lys178

HCD (30% normalised collision energy) mass spectrum of $[GIAAFVQR]^{2+}$, acquired in the tandem LC-MS analysis of the tryptic digest of PstS-Lys178. This tryptic peptide is the product of the specific proteolytic cleavage of the bond between L-A178K and glycine in PstS-Lys178.

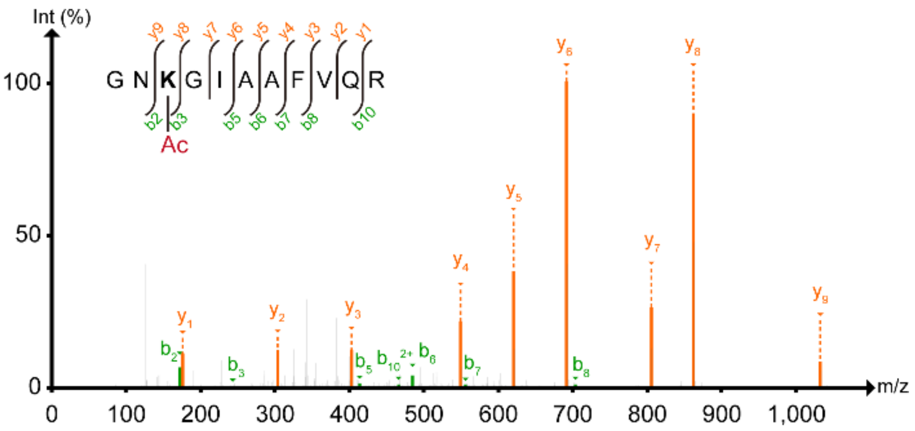

Supplementary Figure S10: MS/MS analysis of PstS-KAc178

HCD (30% normalised collision energy) mass spectrum of [GNK(Ac)GIAAFVQR]$^{2+}$, acquired in the tandem LC-MS analysis of the tryptic digest of PstS-KAc178. The peak at m/z = 1032.5 Th corresponds to the mass of the acetylated fragment y_9.

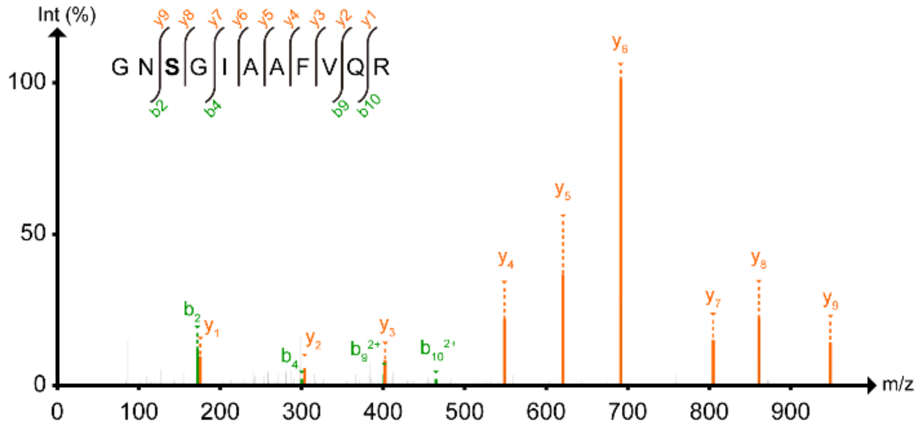

Supplementary Figure S11: MS/MS analysis of Psts-Ser178

HCD (30% normalised collision energy) mass spectrum of [GNSGIAAFVQR]$^{2+}$, acquired in the tandem LC-MS analysis of the tryptic digest of PstS-Ser178. The peak at m/z = 948.5 Th corresponds to the mass of the fragment y_9.

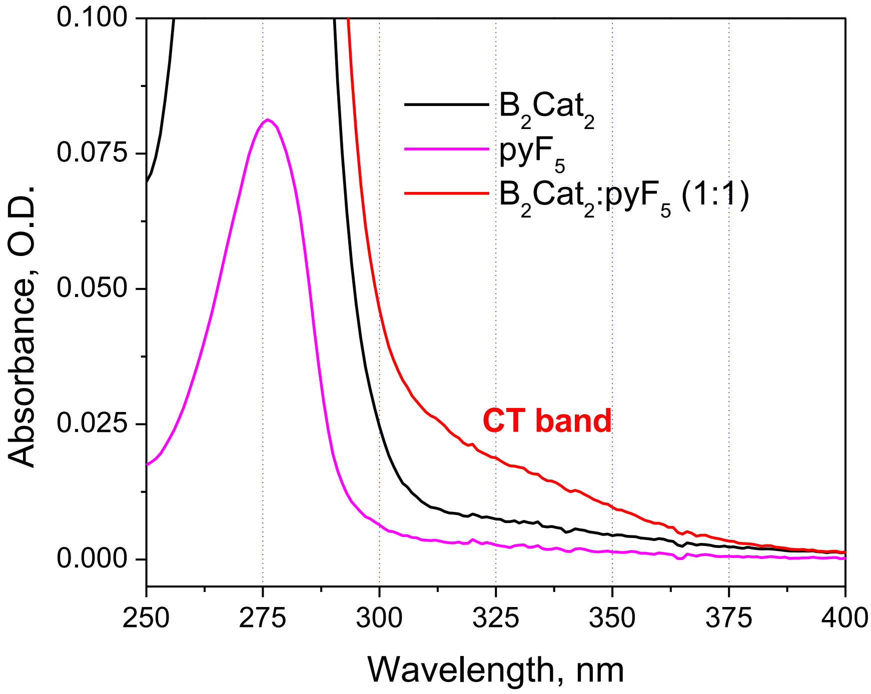

Supplementary Figure S12: Spectral signature of EDA complexation

Absorption spectra of bis(catecholato)diboron (B_2Cat_2, electron donor, 0.1 mM, in black) and pentafluoropyridine (pyF_5, electron acceptor, 0.1 mM, in magenta). The red trace represents 1:1 mixture of donor and acceptor in buffer solution (Tris, pH = 8.0). A weak charge transfer (CT) band lower in energy than the parent molecular transitions can be observed, which confirms EDA complexation.

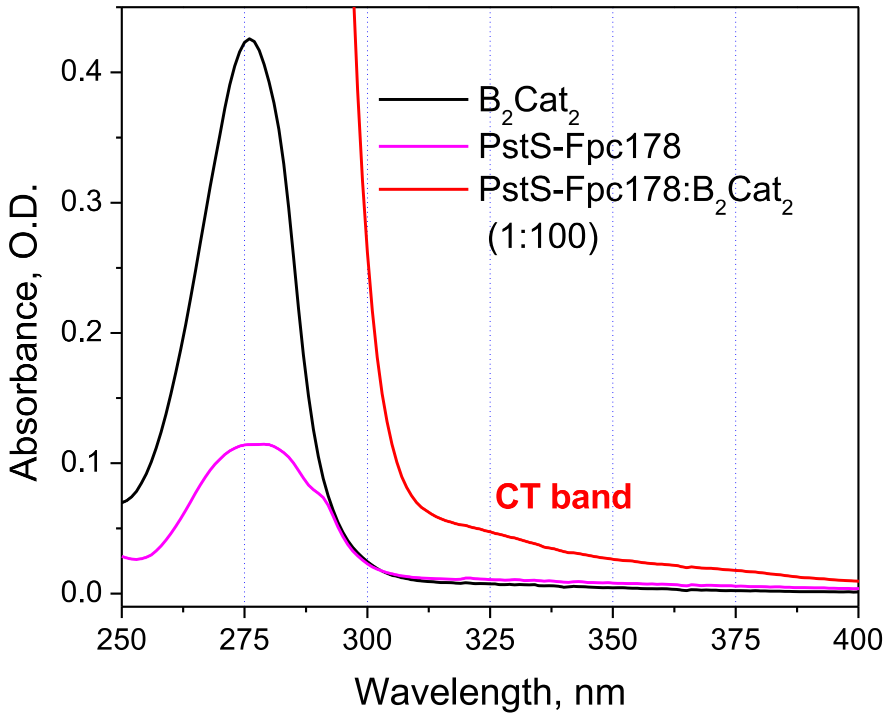

Supplementary Figure S13: Spectral signature of EDA complexation on protein

Absorption spectra of bis(catecholato)diboron (B_2Cat_2, electron donor, 0.1 mM, in black) and PstS-Fpc178 (protein with electron acceptor, 0.02 mM, in magenta). The red trace represents 1:100 mixture of donor and acceptor in buffer solution (Tris, pH = 8.0). A weak charge transfer (CT) band lower in energy than the parent molecular transitions can be observed, which confirms EDA complexation.

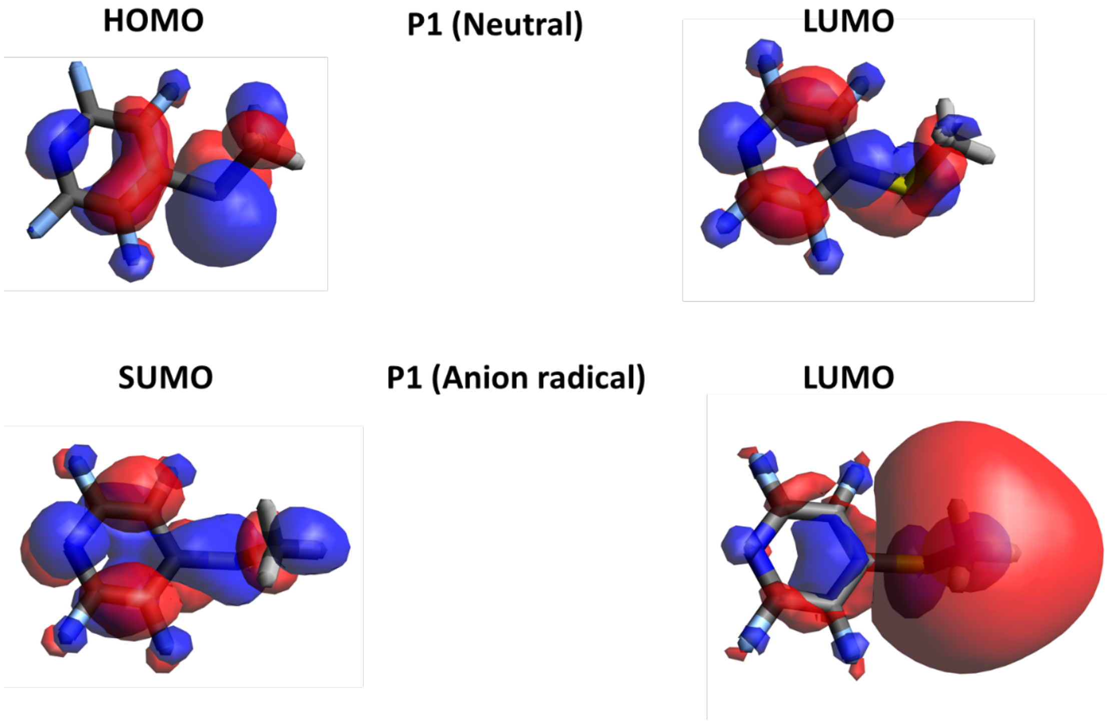

Supplementary Figure S14: Frontier molecular orbitals from DFT Calculations

The frontier molecular orbitals of P1 molecule is shown in its neutral and anion radical state. Molecular orbitals shown here are obtained by performing DFT calculations using B3LYP/6311G(d,p).

(a)

AAWKTNIK
DQKKPEQGTEVLK
DSSGKPLY
ETGNKVNYQGIGSSGGVK
FFDWAYKTGAK
GADWSKTFAQDLTNQK
GEDAWPITSTTFILIHK
GNA(178)GIAAFVQR
IKKWDDEAIAK
KPEQGTEVLK
KWDDEAIAK
LISADGKPVSPTEENFANAAK
LISADGKPVSPTEENFANAAKGADWSK
LNPGLKLPSQNIAVVR
LPGAIGYVEYAYAKQNNLAYTK
NNVGTGSTVKWPIGLGGK
QNNLAYTKLISADGKPVSPTEENFANAAK
SGELVLDGKTLGDIYLGK
TGAKQANDLDYASLPDSVVEQVR
TLGDIYLGKIK
TNIKDSSGKPLY
VNEEWKNNVGTGSTVK
VNEEWKNNVGTGSTVKWPIGLGGK
WADTYQKETGNK
WDDEAIAKLNPGLK

(b)

MEASLTGAGATFPAPVYAKWADTYQKETGNKVNYQGIGSSGGVKQII
ANTVDFGASDAPLSDEKLAQEGLFQFPTVIGGVVLAVNIPGLKSGELV
LDGKTLGDIYLGKIKKWDDEAIAKLNPGLKLPSQNIAVVRRADGSGTS
FVFTSYLAKVNEEWKNNVGTGSTVKWPIGLGGKGNAGIAAFVQRLP
GAIGYVEYAYAKQNNLAYTKLISADGKPVSPTEENFANAAKGADWSK
TFAQDLTNQKGEDAWPITSTTFILIHKDQKKPEQGTEVLKFFDWAYKT
GAKQANDLDYASLPDSVVEQVRAAWKTNIKDSSGKPLY

(c)

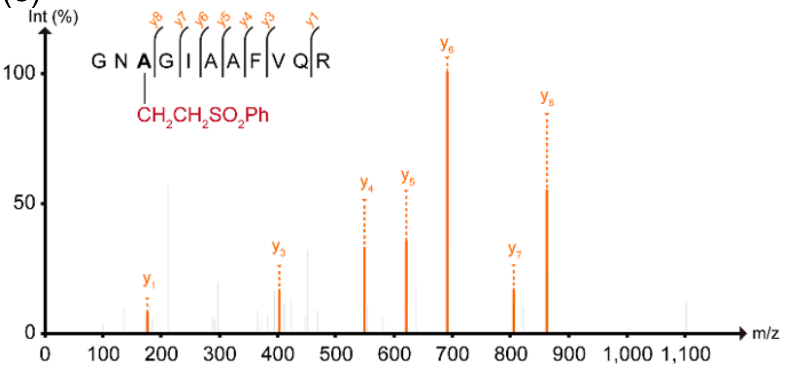

Supplementary Figure S15: MS/MS analysis of Psts-A(Sulphone)178

(**a**) A list of peptides with the mass shift corresponding (within the mass accuracy of 0.005 Da) to $CH_2CH_2SO_2Ph$ identified in the tryptic digest of PstS-A(Sulfone)178. Possible additional non-specific modification sites are labelled with the red colour. The modification of the lysine residues in these peptides led to missed cleavages by trypsin at these sites. (**b**) Coverage of the amino-acid sequence of PstS by peptides listed in (a). Modification sites are highlighted in red. (**c**) MS/MS spectrum for PstS$^{176\text{-}186}$ peptide in PstS-A(Sulfone)178.

(**a**)

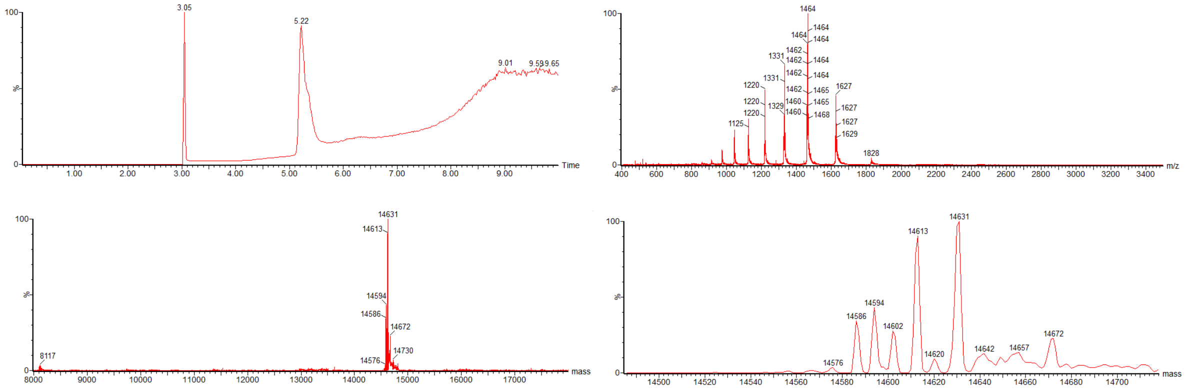

(**b**)

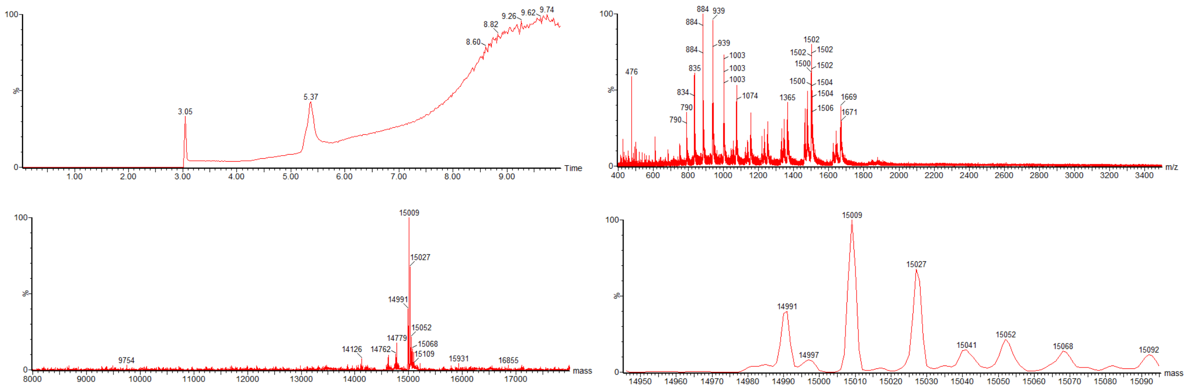

Supplementary Figure S16: Testing of Disulfide Compatibility in the Editing of Single Domain Ab cabVCAM to form cabVCAM-Bal118 and the Disruptive Effect of TCEP.

(**a**) Intact protein ESI-MS spectrum for the cabVCAM-Bal118 following treatment with Ellman's reagent for detection of any free thiols [Tris buffer (20 mM, NaCl 150 mM, pH 8.0), Ellman's reagent (20 mM in DMSO, 20 equiv, 2.7 µL), 37 ˚ C for 30 min]. The calculated masses [m/z = 14631 (Bal), 14613 (Bal- H_2O), 14695 (Bal- $2H_2O$] and observed masses [m/z = 14631 (Bal), 14613 (Bal- H_2O), 14694 (Bal- $2H_2O$)] indicate editing without disruption of the internal disulfide.

(**b**) To test the comparable disruption that would arise from phosphines such as TCEP cabVCAM-Bal118 was also analyzed in the same way after treatment with TCEP [54 µM, 50 µL in Tris buffer (20 mM, NaCl 150 mM, pH 8.0), TCEP·HCl (20 mM in H_2O, 20 equiv, 2.7 µL), 37 ˚C for 30 min]. Following treatment with Ellman's reagent [DTNB, 20 mM in DMSO, 40 equiv, 5.4 µL, incubated at 37 ˚ C for a further 30 min] intact protein ESI-MS spectra indicate clear disruption of the internal disulfide and subsequent detection by Ellman's; calculated masses: m/z = 15027 (Bal+DTNB), 15009 (Bal+DTNB-H_2O), 14991

(Bal+DTNB-2H_2O); observed masses m/z = 15027 (Bal+DTNB), 15009 (Bal+DTNB-H_2O), 14991 (Bal+DTNB-2H_2O)].

(a)

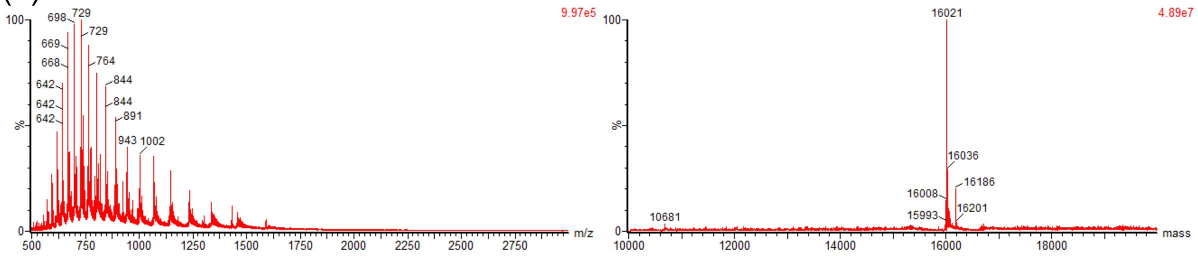

(b)

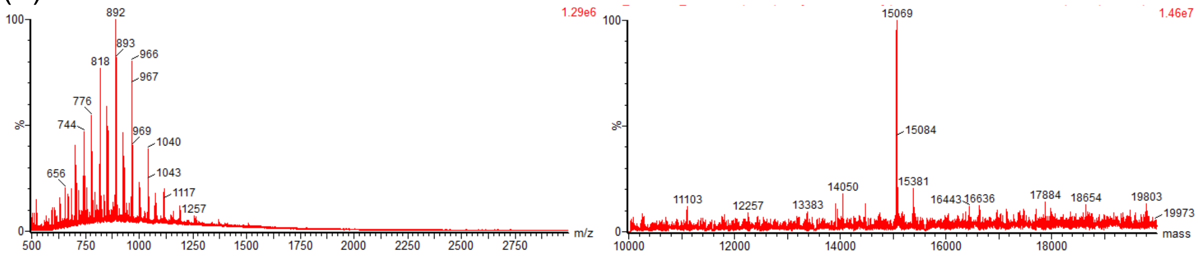

(c)

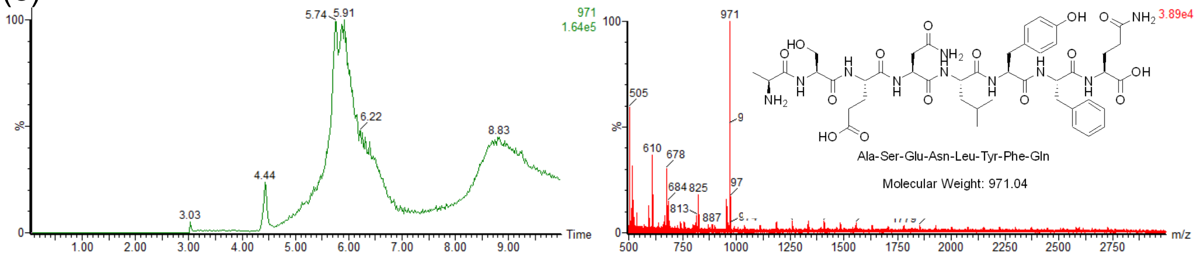

(d)

(i)

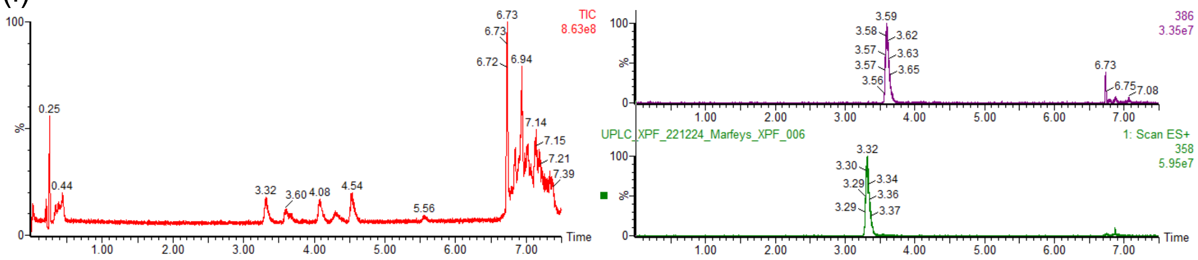

(ii)

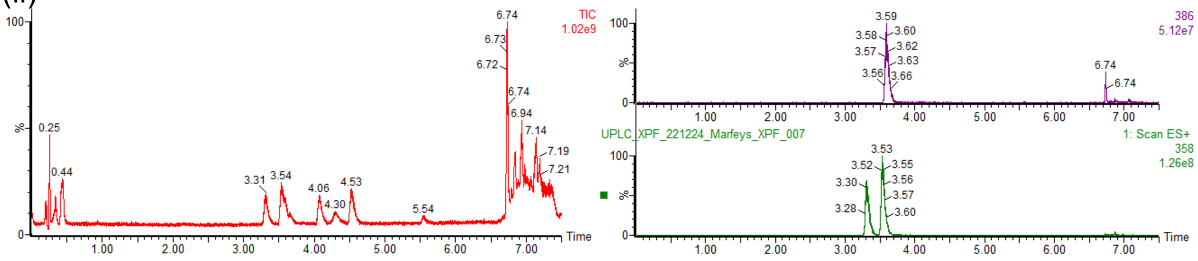

(iii)

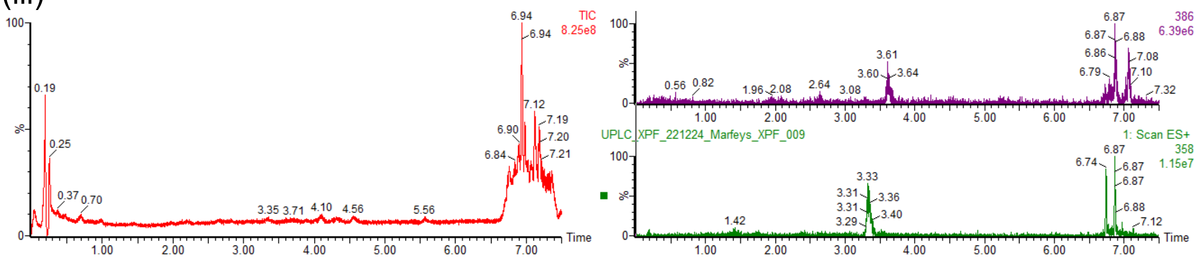

(iv)

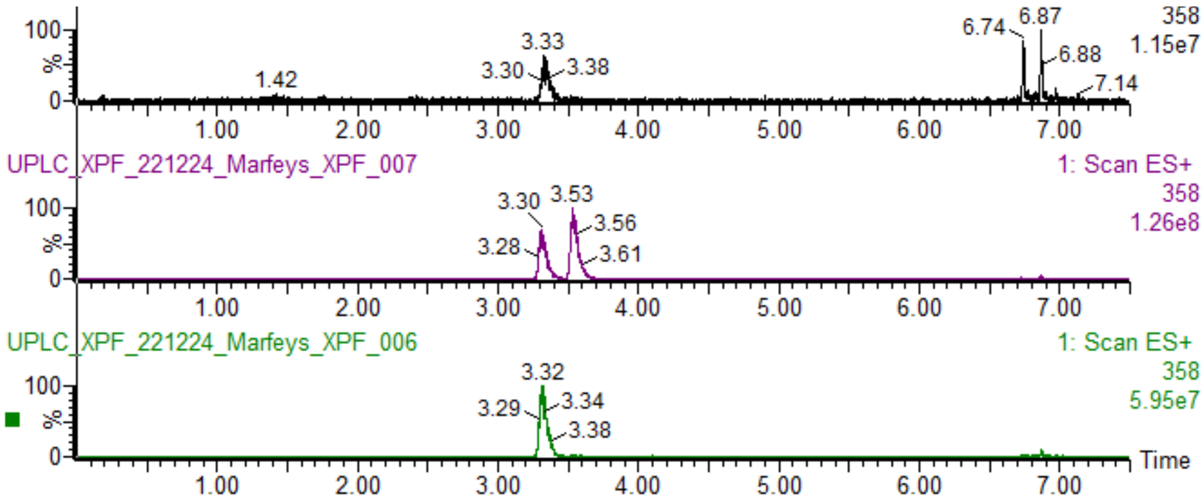

(v)

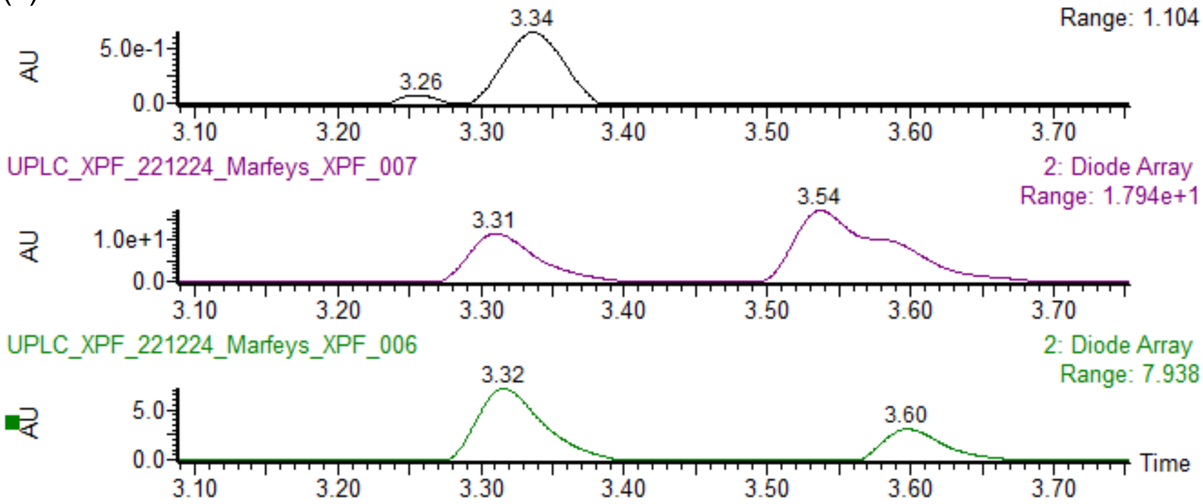

Supplementary Figure S17: Testing of Marfey's Analysis for Detection of Configuration in edited TEV-HistoneH3-Ser2 protein.

(**a**) Intact protein ES-MS spectra of TEV-HistoneH3-Ser2, ion series raw (**left**), deconvoluted total mass spectrum (**right**).

(**b**) TEV-HistoneH3-Ser2 was cleaved using TEV protease to release the N-terminal fragment shown in (c). Intact protein ES-MS spectra of cleaved-TEV-HistoneH3-Ser2, ion series raw (**left**), deconvoluted total mass spectrum (**right**) confirmed cleavage.

(**c**) Extracted ion chromatogram (EIC) of the isolated, cleaved N-terminal peptide from treatment with TEV protease containing the relevant edited Ser2 [EIC for m/z 971, **left**; with corresponding mass spectrum for retention time = 253 - 262 s (see methods for further details).

(**d**) UPLC analyses of Marfey's analyses on N-terminal peptide.

(i) Analysis of mixture of L-FDAA derivatives of the residues found in the N-terminal peptide as standards [total ion chromatogram (TIC) of control mixture, in red (**left**); EIC for

L-FDAA-L-Asp, m/z = 386 m/z, in purple (**right, top**), 3.59 min; EIC for L-FDAA-L-Ser, m/z = 358, in green (**right, bottom**), 3.32 min]

(ii) Analysis of the sample control mixture additionally spiked with L-FDAA-D-Ser (0.1 mM) [total ion chromatogram (TIC) of spiked mixture, in red (**left**); EIC for L-FDAA-L-Asp, m/z = 386 m/z, in purple (**right, top**), 3.59 min; EIC for L-FDAA-L-Ser with L-FDAA-D-Ser, m/z = 358, in green (**right, bottom**), 3.32 min L-FDAA-L-Ser, 3.53 L-FDAA-D-Ser]

(iii) Analysis of N-terminal peptide [total ion chromatogram (TIC) of sample in red (**left**); EIC for L-FDAA-L-Asp, m/z = 386 m/z, in purple (**right, top**), 3.61 min; EIC for L-FDAA-D/L-Ser m/z = 358, in green (**right, bottom**) – only L-FDAA-L-Ser at 3.32 min is observed in the analyzed sample]

(iv) Comparison of the EICs for FDAA-Ser, m/z = 358 [control mixture with only L-FDAA-L-Ser in black (**top**), spiked mixture with L-FDAA-L-Ser and L-FDAA-D-Ser in purple (**middle**); analysis of N-terminal peptide in green (**bottom**) – only L-FDAA-L-Ser at 3.32 min is observed in the analyzed sample]

(v) Diode Array UV-Vis chromatogram comparison of the same samples [control mixture with L-FDAA-L-Ser and in black (**top**), spiked mixture with L-FDAA-L-Ser and L-FDAA-D-Ser in purple (**middle**); analysis of N-terminal peptide in green (**bottom**) – L-FDAA-L-Ser at 3.32 min is observed in the analyzed sample; note L-FDAA-L-Asp appears as a shoulder in both the spiked and analyzed sample at 3.60 min]

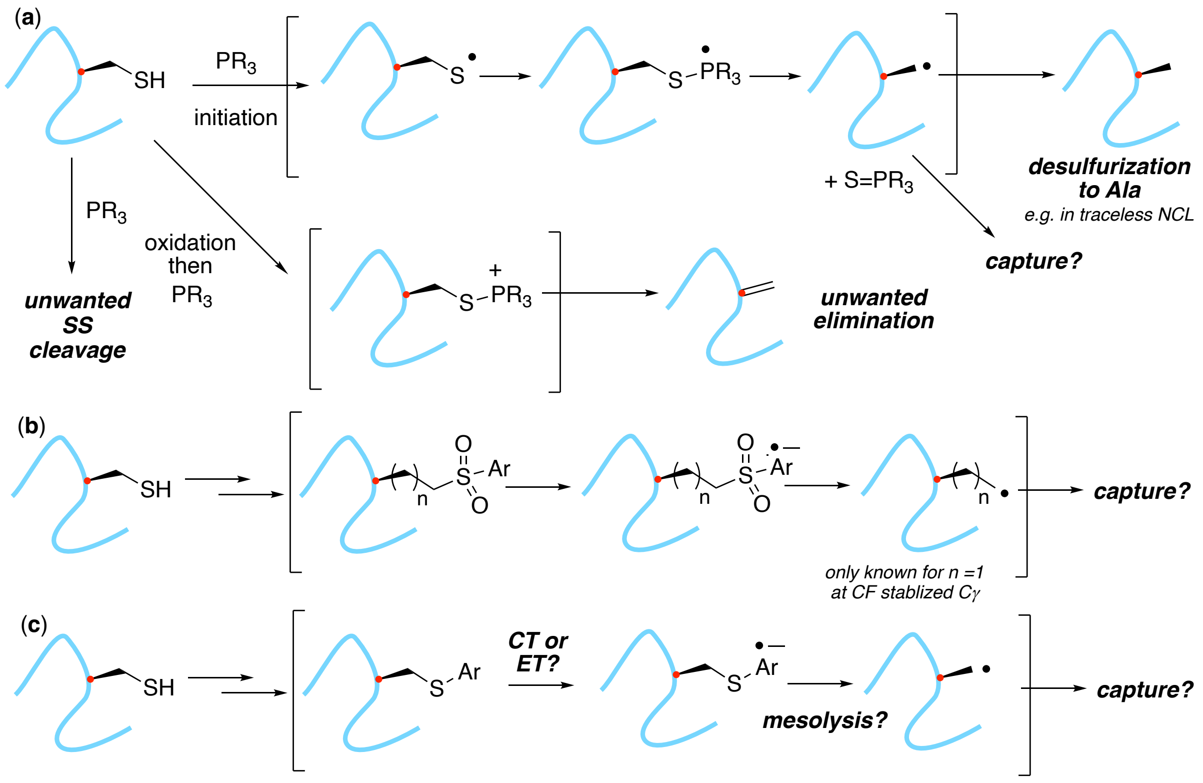

Supplementary Figure S18: Possible Modes of C–S Homolysis to Putative On-protein C• Side-chain Radical Generation for Stereoretentive Sidechain Editing.

It has been proposed for over 60 years that alanyl radicals may be intermediates in Cys desulfurization reactions.[1,2]

(**a**, top) Such reactions are now commonly exploited in so-called 'traceless native chemical ligation' [3,4,5] to convert Cys to desulfurized Ala residues. In peptidic systems alanyl-radicals have shown promise by taking advantage of phosphine to activate the C_β-S_γ bond.[6,7] Such prior strategies for desulfurization at cysteine, cystine or selenenylcysteines proceed via a seemingly complex or likely multiple-manifold process[8] involving the likely intermediate formation of thiophosphoranyl radical adducts as precursors to C• radicals formed upon β-scission.

(**a**, bottom) The requirement in these systems for use of phosphines or other P(III) reagents, which are strongly reducing, effectively precludes more general use in typical protein systems since these are commonly used to disrupt disulfides (**a**, bottom left) (e.g. TCEP, see also **Supplementary Figure S16**). We have shown that eliminative mechanisms to Dha may compete in some phosphine mediated desulfurization manifolds thereby raising the potential for loss of stereochemistry or side-reaction (**a**, bottom right).[9]

(**b**) On-protein C• radicals, when stabilised by α-fluoro-substitution as $C(F)_n$•, allow reactivity that enables C–Se, C–O and C–C bond formation[10] but require the creation of unnatural (e.g. fluorine substituted) sidechain precursors.

(**c**) Alternative methods for tuning the radical scission potential of the Cβ–S bond are explored in this manuscript. The presence of electron-withdrawing substituents on S is known to enhance C–S bond cleavage via homolytic and mesolytic manifolds.[11,12] In reductive initiation this may stabilize appropriate radical anion intermediates formed upon single-electron transfer (SET) / charge transfer (CT) and/or thiolates in mesolysis / homolysis.

Supplementary Tables

Supplementary Table S1: Optimisation of chemical introduction of tetrafluoropyridyl-cysteine (Fpc) into proteins

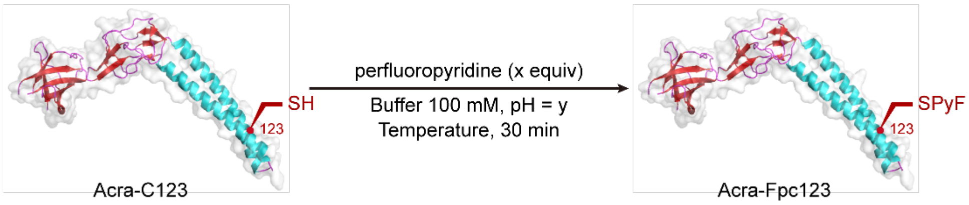

entry	Buffer	x	y	Temperature (°C)	Conversion
1	NaPi	100	8.0	4	75%
2	NaPi	200	8.0	4	84%
3	NaPi	400	8.0	4	90%
4	NaPi	200	8.0	25	>98% conversion
5	NaPi	200	7.4	25	>98% conversion
6	NaPi	200	7.0	25	60%
7	NaPi	200	6.0	25	Trace
8	Tricine	200	7.4	25	>98% conversion
9	Tris	200	7.4	25	>98% conversion

General procedure: AcrA-Cys123 (20 µM, 50 µL, in buffer), perfluoropyridine (x equiv, 100 mM, in DMSO), were mixed and incubated at 4 °C or 25 °C for 30 min. The reaction mixture was then analysed by LC-MS.

Supplementary Table S2: Optimisation of light-mediated C_β-S_γ bond cleavage

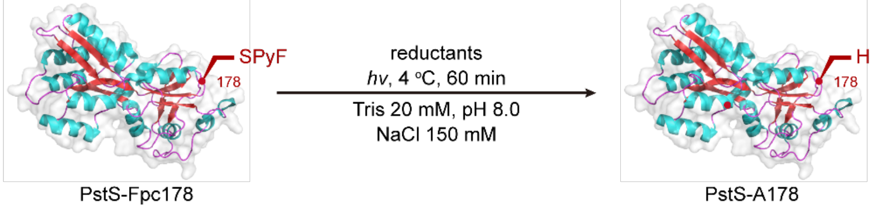

Entry	Reductants	hυ	Conversion
1	4-Me-PhSH (100 equiv)	365 nm	>98% conversion
2	4-Me-PhSH (100 equiv)	385 nm	0%
3	4-Me-PhSH (100 equiv)	405 nm	0%
4	4-Me-PhSH (100 equiv)	420 nm	0%
5	4-Me-PhSH (100 equiv)	445 nm	0%
6	2-Cl-4-F-PhSH (100 equiv)	365 nm	>98% conversion
7	Ir(dtppy)(bpy)$_2$BF$_4$ (10 equiv), FeSO$_4$ (200 equiv)	365 nm	90%
8	Ir(dtppy)(bpy)$_2$BF$_4$ (10 equiv), FeSO$_4$ (200 equiv)	385 nm	90%
9	Ir(dtppy)(bpy)$_2$BF$_4$ (10 equiv), FeSO$_4$ (200 equiv)	405 nm	71%
10	Ir(dtppy)(bpy)$_2$BF$_4$ (10 equiv), FeSO$_4$ (200 equiv)	420 nm	77%
11	Ir(dtppy)(bpy)$_2$BF$_4$ (10 equiv), FeSO$_4$ (200 equiv)	445 nm	35%
12	Ru(bpy)$_3$Cl$_2$ (10 equiv), FeSO$_4$ (200 equiv)	365 nm	52%
13	Ru(bpy)$_3$Cl$_2$ (10 equiv), FeSO$_4$ (200 equiv)	385 nm	63%
14	Ru(bpy)$_3$Cl$_2$ (10 equiv), FeSO$_4$ (200 equiv)	405 nm	66%
15	Ru(bpy)$_3$Cl$_2$ (10 equiv), FeSO$_4$ (200 equiv)	420 nm	75%
16	Ru(bpy)$_3$Cl$_2$ (10 equiv), FeSO$_4$ (200 equiv)	445 nm	51%
17	B$_2$Cat$_2$ (100 equiv)	365 nm	>98% conversion[a]
18	B$_2$Cat$_2$ (100 equiv)	385 nm	0%
19	B$_2$Cat$_2$ (100 equiv)	405 nm	0%

20	B_2Cat_2 (100 equiv)	420 nm	0%
21	B_2Cat_2 (100 equiv)	445 nm	0%

In glovebox PstS-Fpc178 (15 μM, 50 μL), reductants, were mixed and irradiated with *hυ* at 4 ^{o}C for 60 min. The reaction mixture was then analysed by LC-MS.

[a] 9% conversion to PstS-A178; 91% conversion to PstS-Bal178.

Supplementary Methods

General Experimental Procedures

Chemicals and solvents were purchased from Sigma-Aldrich UK, Acros UK, Alfa Aesar UK, Carbosynth, Fluorochem or Fischer UK and were used as delivered unless stated otherwise. Thin layer chromatography (TLC) was carried out using plastic 0.20 mm Polygram® Silg/UV254 plates that were dried using a heat gun and visualised under UV (λmax 254 nm or 366 nm) or by use of anisaldehyde, potassium permanganate, sulfuric acid or vanillin dip. Flash column chromatography was performed using Geduran® Si 60.8.2 or a Teldyne Flash Purification System with either Kinesis Telos or Biotage Snap columns.

NMR Spectroscopy General: Deuterated solvents were used as the lock and the residual protonated solvent as the internal reference peak. Spectra were analysed using MestReNova.

Glovebox Usage: Anaerobic atmosphere was achieved using a Belle Technology glovebox equipped with the BASF R3-11G catalyst. The oxygen level was measured below 6 ppm during all reactions.

Photobox usage: Reactions were performed on the Zinsser Analytic off-deck irradiation system with two reaction positions irradiated by Lumidox II 96-LED arrays. 96-position, open-bottom Desyre reaction blocks equipped with several 1.2 mL vials were placed in the irradiation box, agitated at 400 rpm, and irradiated with 365 nm light at 230 mW per well for 60 mins. Cooling of reaction positions to 4 °C was provided by an off-deck circulating cooler.

Raw data is deposited at doi: 10.5281/zenodo.7011026

Protein Mass Spectrometry

Protein samples were analysed on Waters Xevo G2-XS QTof mass spectrometers equipped with a Waters Acquity UPLC. Separation was achieved using a Thermo Scientific ProSwift RP-2H monolithic column (4.6 mm × 50 mm) using water + 0.1% formic acid (solvent A) and acetonitrile + 0.1% formic acid (solvent B) as mobile phase at a flow rate of 0.3 ml/min and running a 10-min linear gradient as follows: 5% solvent B for 1 min, 5 to 95% solvent B over 6 min, 95 to 5% solvent B over 1 min, and 5% solvent B for 2 min. Spectra were deconvoluted using MassLynx 4.1 (Waters) and the "MaxEnt1" deconvolution algorithm with the following settings: resolution: 1.0 Da per channel; damage model: uniform Gaussian; width

at half height: 0.4 Da; minimum intensity ratios: 33% (left) and 33% (right); and iterate to convergence. Con-versions were calculated from peak intensities.

Protein digestion and analysis by MS/MS

For in-solution proteolytic digestion, samples were dissolved in 100 mM ammonium bicarbonate, reduced with 10 mM tris(2-carboxyethyl)phosphine (Thermo Fisher) at 56 °C for 30 min and alkylated with 30 mM 2-chloroacetamide (Sigma Aldrich) at room temperature for 30 min in the dark. Trypsin (Pierce) was added to each sample for an overnight incubation at 37 °C with 1:25 trypsin:protein ratio (w/w). The samples were desalted by Oasis HLB cartridges (Waters), dried, and reconstituted in water containing 5% formic acid, 5% DMSO right before the LC-MS analysis.

For in-gel proteolytic digestion (PstS-Bal178 and PstS-TEMPO-A178), samples were resolved via SDS-PAGE and stained with InstantBlue® (Abcam). After destaining in MilliQ® H_2O for 20 min, the corresponding bands were excised and cut into small cubes. The gels were extensively destained twice with 50 mM NH_4HCO_3/MeCN (1:1, v/v) at room temperature for 10 min. After washing with 50 mM NH_4HCO_3 for 10 min, samples were reduced with 10 mM tris(2-carboxyethyl)phosphine (Thermo Fisher) at room temperature for 60 min and alkylated with 30 mM 2-chloroacetamide (Sigma Aldrich) at room temperature for 30 min in the dark. The gels were washed twice with 50 mM NH_4HCO_3/MeCN (1:1, v/v) at room temperature for 10 min. In turn, the gels were dehydrated with MeCN and dried under air for 10 min before overnight trypsin (Pierce) digestion in 50 mM NH_4HCO_3 at 37 °C with 1:25 trypsin:protein ratio (w/w).

Samples were subjected to LC-MS/MS using a UltiMate 3000 nanoUHPLC system (Thermo Fisher Scientific) coupled to an Orbitrap Fusion Lumos (Thermo Fisher Scientific). The peptides were trapped on a C18 PepMap100 pre-column (300 µm i.d. x 5 mm, 100 Å, Thermo Fisher Scientific) using solvent A (0.1% formic acid in water), then separated on an in-house packed analytical column (75 µm i.d. x 50 cm in-house packed with ReproSil Gold 120 C18, 1.9 µm, Dr. Maisch GmbH) with a gradient of 12% to 40% B (0.1% formic acid in acetonitrile) over 15 min at a flow rate of 200 nL/min. Full scan MS spectra were acquired in the Orbitrap (scan range 350-1400 m/z, resolution 60000, AGC target 1200000). The 20 most intense peaks were selected for HCD fragmentation at 30% of normalised collision energy and with a resolution 7500, AGC target 20000.

Spectra were searched using FragPipe (v18.0) MSFragger 3.5[13] with standard ‘open’ search settings against database (PDB ID 2abh with mutation D178A). Data was filtered using the inbuilt tools within FragPipe to an FDR of below 1%. Modified peptides were discerned by filtering the resulting dataset using the expected changes in mass caused by each modification.

General procedure 1: Cys-tetrafluoropyridylsulfide (Fpc) formation in proteins

To a solution of Protein (1.0 mL, 1 mg/mL, 1.00 equiv) NaPi buffer (100 mM, pH 7.4)[a] was added prepared stock solutions of perfluoropyridine (1 M in DMSO, 100 equiv). The mixture was shaken for 30 min at room temperature. The protein was desalted by passing through a GE MiniTrap G-25 column pre-equilibrated with Tris buffer (20 mM, NaCl 150 mM, pH 8.0) according to the manufacturer's instructions.

[a]For protein PstS-D57C, PstS-D103C, PstS-A197C, NaPi buffer (100 mM, 3 M Gdn·HCl, pH 7.4) was used; for histone proteins, tricine buffer (100 mM, 3 M Gdn·HCl, pH 7.4) was used.

General procedure 2: Alanyl radical trapped by HAT

In glovebox PstS-Fpc178 (15 μM, 50 μL) in Tris buffer (20 mM, NaCl 150 mM, pH 8.0), 4-Me-PhSH (50 mM in DMSO, 100 equiv, 1.5 μL) were mixed and irradiated with 365 nm light at 4 °C for 60 min. The reaction mixture was then analysed by LC-MS.

General procedure 3: Alanyl radical trapped by TEMPO

In glovebox PstS-Fpc178 (15 μM, 50 μL) in Tris buffer (20 mM, NaCl 150 mM, pH 8.0), 4-Me-PhSH (50 mM in DMSO, 100 equiv, 1.5 μL), TEMPO (100 mM in DMSO, 200 equiv, 1.5 μL) were mixed and irradiated with 365 nm light at 4 °C for 60 min. The reaction mixture was then analysed by LC-MS.

General procedure 4: Alanyl radical trapped by diselenides

In glovebox PstS-Fpc178 (15 μM, 50 μL) in Tris buffer (20 mM, NaCl 150 mM, pH 8.0), 2,6-di-Cl-PhSH[b] (50 mM in DMSO, 100 equiv, 1.5 μL), diselenides (100 mM in DMSO, 200 equiv, 1.5 μL) were mixed and irradiated with 365 nm light at 4 °C for 60 min. The reaction mixture was then analysed by LC-MS.

[b]For PhSeSePh, 2,6-di-Cl-PhSH (50 mM in DMSO, 200 equiv, 3.0 equiv) was used.

General procedure 5: Alanyl radical trapped by alkenes

In glovebox PstS-Fpc178 (15 μM, 50 μL) in Tris buffer (20 mM, NaCl 150 mM, pH 8.0), 2-Cl-6-F-PhSH (50 mM in DMSO, 100 equiv, 1.5 μL), alkenes[c] (100 mM in DMSO, 200 equiv,

1.5 μL) were mixed and irradiated with 365 nm light at 4 °C for 60 min. The reaction mixture was then analysed by LC-MS.

[c]For allylic amine and 1-phenyl-1-trimethylsiloxyethylene, 2,6-di-Cl-PhSH (50 equiv) was used. For allylic phenyl sulfone (50 mM in DMSO, 100 equiv, 1.5 μL), 2-Cl-6-F-PhSH (50 mM in DMSO, 200 equiv, 3.0 μL) was used.

General procedure 6: Alanyl radical trapped by B_2Cat_2.

In glovebox PstS-Fpc178 (15 μM, 50 μL) in Tris buffer (20 mM, NaCl 150 mM, pH 8.0), B_2Cat_2 (100 mM in H_2O, 1000 equiv, 7.5 μL), were mixed and irradiated with 365 nm light at 4 °C for 60 min. The reaction mixture was then analysed by LC-MS.

General procedure 7: Alanyl radical formally trapped by O_2.

In air PstS-Fpc178 (15 μM, 50 μL) in Tris buffer (20 mM, NaCl 150 mM, pH 8.0), B_2Cat_2 (100 mM in H_2O, 1000 equiv, 7.5 μL), were mixed and irradiated with 365 nm light at 4 °C for 60 min. The reaction mixture was then analysed by LC-MS.

General procedure 8: Chemical introduction of boronoalanine (Bal) into proteins.

Cys-tetrafluoropyridylsulfide containing proteins were prepared *via* general procedure 1 without desalting. The mixture was directly used for the following borylation. In glovebox Cys-tetrafluoropyridylsulfide containing proteins (15 μM, 50 μL) in NaPi buffer (100 mM, pH 7.4), B_2Cat_2 (100 mM in H_2O, 1000 equiv, 7.5 μL), were mixed and irradiated with 365 nm light at 4 °C for 60 min. The reaction mixture was then analysed by LC-MS.

Histone H3-Fpc2, -Fpc4, -Fpc9, -Fpc18, -Fpc27, Histone H4-Fpc20: protein expression, purification and Fpc generation

Histone proteins expression and purification

Histone proteins were expressed and purified following a previously published procedure.[14]

Histone H3-K4C

ARTCQTARKSTGGKAPRKQLATKAARKSAPATGGVKKPHRYRPGTVALREIRRYQK STELLIRKLPFQRLVREIAQDFKTDLRFQSSAVMALQEASEAYLVALFEDTNLAAIHA KRVTIM PKDIQLARRIRGERA

Calculated mass = 15214

Observed mass = 15214

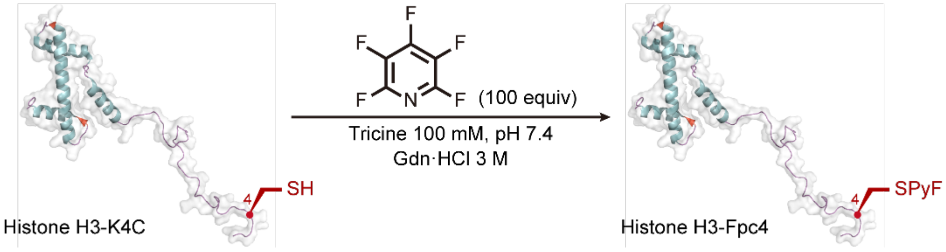

The Cys-tetrafluoropyridylsulfide-containing histone H3-Fpc4 was prepared according to general procedure 1.

ESI-MS spectrum for the modified histone is shown below.

Calculated mass = 15363

Observed mass = 15363

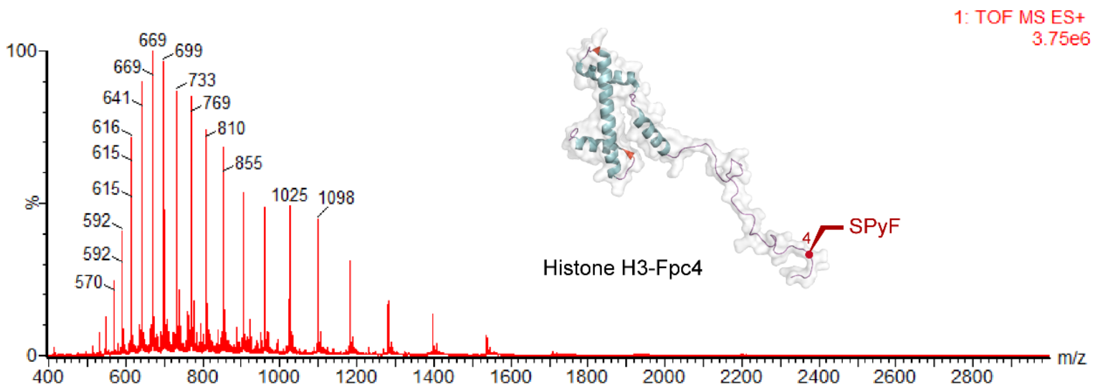

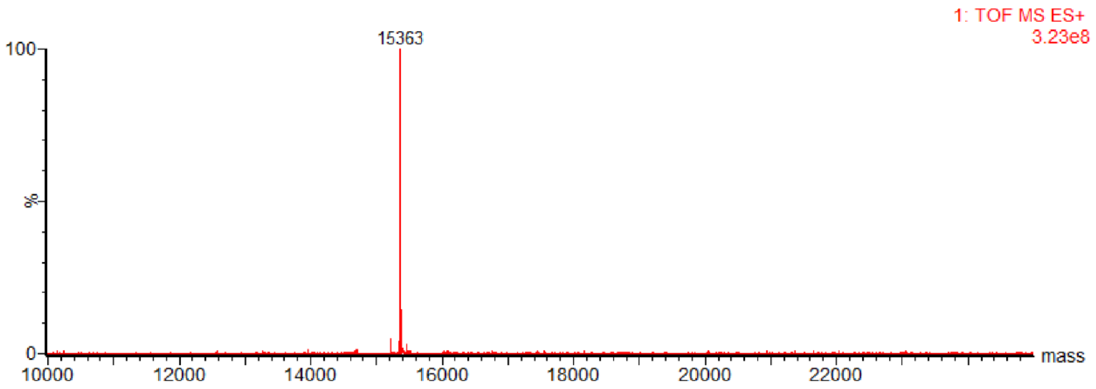

Histone H3-K9C

ARTKQTARCSTGGKAPRKQLATKAARKSAPATGGVKKPHRYRPGTVALREIRRYQK
STELLIRKLPFQRLVREIAQDFKTDLRFQSSAVMALQEASEAYLVALFEDTNLAAIHA
KRVTIM PKDIQLARRIRGERA

Calculated mass = 15214

Observed mass = 15214

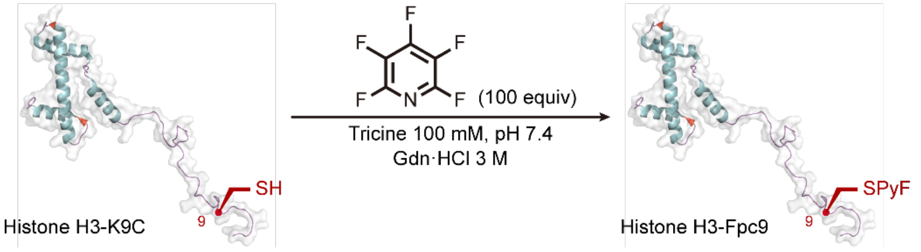

The Cys-tetrafluoropyridylsulfide-containing histone H3-Fpc9 was prepared according to general procedure 1.

ESI-MS spectrum for the modified histone is shown below.

Calculated mass = 15363

Observed mass = 15363

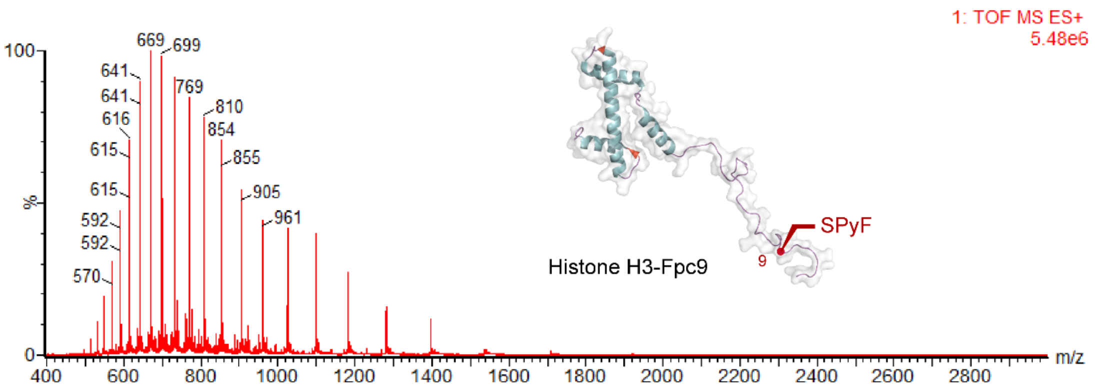

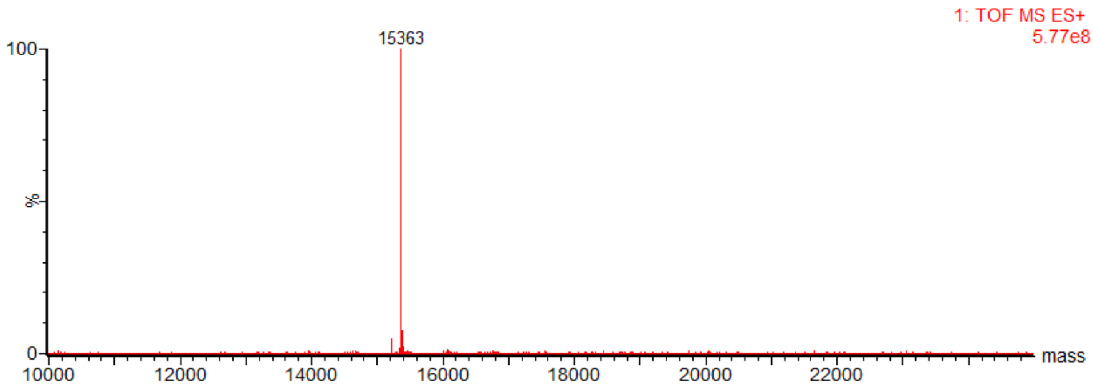

Histone H3-K18C

ARTKQTARKSTGGKAPRCQLATKAARKSAPATGGVKKPHRYRPGTVALREIRRYQK
STELLIRKLPFQRLVREIAQDFKTDLRFQSSAVMALQEASEAYLVALFEDTNLAAIHA
KRVTIM PKDIQLARRIRGERA

Calculated mass = 15214

Observed mass = 15214

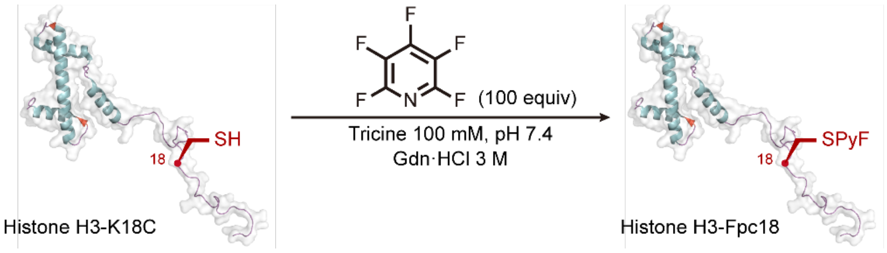

The Cys-tetrafluoropyridylsulfide-containing histone H3-Fpc18 was prepared according to general procedure 1.

ESI-MS spectrum for the modified histone is shown below.

Calculated mass = 15363

Observed mass = 15364

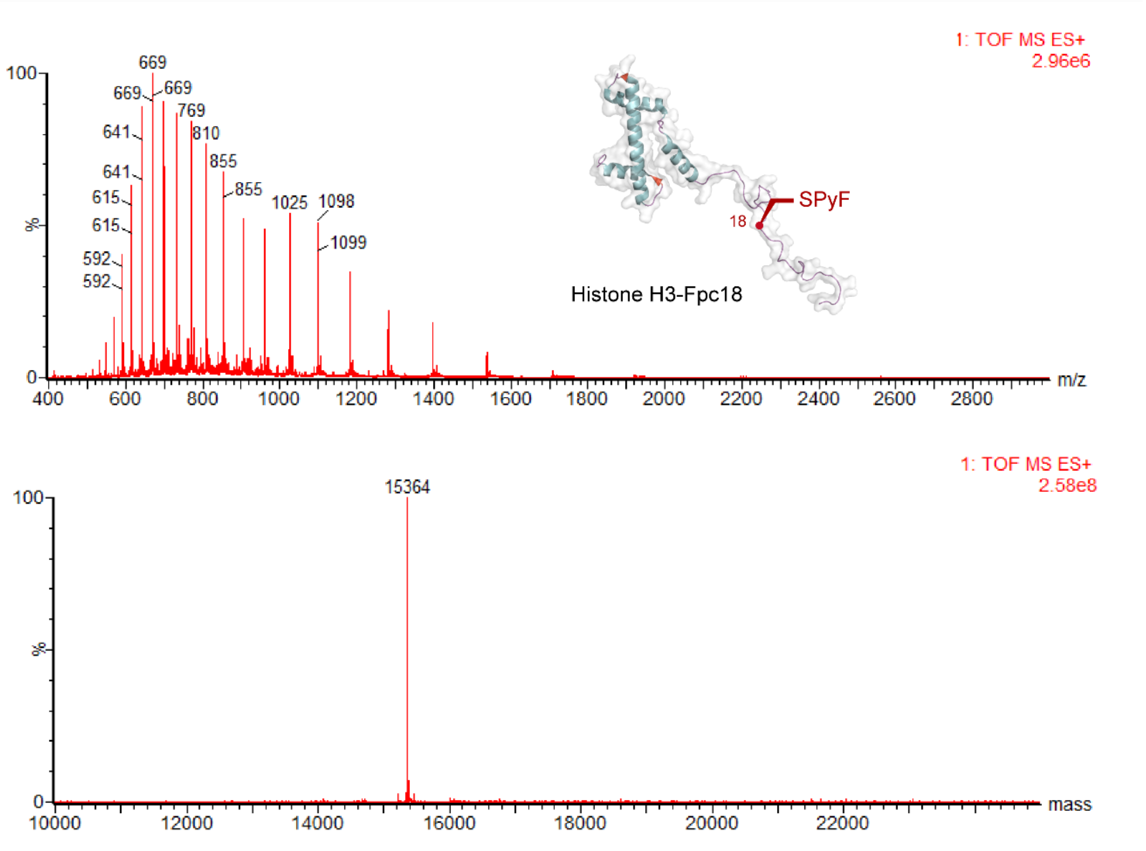

Histone H3-K27C

ARTKQTARKSTGGKAPRKQLATKAARCSAPATGGVKKPHRYRPGTVALREIRRYQK STELLIRKLPFQRLVREIAQDFKTDLRFQSSAVMALQEASEAYLVALFEDTNLAAIHA KRVTIM PKDIQLARRIRGERA

Calculated mass = 15214

Observed mass = 15214

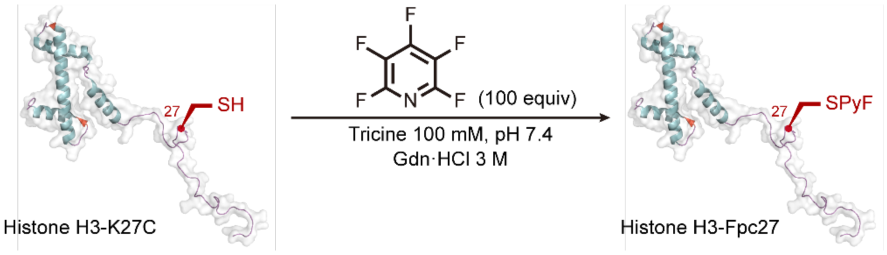

The Cys-tetrafluoropyridylsulfide-containing histone H3-Fpc27 was prepared according to general procedure 1.

ESI-MS spectrum for the modified histone is shown below.

Calculated mass = 15363

Observed mass = 15363

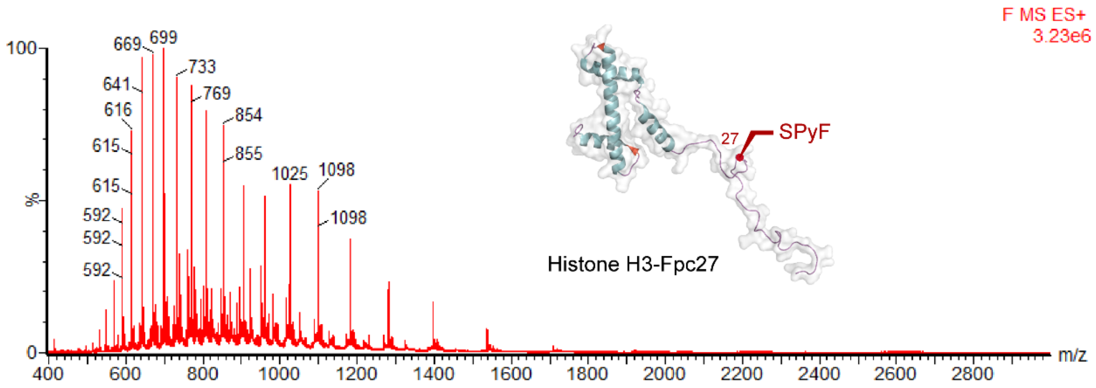

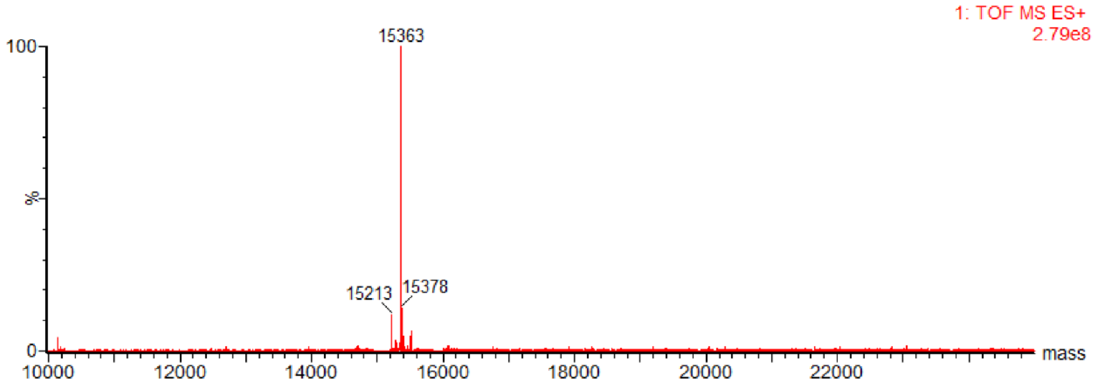

Histone H3-R2C

ACENLYFQGTKQTARKSTGGKAPRKQLATKAARKSAPATGGVKKPHRYRPGTVAL

REIRRYQKSTELLIRKLPFQRLVREIAQDFKTDLRFQSSAVMALQEASEAYLVALFED

TNLAAIHAKRVTIMPKDIQLARRIRGERA

Calculated mass = 16038

Observed mass = 16038

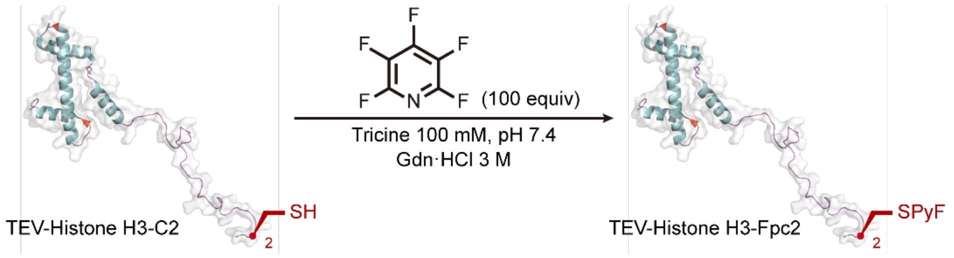

The Cys-tetrafluoropyridylsulfide-containing TEV-Histone H3-Fpc2 was prepared according to general procedure 1.

ESI-MS spectrum for the modified histone is shown below.

Calculated mass = 16186

Observed mass = 16187

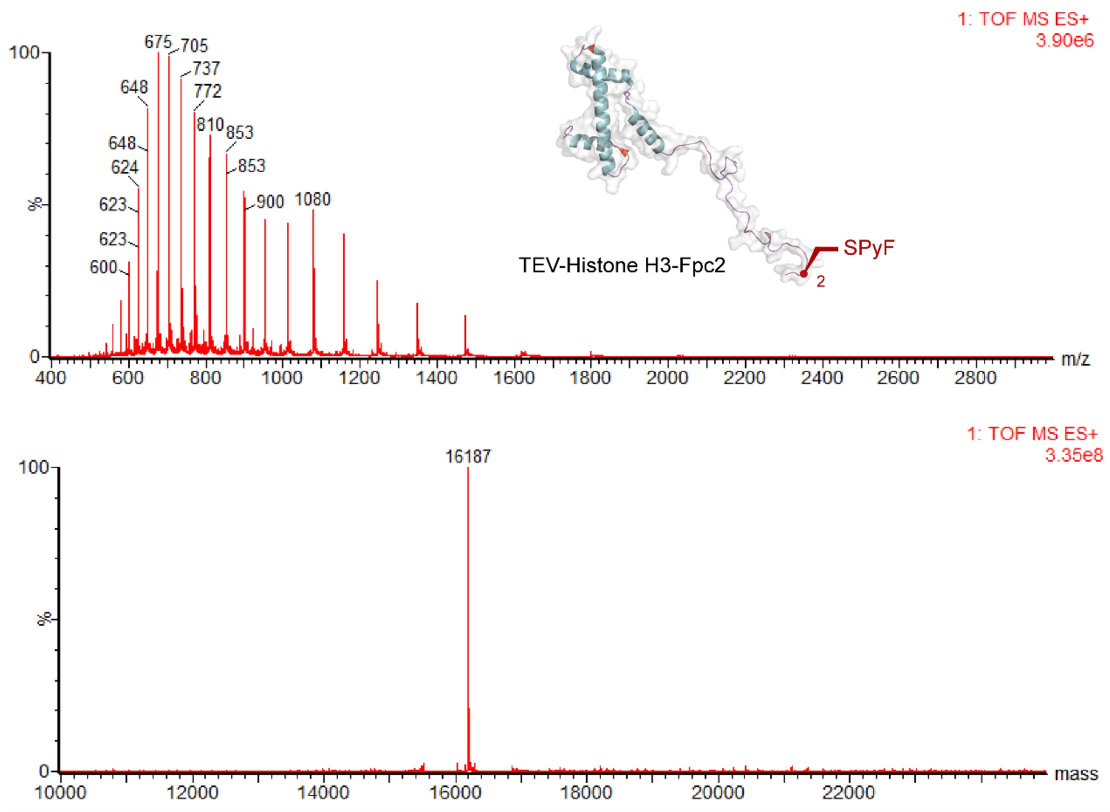

H4-Fpc20: protein expression, purification and Fpc generation

SGRGKGGKGLGKGGAKRHRCVLRDNIQGITKPAIRRLARRGGVKRISGLIYEETRGV LKVFLENVIRDAVTYTEHAKRKTVTAMDVVYALKRQGRTLYGFGG

Calculated mass = 11211;

Observed mass = 11212

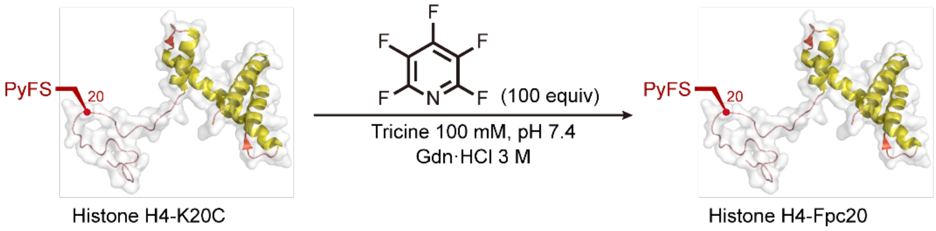

The Cys-tetrafluoropyridylsulfide-containing histone H4-Fpc20 was prepared according to general procedure 1.

ESI-MS spectrum for the modified histone is shown below.

Calculated mass = 11360;

Observed mass = 11360

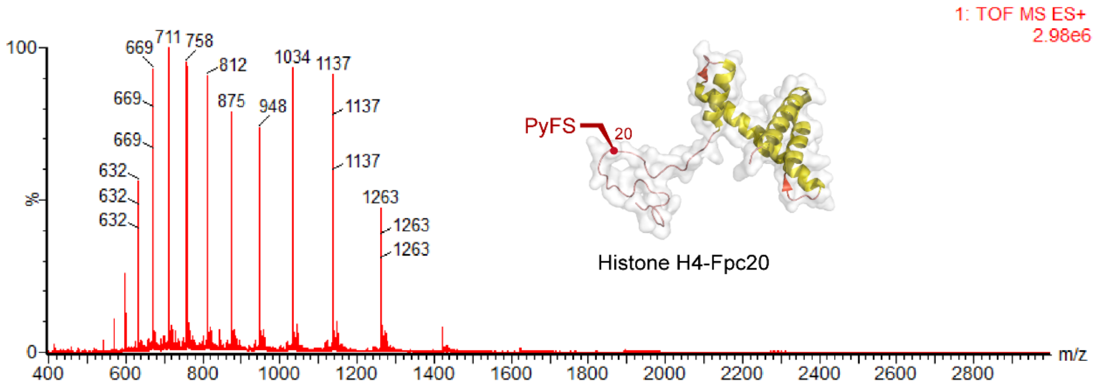

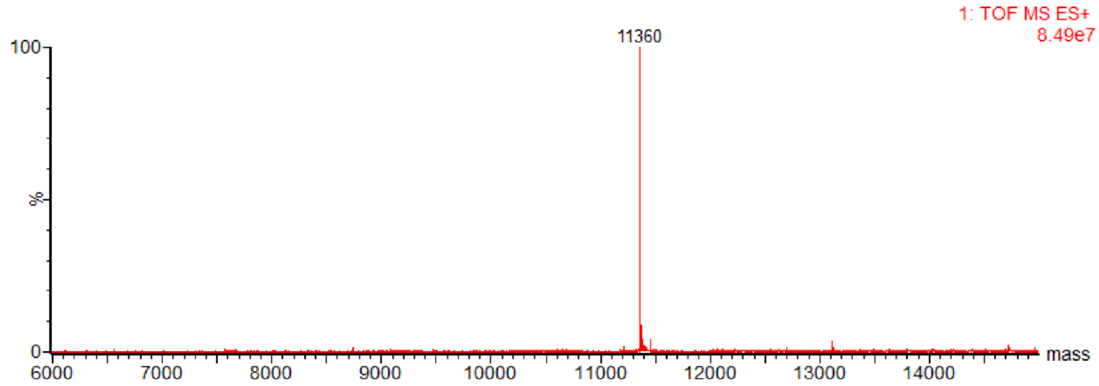

PstS -Fpc57, -Fpc103, -Fpc178, -Fpc197: protein expression, purification and Fpc generation

E. coli DH5α carrying the plasmid pET22b-PstS-A197C was kindly donated by Martin Webb and distributed by Addgene (Addgene plasmid #78198) as an agar stab. Site-directed mutagenesis to introduce Cys mutations was performed using QuikChange II (Agilent), according to manufacturer's instructions.

The appropriate PstS encoding plasmid was transformed into BL21 (DE3) cells, with ampicillin added. A single colony was selected after overnight growth and used to inoculate 20 mL LB medium with the same antibiotics. This culture was grown at 37 °C overnight. 20 mL of starter culture was then added to 1 L LB medium containing the same antibiotics and grown at 37 °C until OD_{600} = 0.6-0.8. IPTG was added, to a final concentration of 1 mM and the flask was shaken at 37 °C for 2-4 h. The cells were harvested by centrifugation (9,000 rpm, 20 min, 4 °C), suspended in 12.5 mL of lysis buffer each (10 mM Tris base, 1 mM DTT, pH 8.6, one tablet cOmplete™ Mini EDTA-free Protease Inhibitor Cocktail (Roche)) and frozen in liquid nitrogen, and stored at -80 °C.

Frozen cells were thawed in a water bath, when the solution was viscous DNase I was added, sonicated (10 cycles, 40% amplitude, 15 sec. on, 60 sec. off) and the cell debris was removed by centrifugation (30,000 rpm, 30 min, 4 °C). The solution was loaded onto a HiTrap Q HP column (5 mL) (GE Healthcare) and washed with 10 CV of binding buffer (10 mM Tris base, 1 mM DTT, pH 8.6) and eluted with a 20 CV gradient 0−100% elution buffer (10 mM Tris base, 200 mM NaCl, 1 mM DTT, pH 8.6). The fractions were analysed by SDS-PAGE and clean fractions were pooled together. The protein concentration was determined using an A280 spectrophotometer. The expression yield was determined to be 20 mg (30 mg/L expression volume). The protein solution was divided into aliquots, frozen in liquid nitrogen and stored at −80 °C until needed.

PstS-D57C

MEASLTGAGATFPAPVYAKWADTYQKETGNKVNYQGIGSSGGVKQIIANTVDFGAS
CAPLSDEKLAQEGLFQFPTVIGGVVLAVNIPGLKSGELVLDGKTLGDIYLGKIKKWD
DEAIAKLNPGLKLPSQNIAVVRRADGSGTSFVFTSYLAKVNEEWKNNVGTGSTVKW
PIGLGGKGNDGIAAFVQRLPGAIGYVEYAYAKQNNLAYTKLISADGKPVSPTEENFA
NAAKGADWSKTFAQDLTNQKGEDAWPITSTTFILIHKDQKKPEQGTEVLKFFDWAY
KTGAKQAN DLDYASLPDSVVEQVRAAWKTNIKDSSGKPLY

Calculated mass = 34541

Observed mass = 34541

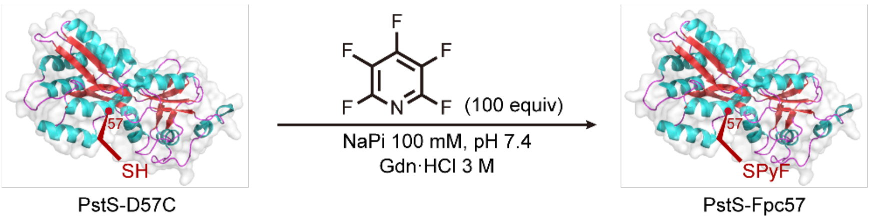

The Cys-tetrafluoropyridylsulfide-containing PstS-Fpc57 was prepared according to general procedure 1.

ESI-MS spectrum for the modified PstS is shown below.

Calculated mass = 34690

Observed mass = 34690

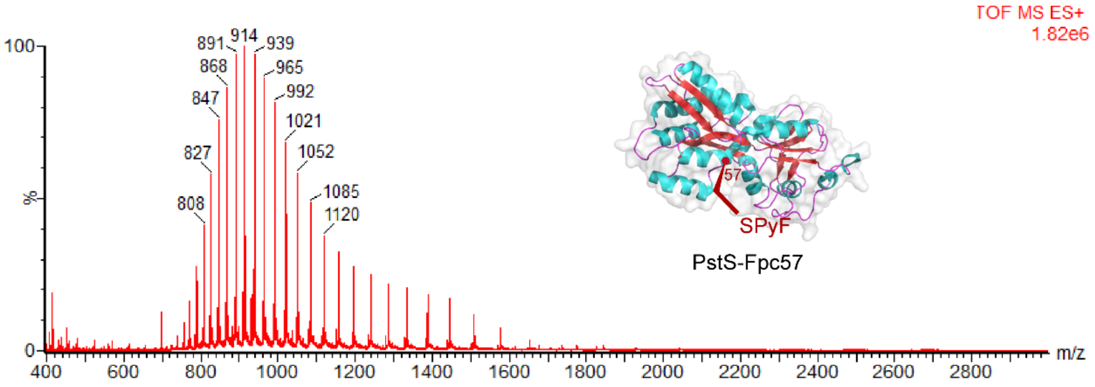

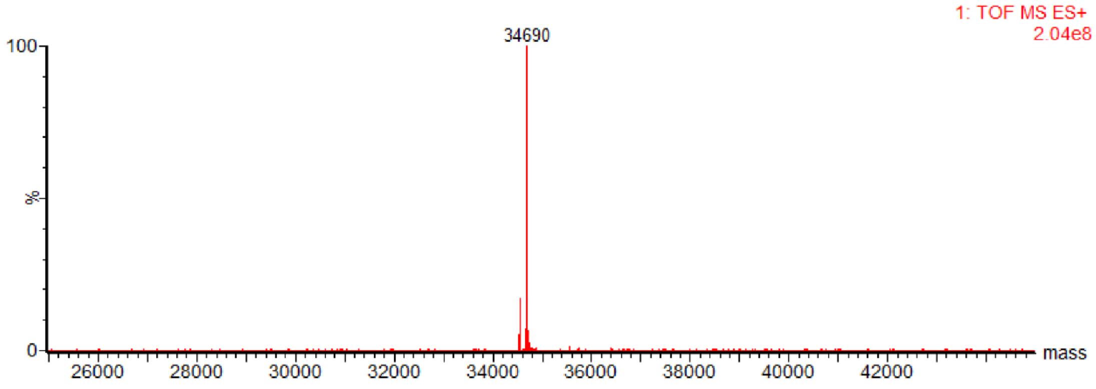

PstS-D103C

MEASLTGAGATFPAPVYAKWADTYQKETGNKVNYQGIGSSGGVKQIIANTVDFGAS

DAPLSDEKLAQEGLFQFPTVIGGVVLAVNIPGLKSGELVLDGKTLGCIYLGKIKKWD

DEAIAKLNPGLKLPSQNIAVVRRADGSGTSFVFTSYLAKVNEEWKNNVGTGSTVKW

PIGLGGKGNDGIAAFVQRLPGAIGYVEYAYAKQNNLAYTKLISADGKPVSPTEENFA

NAAKGADWSKTFAQDLTNQKGEDAWPITSTTFILIHKDQKKPEQGTEVLKFFDWAY

KTGAKQANDLDYASLPDSVVEQVRAAWKTNIKDSSGKPLY

Calculated mass = 34541
Observed mass = 34541

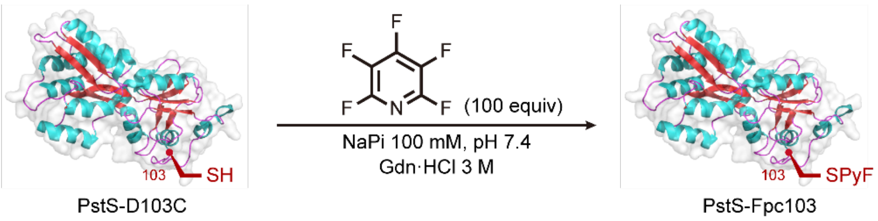

The Cys-tetrafluoropyridylsulfide-containing PstS-Fpc103 was prepared according to general procedure 1.

ESI-MS spectrum for the modified PstS is shown below.

Calculated mass = 34690

Observed mass = 34690

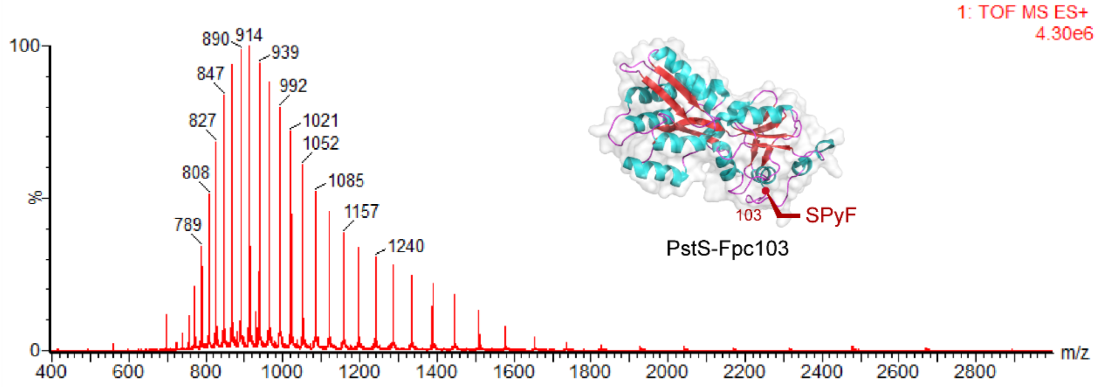

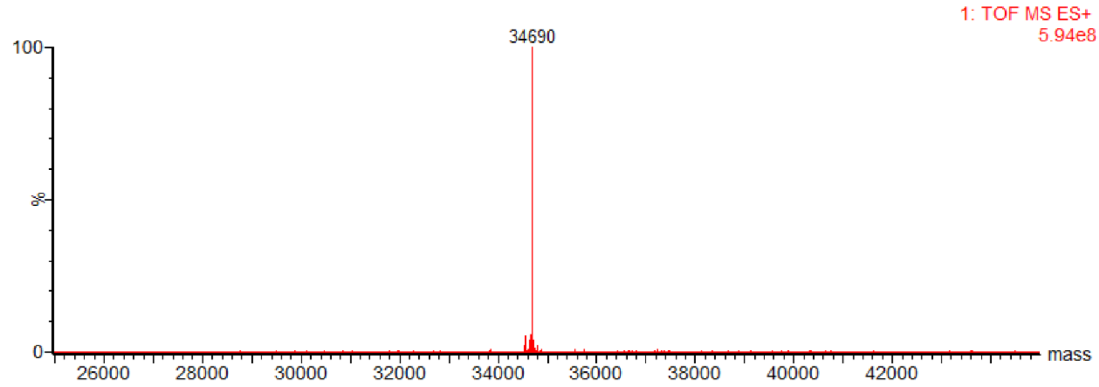

PstS-D178C

MEASLTGAGATFPAPVYAKWADTYQKETGNKVNYQGIGSSGGVKQIIANTVDFGAS
DAPLSDEKLAQEGLFQFPTVIGGVVLAVNIPGLKSGELVLDGKTLGDIYLGKIKKWD
DEAIAKLNPGLKLPSQNIAVVRRADGSGTSFVFTSYLAKVNEEWKNNVGTGSTVKW
PIGLGGKGNCGIAAFVQRLPGAIGYVEYAYAKQNNLAYTKLISADGKPVSPTEENFA
NAAKGADWSKTFAQDLTNQKGEDAWPITSTTFILIHKDQKKPEQGTEVLKFFDWAY
KTGAKQANDLDYASLPDSVVEQVRAAWKTNIKDSSGKPLY

Calculated mass = 34541

Observed mass = 34541

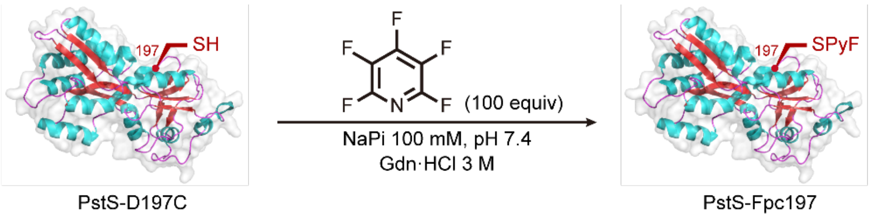

The Cys-tetrafluoropyridylsulfide-containing PstS_178PyfS was prepared according to general procedure 1.

ESI-MS spectrum for the modified PstS is shown below.

Calculated mass = 34690

Observed mass = 34690

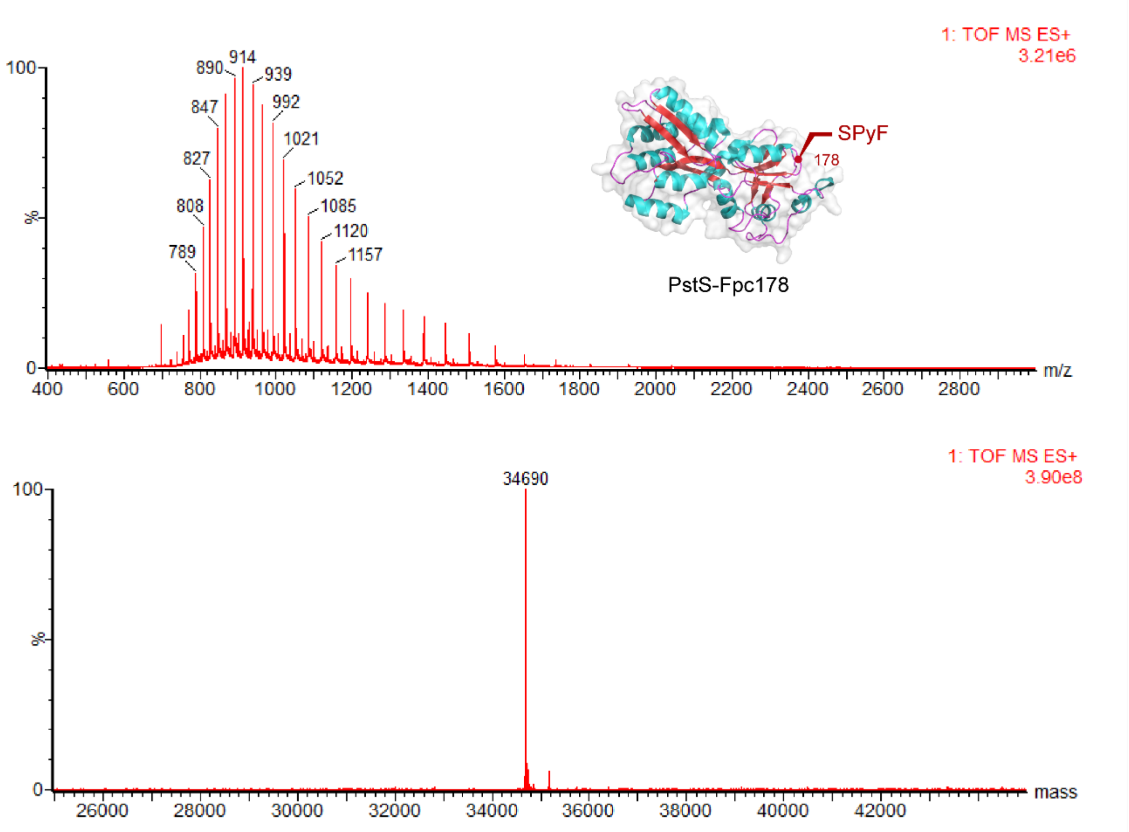

PstS-A197C

MEASLTGAGATFPAPVYAKWADTYQKETGNKVNYQGIGSSGGVKQIIANTVDFGAS
DAPLSDEKLAQEGLFQFPTVIGGVVLAVNIPGLKSGELVLDGKTLGDIYLGKIKKWD
DEAIAKLNPGLKLPSQNIAVVRRADGSGTSFVFTSYLAKVNEEWKNNVGTGSTVKW
PIGLGGKGNDGIAAFVQRLPGAIGYVEYCYAKQNNLAYTKLISADGKPVSPTEENFA
NAAKGADWSKTFAQDLTNQKGEDAWPITSTTFILIHKDQKKPEQGTEVLKFFDWAY
KTGAKQANDLDYASLPDSVVEQVRAAWKTNIKDSSGKPLY

Calculated mass = 34585

Observed mass = 34585

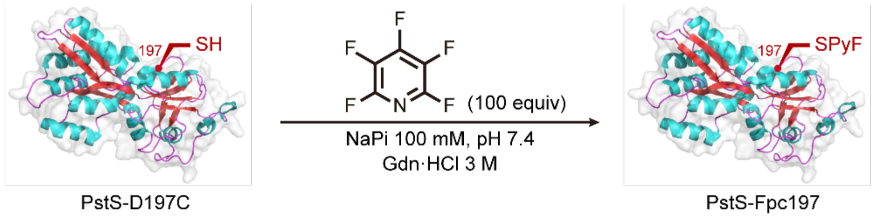

The Cys-tetrafluoropyridylsulfide-containing PstS-Fpc197 was prepared according to general procedure 1.

ESI-MS spectrum for the modified PstS is shown below.

Calculated mass = 34734

Observed mass = 34734

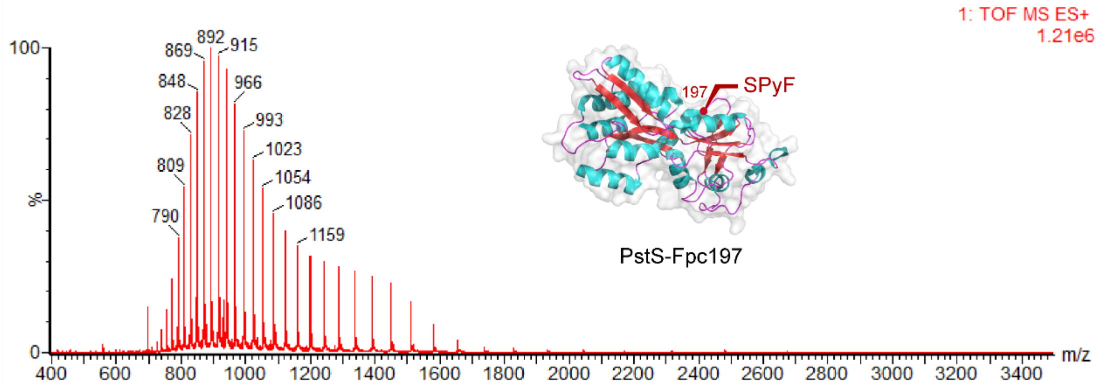

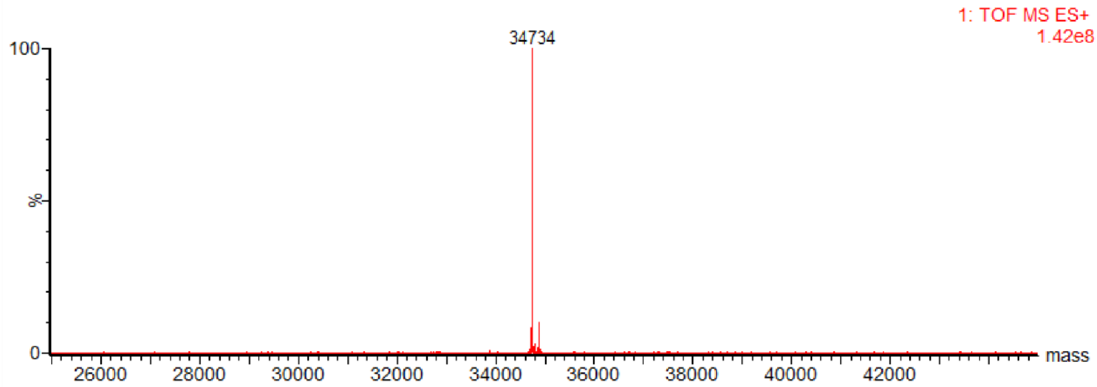

pre-SUMO1-Fpc51: protein expression, purification and Fpc generation

pre-SUMO1-Cys51 protein was expressed and purified following a previously published procedure.[15]

pre-SUMO1-Cys51

ADQEAKPSTEDLGDKKEGEYIKLKVIGQDSSEIHFKVKMTTHLKKLKESYCQRQGVPMNSLRFLFEGQRIADNHTPKELGMEEEDVIEVYQEQTGGHSTVLEHHHHHH

Calculated mass = 12475

Observed mass = 12474

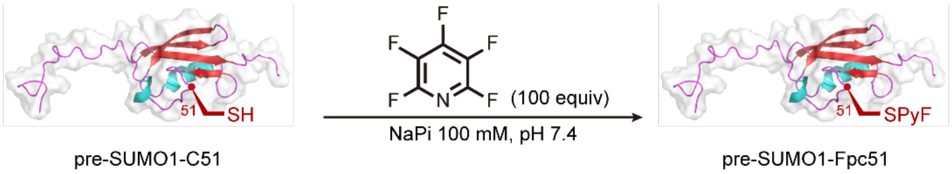

The Cys-tetrafluoropyridylsulfide-containing pre-SUMO1-Fpc51 was prepared according to general procedure 1.

ESI-MS spectrum for the modified pre-SUMO1 is shown below.

Calculated mass = 12623

Observed mass = 12623

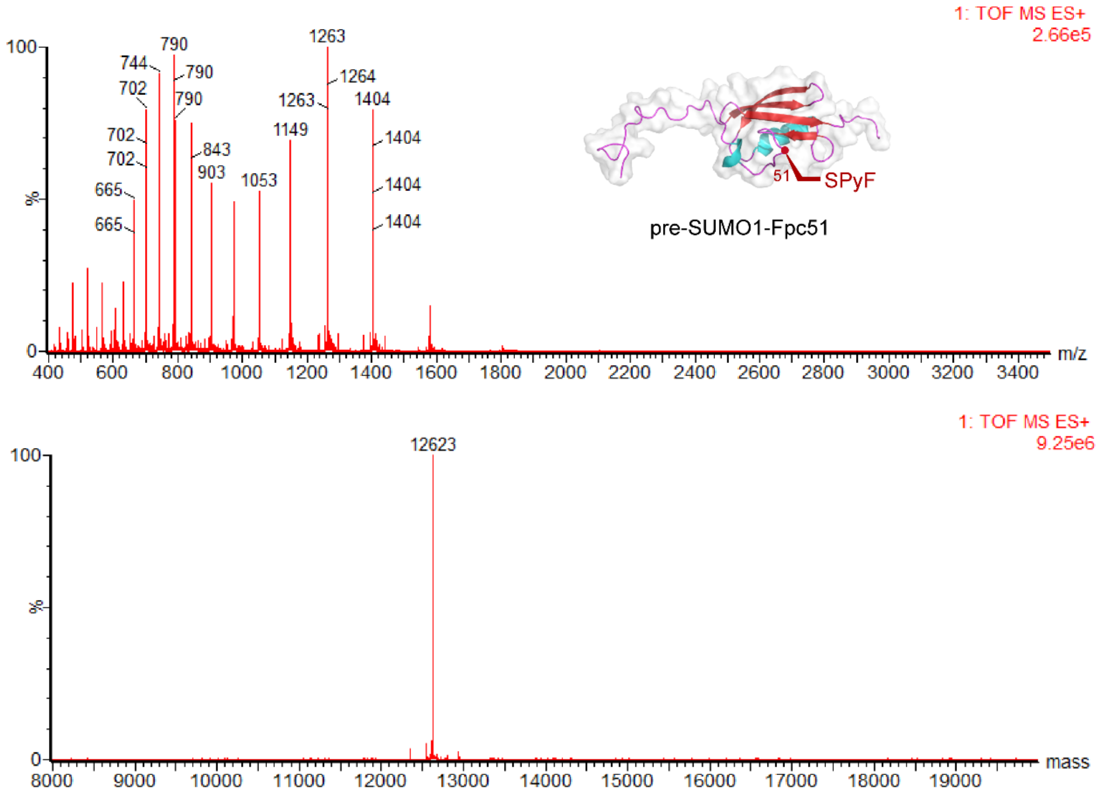

Npβ-Fpc61: protein expression, purification and Fpc generation

Npβ-C61 was expressed and purified following a previously published procedure.[15]

Npβ-Cys61

MFSSHHHHHHSSGLVPRGSHIDVGKLRQLYAAGERDFSIVDLRGAVLENINLSGAIL
HGACLDEANLQQANLSRADLSGATLNGADLRGANLSKADLSDAILDNAILEGAILDE
AVLNQANLKAANLEQAILSHANIREADLSEANLEAADLSGADLAIADLHQANLHQA
ALERANLTGANLEDANLEGT ILEGGNNNLAT

Calculated mass = 21031

Observed mass = 21031

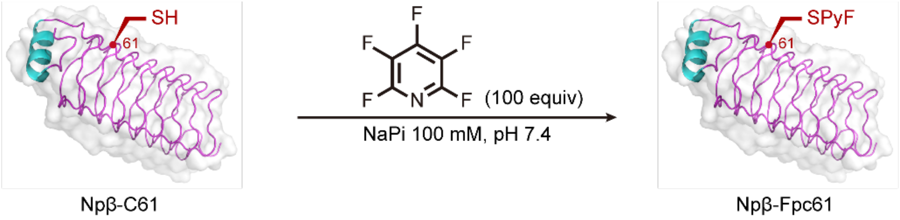

The Cys-tetrafluoropyridylsulfide-containing Npβ-Fpc61 was prepared according to general procedure 1.

ESI-MS spectrum for the modified Npβ is shown below.

Calculated mass = 21180

Observed mass = 21180

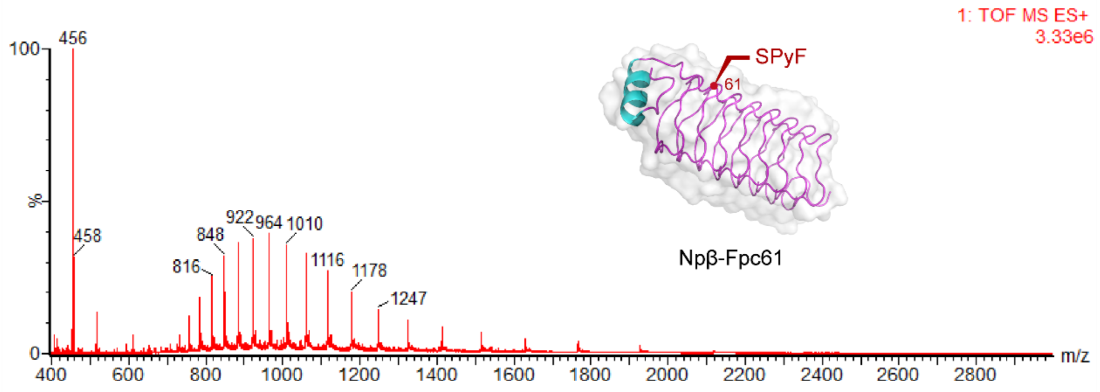

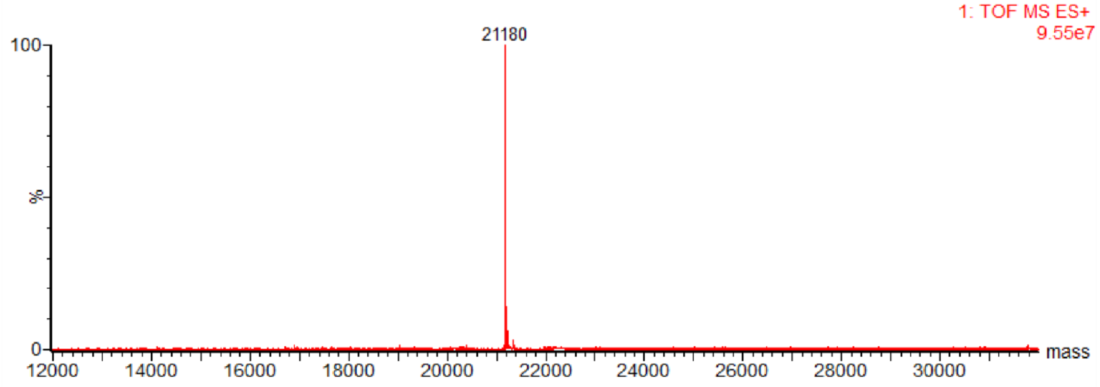

cAbVCAM1-Fpc118: protein expression, purification and Fpc generation

A fresh stock of WK6 competent *E. coli* cells (received as a gift from Prof. Ray Owens) was amplified according to the manufacturer's instructions

Single colonies were transferred to 15 mL of LB media supplemented with ampicillin (100 μg/mL) and incubated at 37 °C for 16 hours. The resulting suspension was immediately used to inoculate 1 L Terrific Broth (TB) media supplemented with ampicillin (100 μg/mL, 0.1 % glucose, and 2 mM $MgCl_2$. The cultures were incubated at 37 °C (180 rpm), for approximately 4 hours, until an OD_{600} between 0.9 and 1.1 was reached. Protein overexpression was induced by the addition of IPTG (final conc. 1 mM) and the cultures were incubated for 16 more hours at 27 °C (180 rpm). The cells were harvested by centrifugation (12,000 x g, 10 minutes, 4 °C) to afford cell pellets (25 g wet weight per liter of culture). The cell pellets were kept at -80 °C until further manipulation.

A single cell pellet was thawed on ice and mixed with 40 mL of TES lysis buffer (0.2 M Tris pH 7.8, 0.5 mM EDTA, 0.5 M sucrose) containing one pre-dissolved cOmplete protease inhibitor mix tablet (EDTA free, Roche). The cell pellet was vortexed until bacterial clumps were not visible and then shaken via end-over-end mixing for 30 minutes at 4 °C. 2 mg of DNase I were added, and the mixture was further shaken via end-over-end mixing for 2 hours at 4 °C. The lysate was centrifuged at 4 °C and 22,000× g for 15 min.

Supernatant was filtered through 0.2 μm syringe filter and loaded to a pre-equilibrated HisTrap HP 5 mL column (GE Healthcare, 2.5 mL/min), using a 50 mL superloop (GE Healthcare). The protein was eluted running a stepwise gradient of 30 CV to 100% buffer B (10 CV Buffer A, 2 CV 5% Buffer B, 2 CV 7% Buffer B, 2 CV 10% Buffer B, 2 CV 20% Buffer B, 2 CV 35% Buffer B, 8 CV 100% Buffer B). The fractions were analysed by SDS-PAGE and clean fractions containing protein were combined. The protein fractions were buffer exchanged to 50 mM NaPi, pH 8 using an Amicon® Ultra-15 Centrifugal Filter Unit and samples were flash-frozen in liquid nitrogen and stored at -80 °C. Protein expression yield was measured after the final buffer exchange and was measured at 11.2 mg/L.

Buffer A - 20 mM Tris-HCl, 15 mM imidazole, 500 mM NaCl, 1 mM DTT, 0.05% (v/v) β-mercaptoethanol, pH 7.8

Buffer B - 20 mM Tris-HCl, 500 mM imidazole, 500 mM NaCl, 1 mM DTT, 0.05% (v/v) β-mercaptoethanol, pH 7.8

cAbVCAM-Cys118

NVQLQESGGGSVQTGGSLRLSCAASGYTNSIMYMAWFRQAPGKKREGVAAIRFPDD SAYYAGSVKGRFTISHDNAKNTVYLQMNNLNPEDTAMYYCAARSSPYSFAWNDPN YNYWGCGTQVTVSSHHHHHH

Calculated mass = 14619

Observed mass = 14619

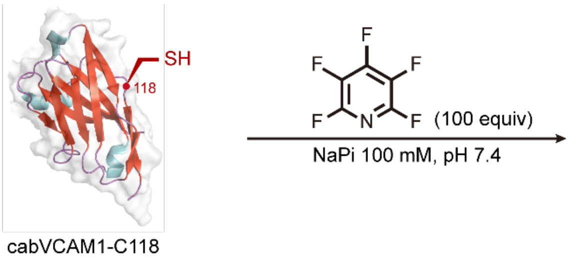

The Cys-tetrafluoropyridylsulfide-containing cabVCAM-Fpc118 was prepared according to general procedure 1.

ESI-MS spectrum for the modified cAbVCAM is shown below.

Calculated mass = 14768

Observed mass = 14767

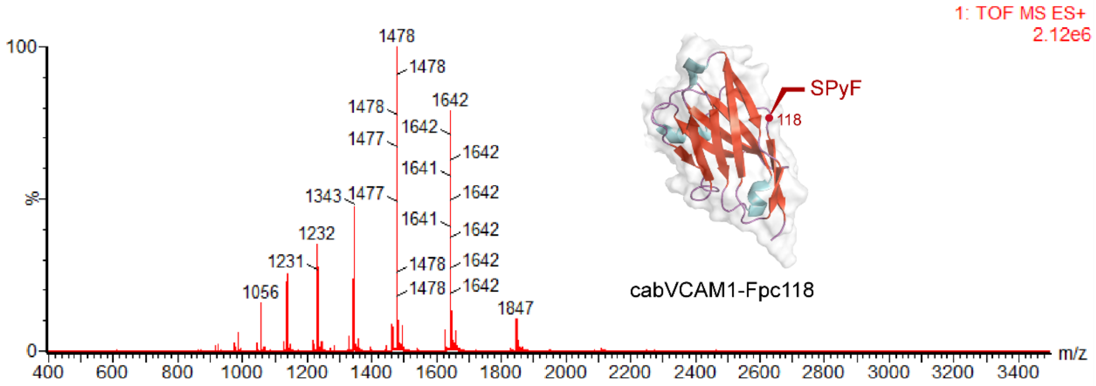

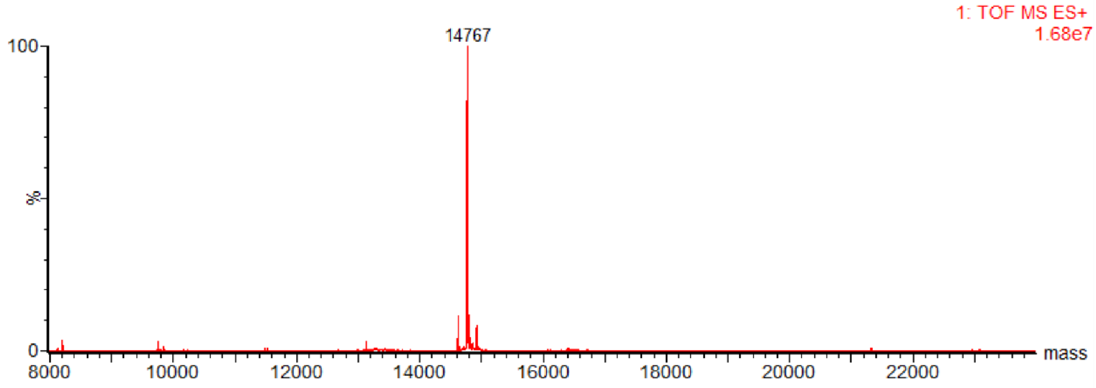

AcrA-Fpc123: protein expression, purification and Fpc generation

AcrA-C123 was expressed and purified following a previously published procedure.[15]

AcrA-Cys123

SKEEAPKIQMPPQPVTTMSAKSEDLPLSFTYPAKLVSDYDVIIKPQVSGVIVNKLFKA
GDKVKKGQTLFIIEQDKFKASVDSAYGQALMAKATFENASKDFCRSKALFSKSAISQ
KEYDSSLATFNNSKASLASARAQLANARIDLDHTEIKAPFDGTIGDALVNIGDYVSAS
TTELVRVTNLNPIYADFFISDTDKLNLVRNTQSGKWDLDSIHANLNLNGETVQGKLY
FIDSVIDANSGTVKAKAVFDNNNSTLLPGAFATITSEGFIQKNGFKVPQIGVKQDQND
VYVLLVKNGKVEKSSVHISYQNNEYAIIDKGLQNGDKIILDNFKKIQVGSEVKEIGAQ
LEHHHHHH

Calculated mass = 38817

Observed mass = 38817

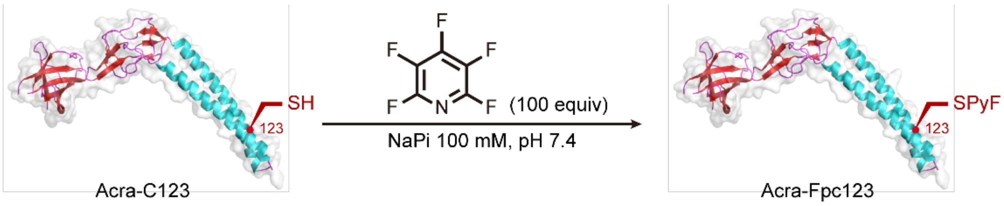

The Cys-tetrafluoropyridylsulfide-containing Acra-Fpc123 was prepared according to general procedure 1.

ESI-MS spectrum for the modified Acra is shown below.

Calculated mass = 38966

Observed mass = 38966

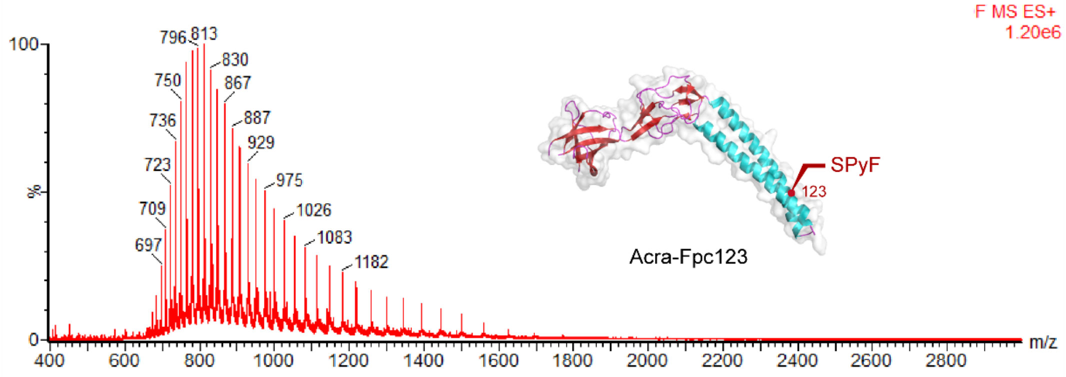

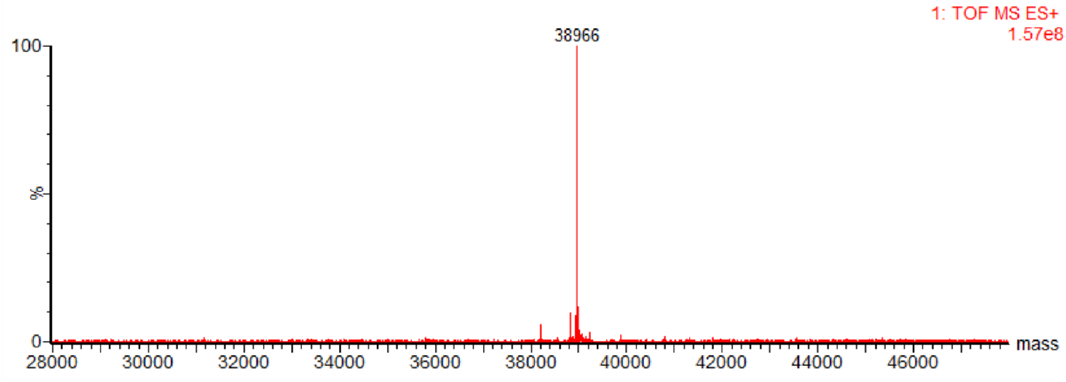

On-protein radical trapping from PstS-Fpc178

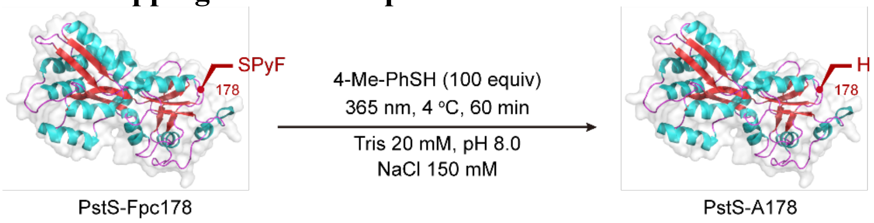

The PstS-A178 was prepared according to general procedure 2.

ESI-MS spectrum for the modified PstS is shown below.

Calculated mass = 34509

Observed mass = 34509

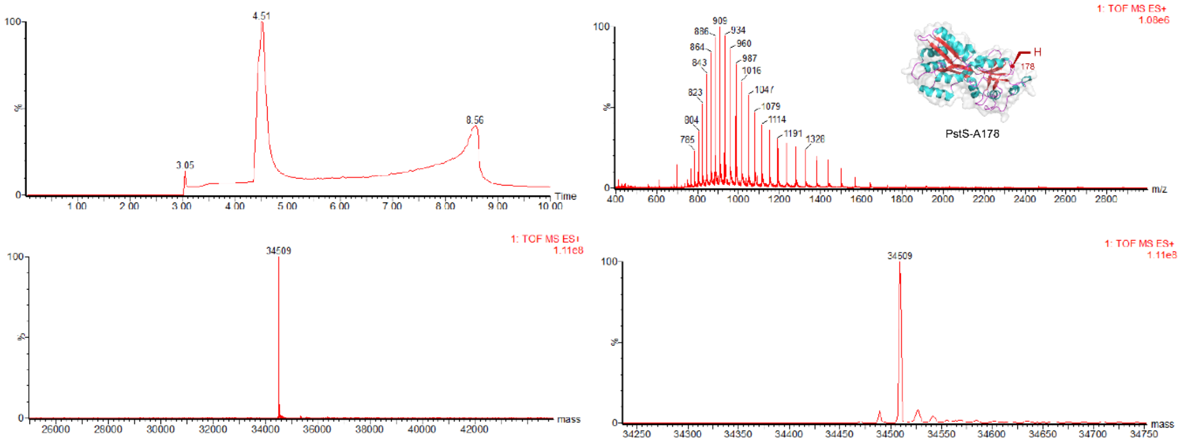

Conversion

PstS-Fpc178	PstS-Ala178	
0%	>98% conversion	

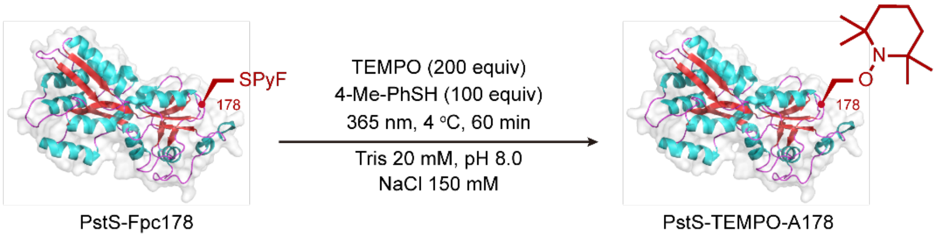

The PstS-TEMPO-A178 was prepared according to general procedure 3.

ESI-MS spectrum for the modified PstS is shown below.

Calculated mass = 34664

Observed mass = 34663

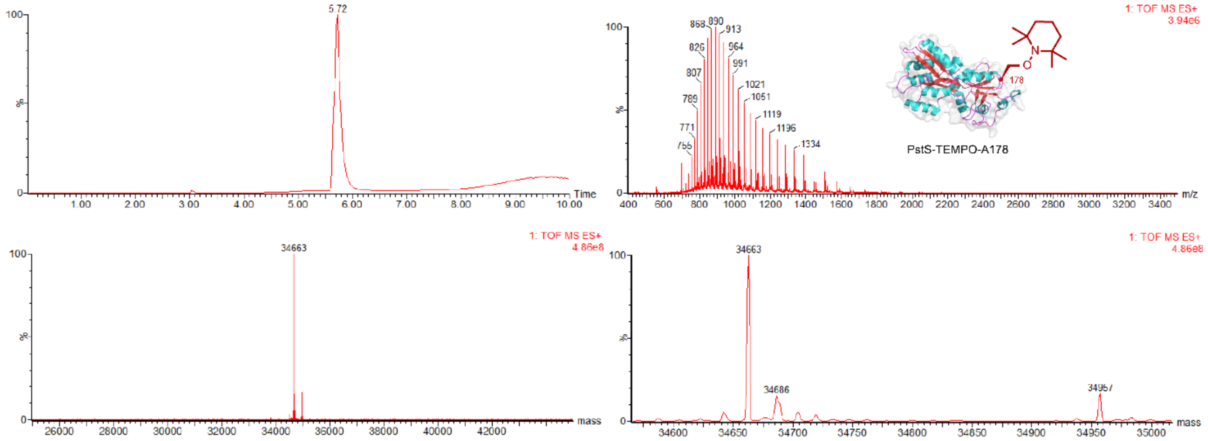

Conversion

PstS-Fpc178	PstS-Ala178	PstS-TEMPO-A178
10%	0%	90%

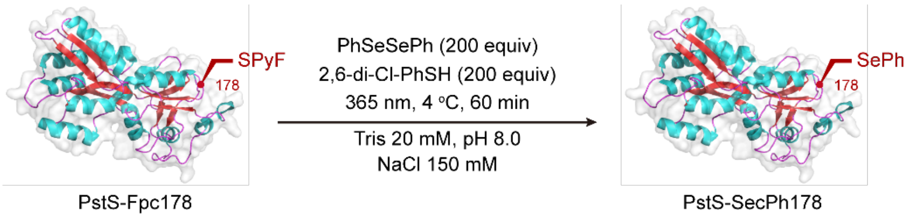

The PstS-SecPh178 was prepared according to general procedure 4.

ESI-MS spectrum for the modified PstS is shown below.

Calculated mass = 34664

Observed mass = 34664

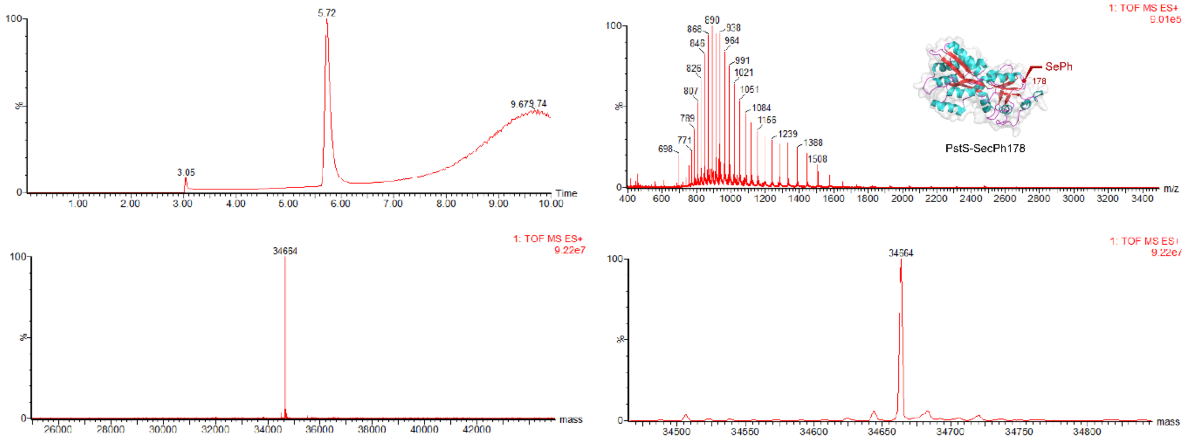

Conversion

PstS-Fpc178	PstS-Ala178	PstS-SecPh178
0%	0%	>98% conversion

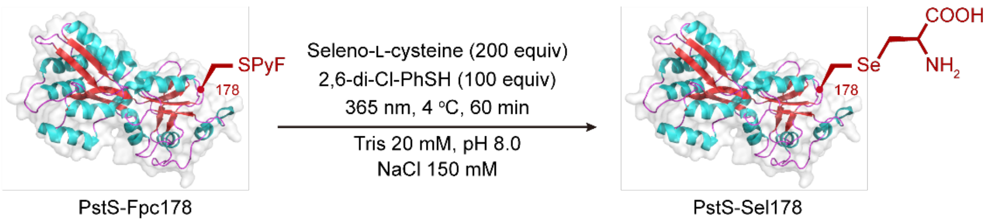

The PstS-Sel178 was prepared according to general procedure 4.

ESI-MS spectrum for the modified PstS is shown below.

Calculated mass = 34675

Observed mass = 34675

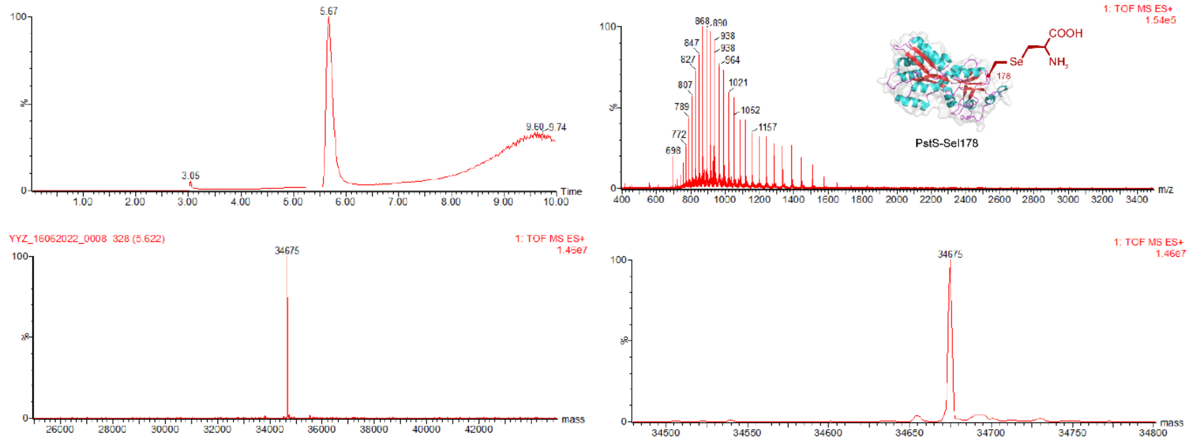

Conversion

PstS-Fpc178	PstS-Ala178	PstS-SecPh178
0%	0%	>98% conversion

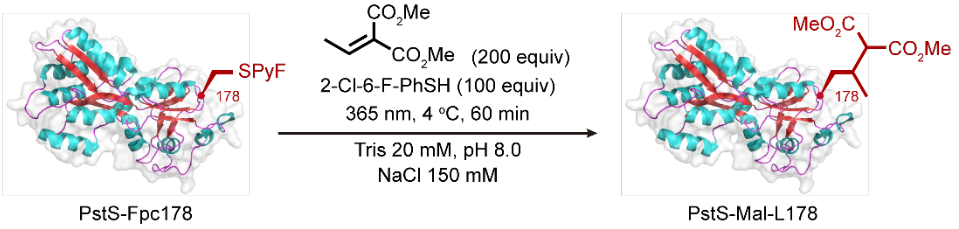

The PstS-Mal-L178 was prepared according to general procedure 5.

ESI-MS spectrum for the modified PstS is shown below.

Calculated mass = 34667

Observed mass = 34668

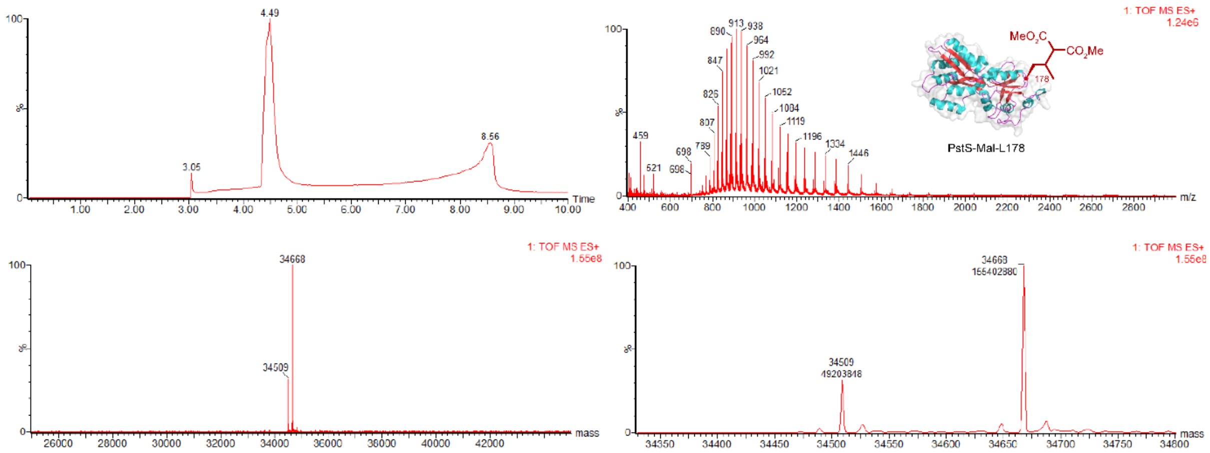

Conversion

PstS-Fpc178	PstS-Ala178	PstS-Mal-L178
0%	25%	75%

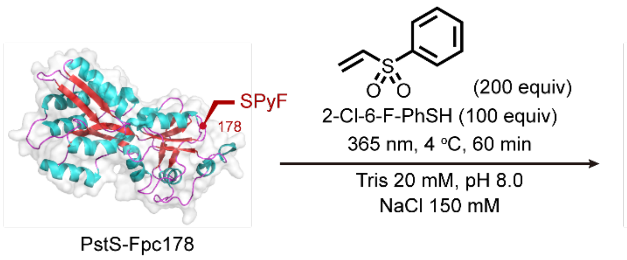

The PstS-A(Sulfone)178 was prepared according to general procedure 5.

ESI-MS spectrum for the modified PstS is shown below.

Calculated mass = 34676 (1 adduct), 34844 (2 adducts), 35012 (3 adducts)

Observed mass = 34676 (1 adduct), 34844 (2 adducts), 35013 (3 adducts)

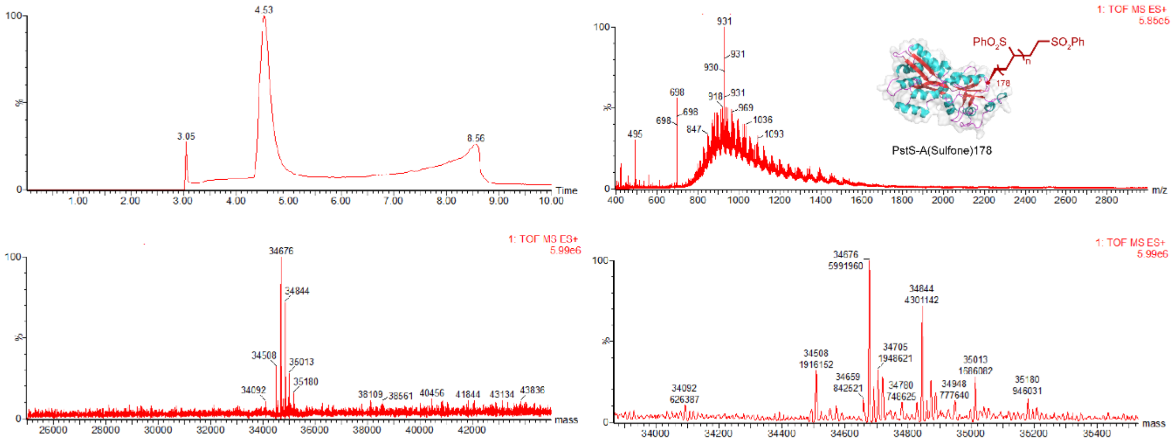

Conversion

PstS-Fpc178	PstS-Ala178	PstS-A(Sulfone)178 (1-4 adducts)
0%	13%	87%

See also **Supplementary Figure 15**, which suggests that adducts may be non-specific.

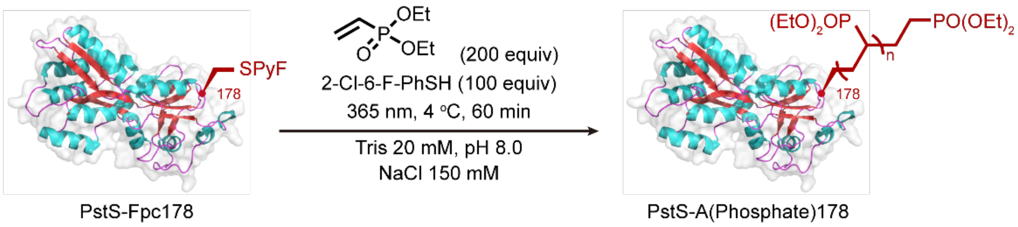

The PstS-A(Phosphonate)178 was prepared according to general procedure 5.

ESI-MS spectrum for the modified PstS is shown below.

Calculated mass = 34673 (n =0), 34837 (n = 1), 35001 (n = 2), 35165 (n = 3)

Observed mass = 34672 (n =0), 34837 (n = 1), 35000 (n = 2), 35164 (n = 3)

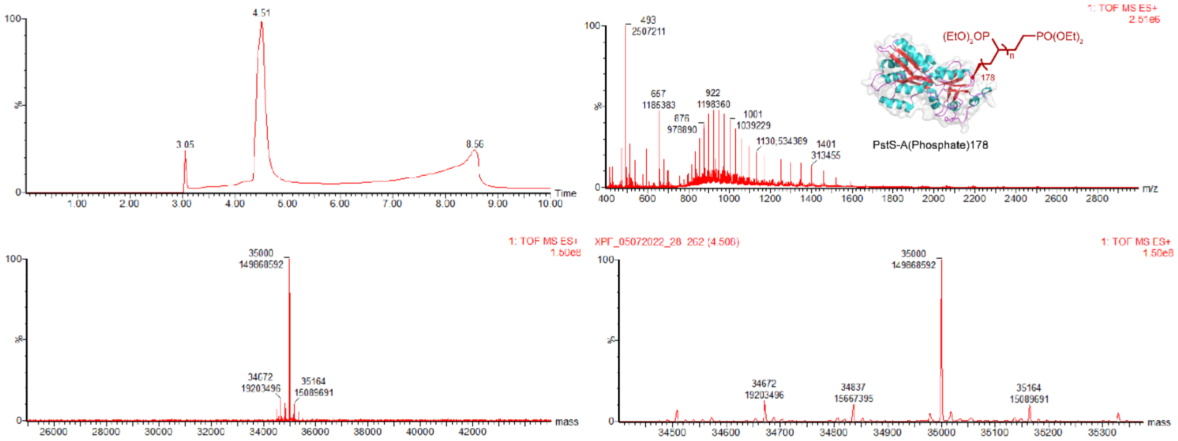

Conversion

PstS-Fpc178	PstS-Ala178	PstS-A(Phosphonate)178 (n = 0-3)
0%	0%	>98% conversion

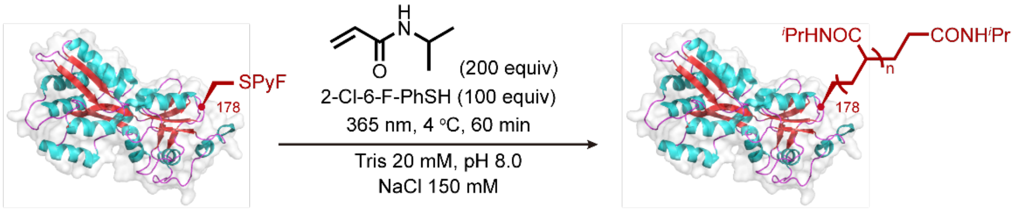

PstS-Fpc178 PstS-A(Amide)178

The PstS-A(Amide)178 was prepared according to general procedure 5.

ESI-MS spectrum for the modified PstS is shown below.

Calculated mass = 34847 (n = 2), 34960 (n = 3), 35073 (n = 4), 35186 (n = 5), 35299 (n = 6), 35412 (n = 7), 35525 (n = 8), 35638 (n = 9), 35751 (n = 10), 35864 (n = 11), 35977 (n = 12).

Observed mass = 34847 (n = 2), 34961 (n = 3), 35073 (n = 4), 35187 (n = 5), 35300 (n = 6), 35414 (n = 7), 35527 (n = 8), 35640 (n = 9), 35753 (n = 10), 35866 (n = 11), 35978 (n = 12).

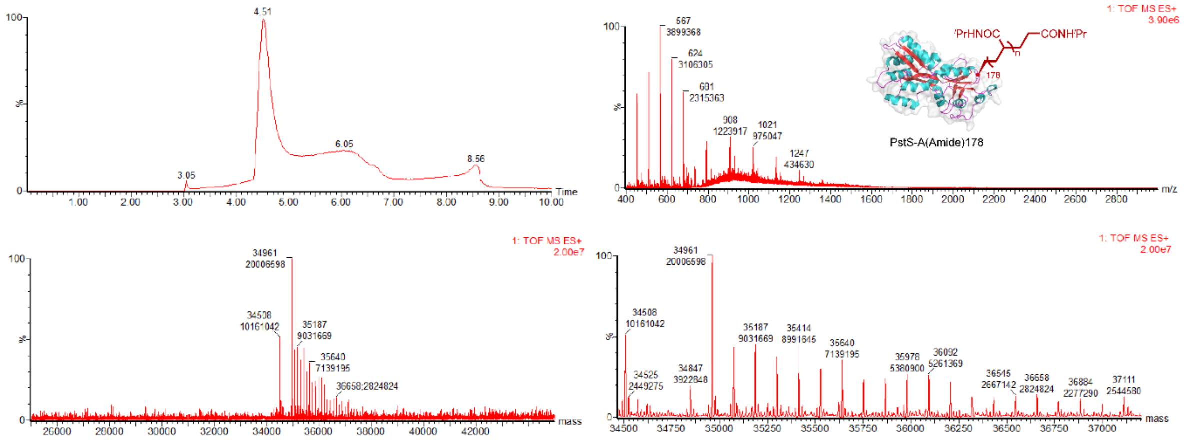

Conversion

PstS-Fpc178	PstS-Ala178	PstS-A(Amide)178 (n = 2-19)
0%	9%	91%

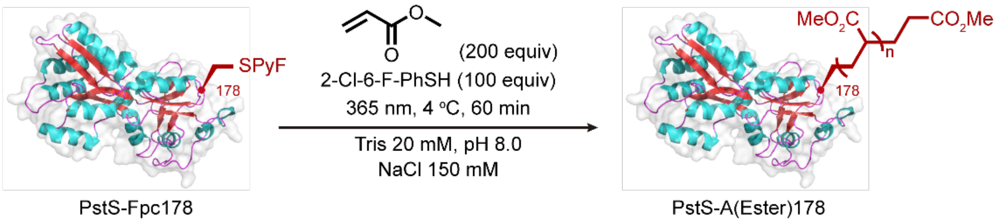

The PstS-A(Ester)178 was prepared according to general procedure 5.

ESI-MS spectrum for the modified PstS is shown below.

Calculated mass = 34766 (n = 2), 34852 (n = 3), 34938 (n = 4), 35024 (n = 5), 35110 (n = 6), 35196 (n = 7).

Observed mass = 34766 (n = 2), 34852 (n = 3), 35938 (n = 4), 35025 (n = 5), 35110 (n = 6), 35196 (n = 7).

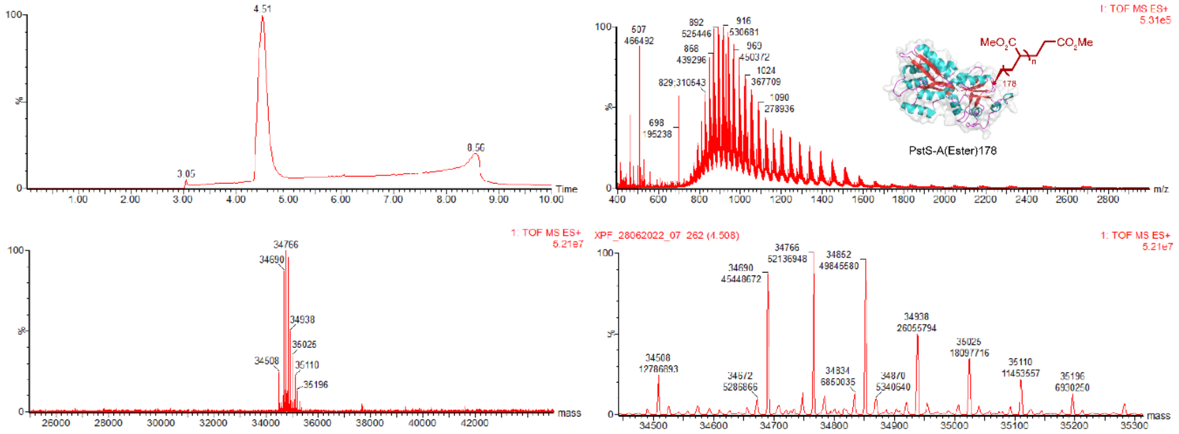

Conversion

PstS-Fpc178	PstS-Ala178	PstS-A(Ester)178 (n = 2-7)
20%	6%	74%

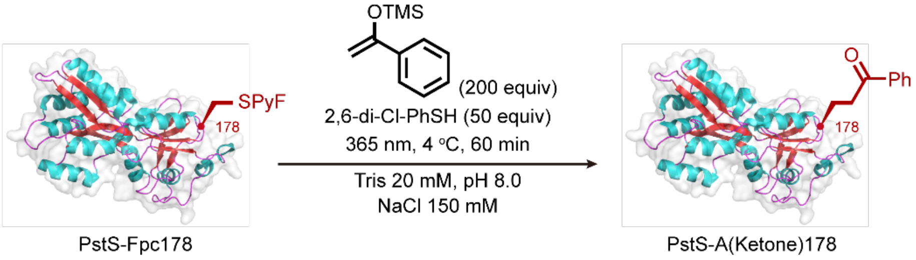

The PstS-A(Ketone)178 was prepared according to general procedure 5.

ESI-MS spectrum for the modified PstS is show below.

Calculated mass = 34627

Observed mass = 34628

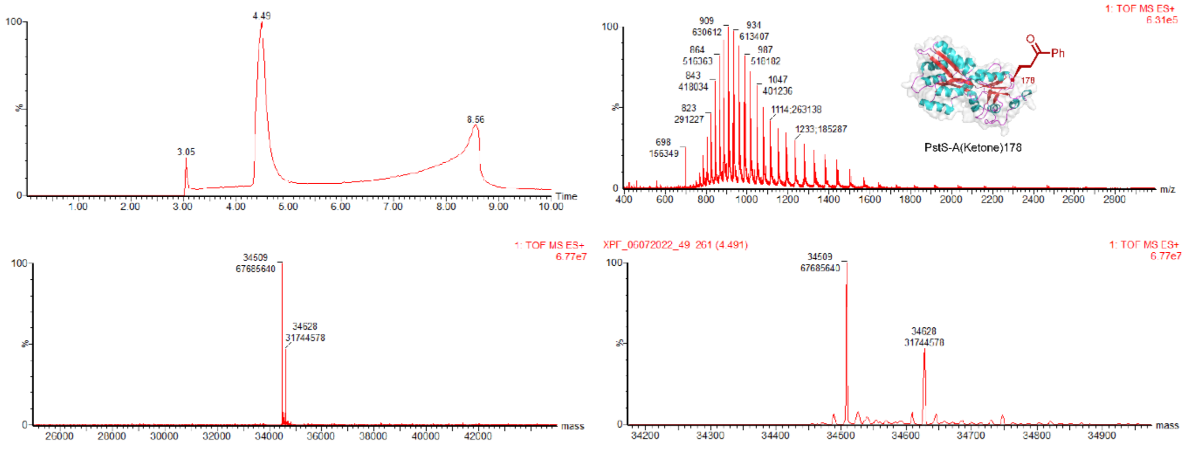

Conversion

PstS-Fpc178	PstS-Ala178	PstS-A(Ketone)178
0%	67%	33%

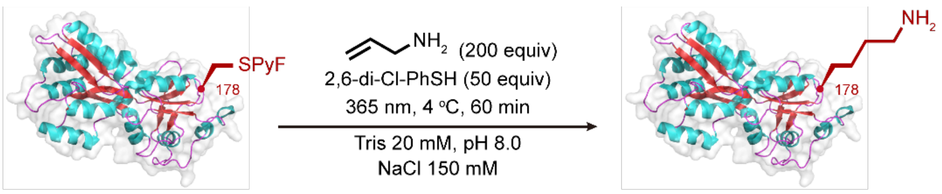

PstS-Fpc178 PstS-K178

The PstS-Lys178 was prepared according to general procedure 5.

ESI-MS spectrum for the modified PstS is shown below.

Calculated mass = 34566

Observed mass = 34565

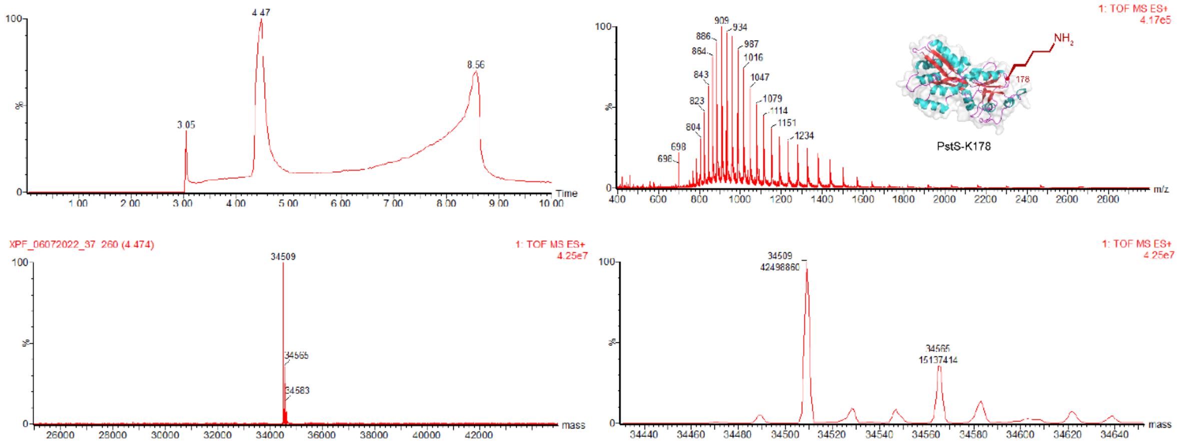

Conversion

PstS-Fpc178	PstS-Ala178	PstS-Lys178
0%	74%	26%

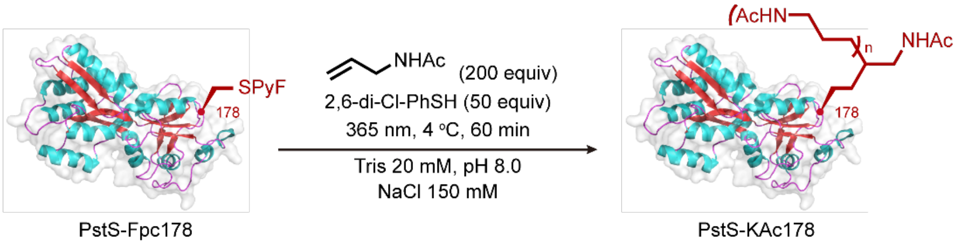

The PstS-KAc178 was prepared according to general procedure 5.

ESI-MS spectrum for the modified PstS is shown below.

Calculated mass = 34607 (n = 0), 34706 (n = 1)

Observed mass = 34607 (n = 0), 34706 (n = 1)

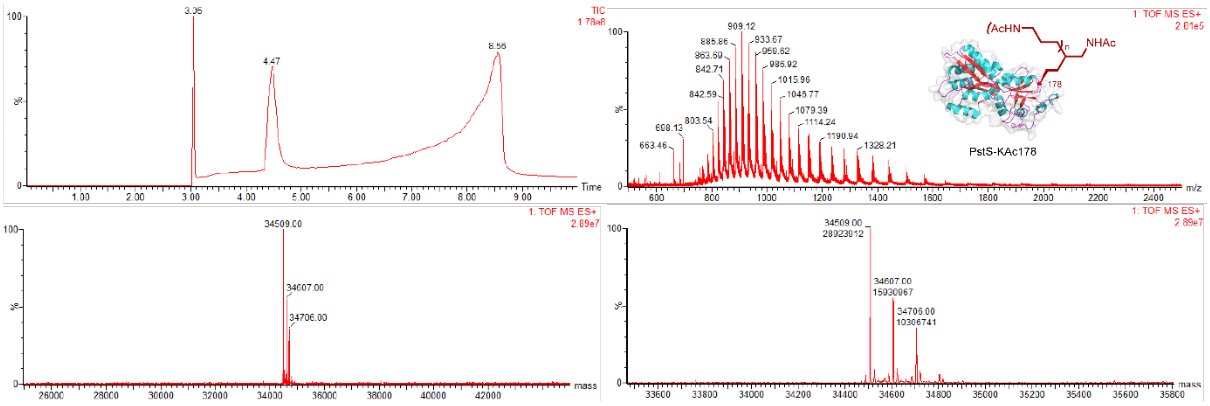

Conversion

PstS-Fpc178	PstS-Ala178	PstS-KAc178 (n = 0-1)
0%	52%	48%

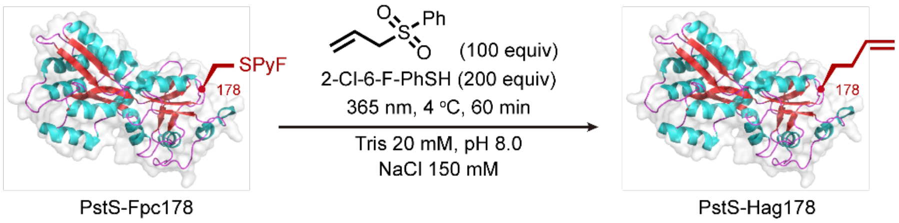

The PstS-Hag178 was prepared according to general procedure 5.

ESI-MS spectrum for the modified PstS is shown below.

Calculated mass = 34548

Observed mass = 34549

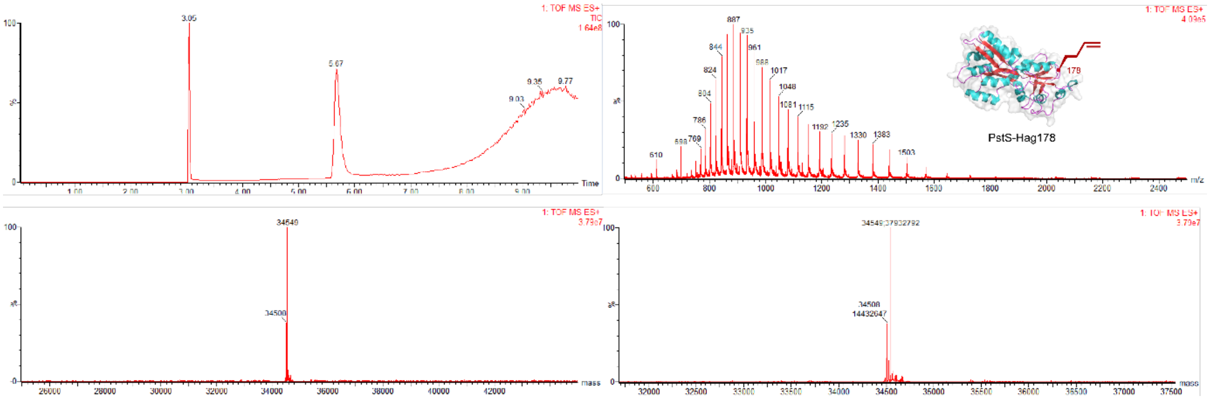

Conversion

PstS-Fpc178	PstS-Ala178	PstS-Hag178
0%	28%	72%

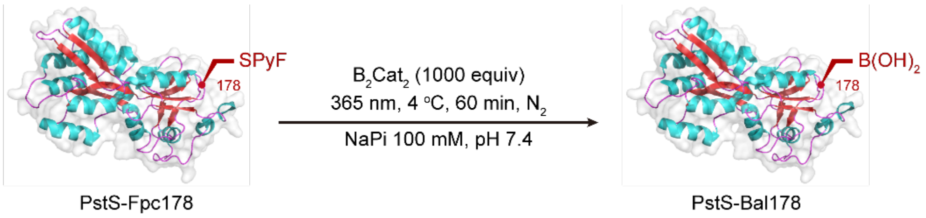

The PstS-Bal178 was prepared according to general procedure 6.

ESI-MS spectrum for the modified PstS is shown below.

Calculated mass = 34553 (Bal), 34535 (Bal-H_2O)

Observed mass = 34552 (Bal), 34533 (Bal-H_2O)

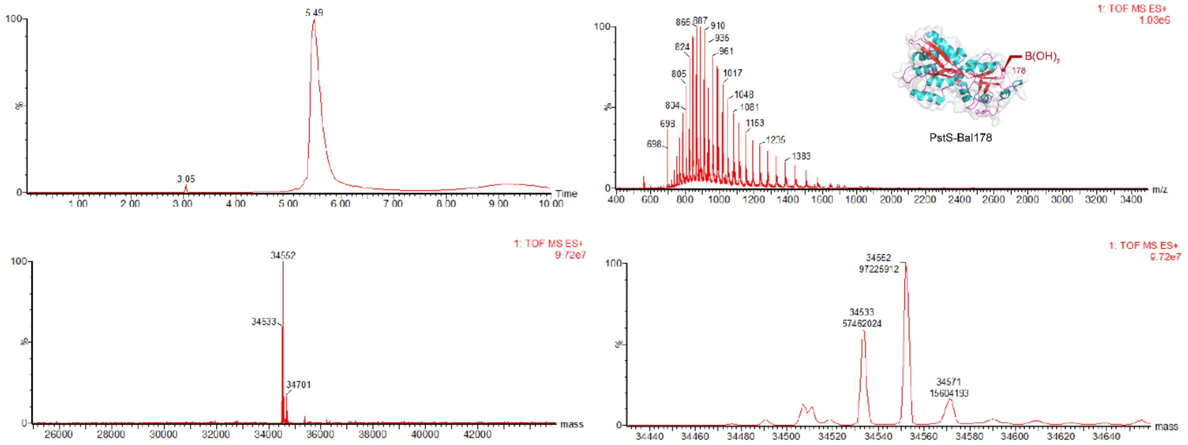

Conversion

PstS-Fpc178	PstS-Ala178	PstS-Bal178	
0%	9%	Bal	Bal-H_2O
		57%	34

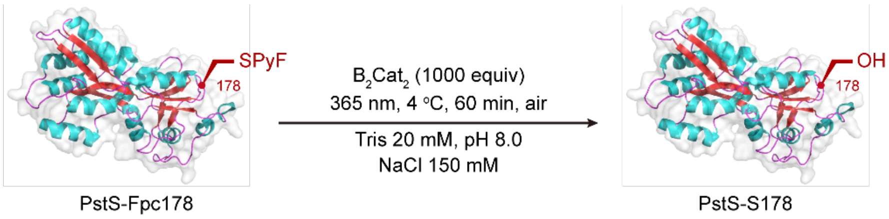

The PstS-Ser178 was prepared according to general procedure 7.

ESI-MS spectrum for the modified PstS is shown below.

Calculated mass = 34525

Observed mass = 34524

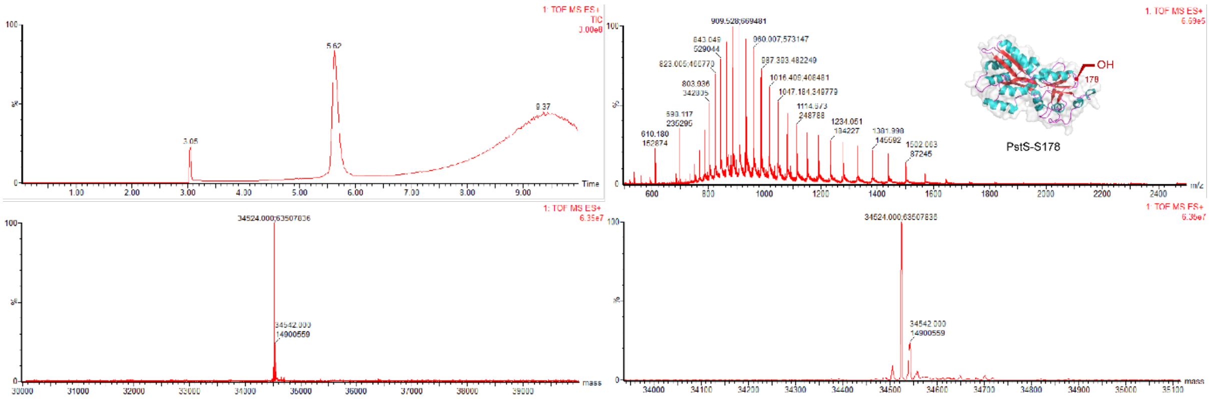

Conversion

PstS-Fpc178	PstS-Ala178	PstS-Ser178
0%	0%	81%

Widescale introduction of L-boronoalanine (L-Bal) into Proteins.

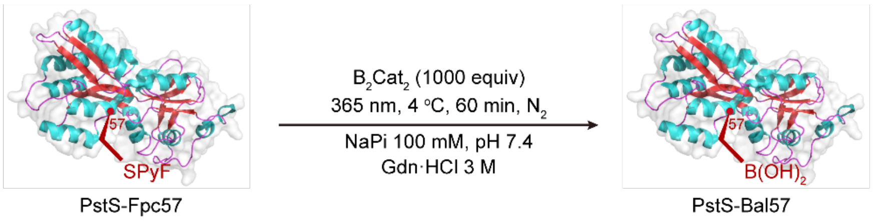

The PstS-Bal57 was prepared according to general procedure 8.

ESI-MS spectrum for the modified PstS is shown below.

Calculated mass = 34553 (Bal), 34535 (Bal-H_2O), 34517 (Bal-2H_2O)

Observed mass = 34551 (Bal), 34533 (Bal-H_2O), 34516 (Bal-2H_2O)

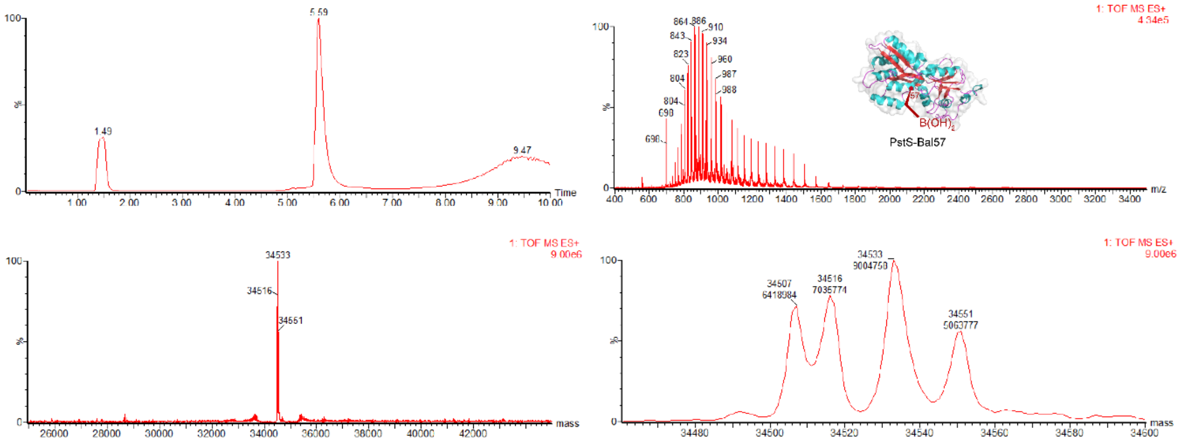

Conversion

PstS-Fpc57	PstS-A57	PstS-Bal57		
		Bal	Bal-H_2O	Bal-2H_2O
0%	23%	25%	32%	20%

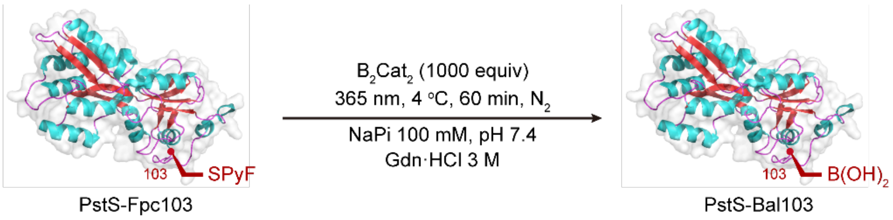

The PstS-Bal103 was prepared according to general procedure 8.

ESI-MS spectrum for the modified PstS is shown below.

Calculated mass = 34553 (Bal), 34535 (Bal-H_2O)

Observed mass = 34551 (Bal), 34536 (Bal-H_2O).

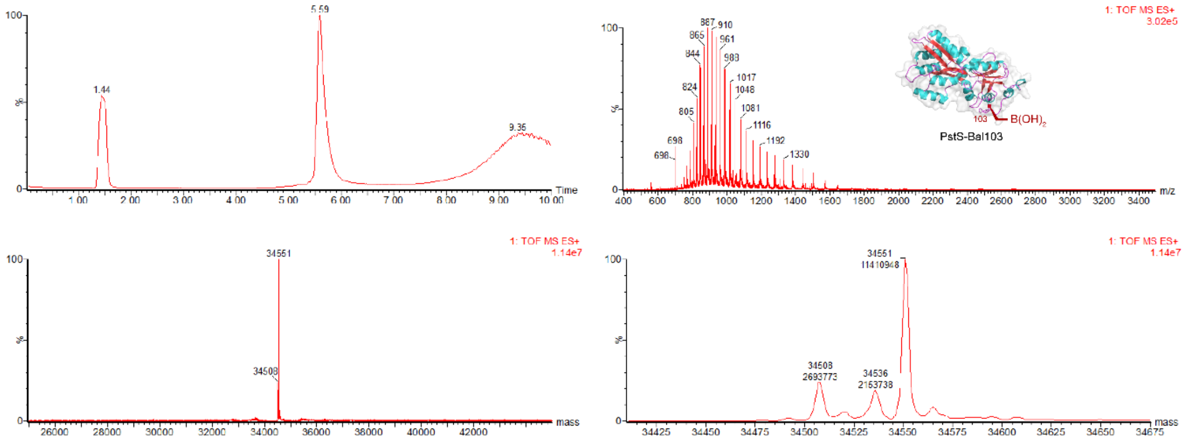

Conversion

PstS-Fpc103	PstS-A103	PstS-Bal103		
		Bal	Bal-H_2O	Bal-2H_2O
0%	17%	70%	13%	0%

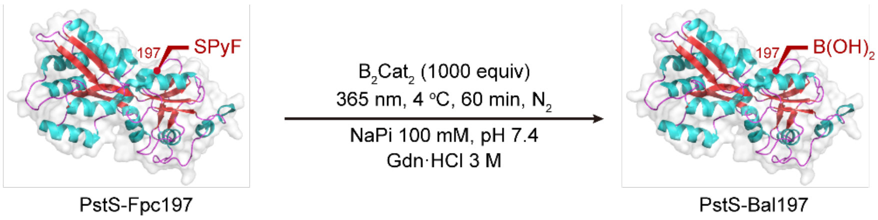

The PstS-Bal197 was prepared according to general procedure 8.

ESI-MS spectrum for the modified PstS is shown below.

Calculated mass = 34597 (Bal), 34579 (Bal-H_2O), 34561 (Bal-$2H_2O$)

Observed mass = 34596 (Bal), 34578 (Bal-H_2O), 34562 (Bal-$2H_2O$)

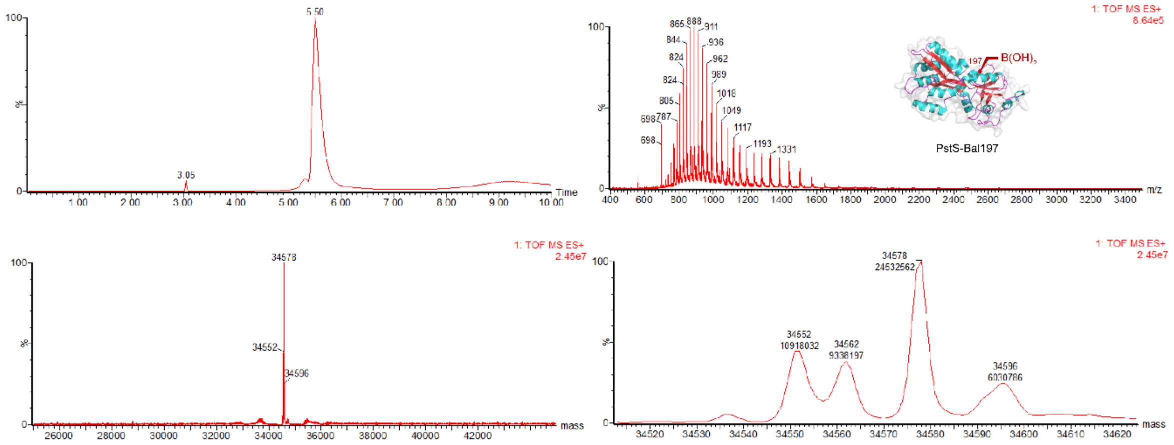

Conversion

PstS-Fpc197	PstS-Ala197	PstS-Bal197		
		Bal	Bal-H_2O	Bal-$2H_2O$
0%	22%	11%	48%	18%

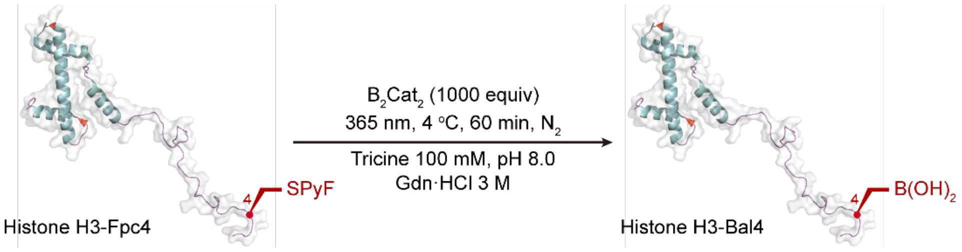

The HistoneH3-Bal4 was prepared according to general procedure 8.

ESI-MS spectrum for the modified histone is shown below.

Calculated mass = 15226 (Bal), 15208 (Bal-H_2O)

Observed mass = 15224 (Bal), 15207 (Bal-H_2O).

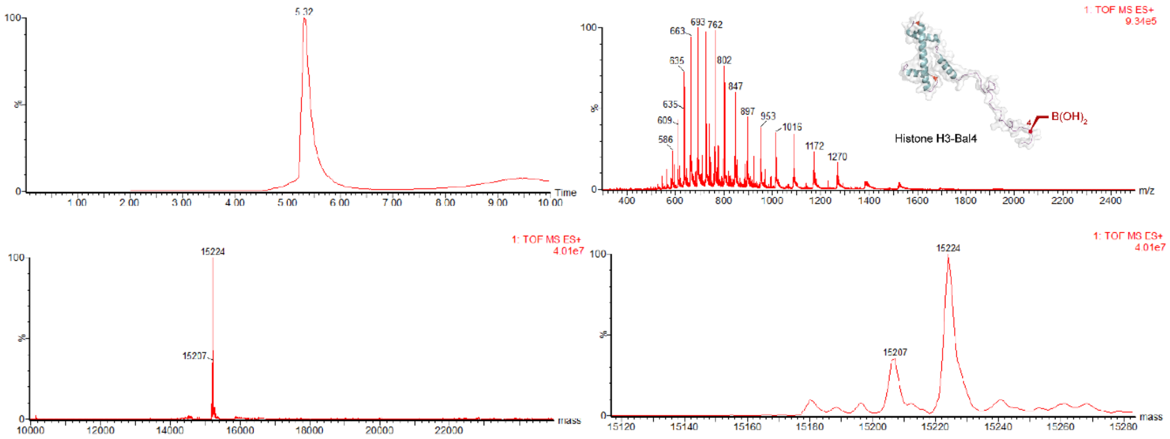

Conversion

Histone H3-Fpc4	Histone H3-Ala4	Histone H3-Bal4		
		Bal	Bal-H_2O	Bal-$2H_2O$
0%	0%	71%	29%	0%

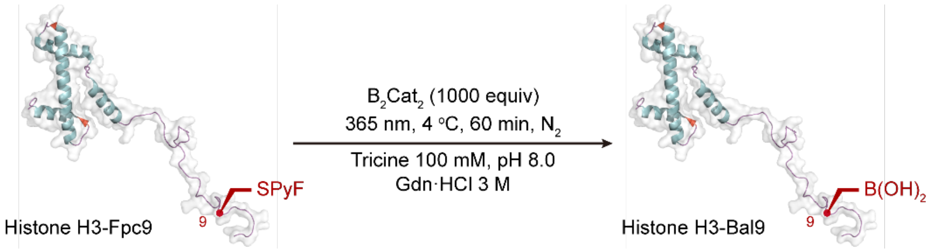

The Histone H3-Bal9 was prepared according to general procedure 8.

ESI-MS spectrum for the modified histone is shown below.

Calculated mass = 15226 (Bal)

Observed mass = 15225 (Bal)

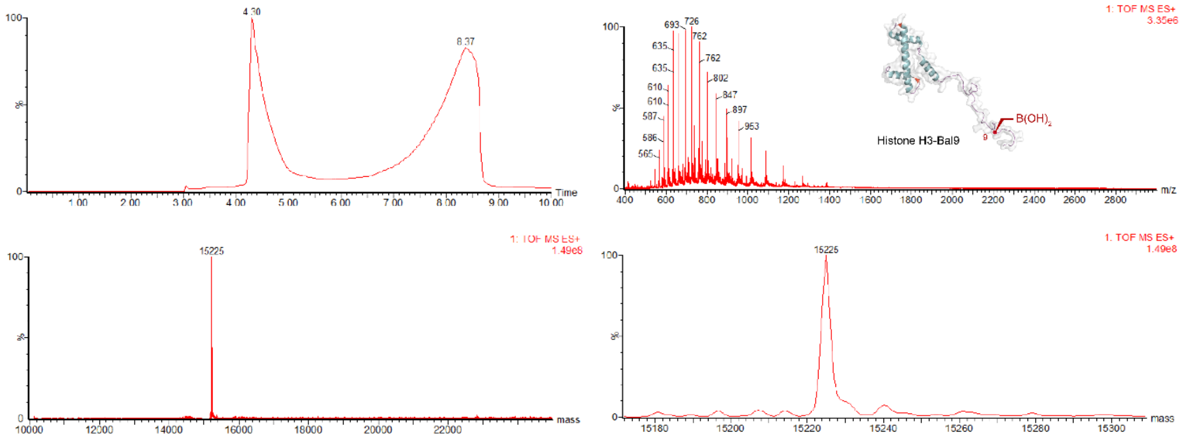

Conversion

Histone H3-Fpc9	Histone H3-Fpc9	Histone H3-Bal9		
		Bal	Bal-H_2O	Bal-$2H_2O$
0%	0%	>98% conversion	0%	0%

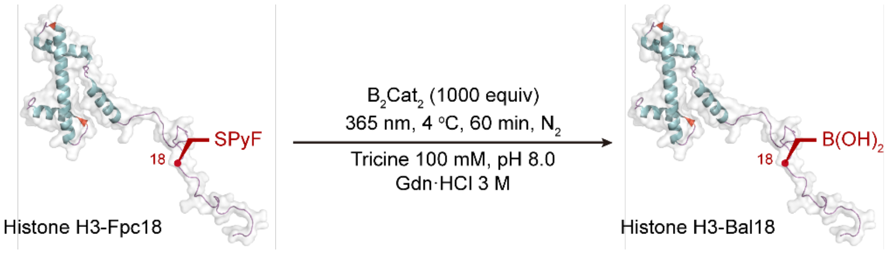

The Histone H3-Bal18 was prepared according to general procedure 8.

ESI-MS spectrum for the modified histone is shown below.

Calculated mass = 15226 (Bal)

Observed mass = 15225 (Bal)

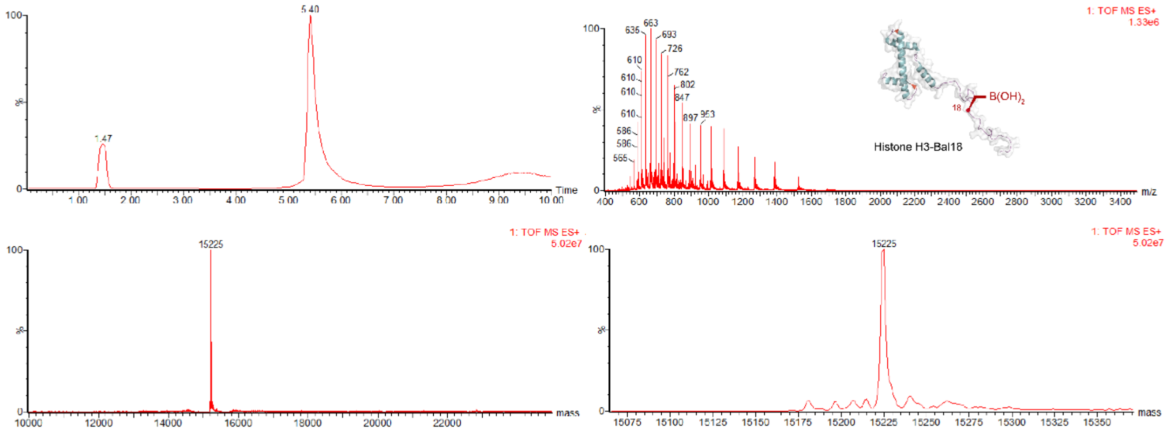

Conversion

Histone H3-Fpc18	Histone H3-Ala18	Histone H3-Bal18		
		Bal	Bal-H_2O	Bal-$2H_2O$
0%	0%	>98% conversion	0%	0%

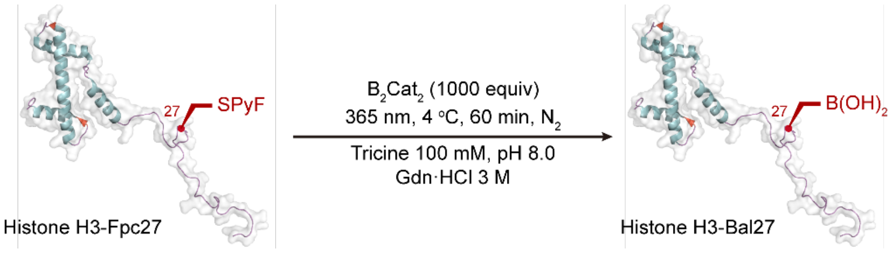

The Histone H3-Bal27 was prepared according to general procedure 8.

ESI-MS spectrum for the modified histone is shown below.

Calculated mass = 15226 (Bal)

Observed mass = 15224 (Bal)

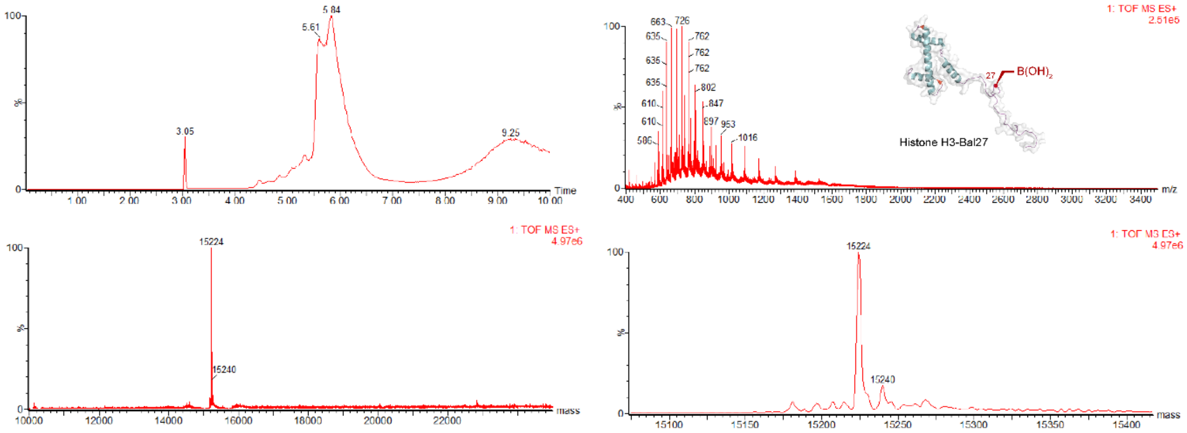

Conversion

Histone H3-Fpc27	Histone H3-Ala27	Histone H3-Bal27		
		Bal	Bal-H_2O	Bal-$2H_2O$
0%	0%	>98% conversion	0%	0%

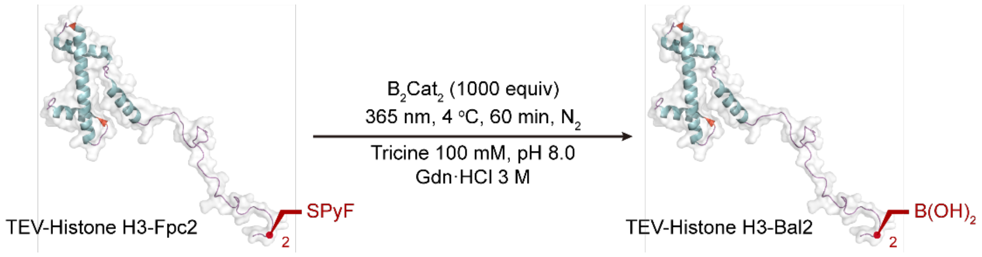

The TEV-Histone H3-Bal2 was prepared according to general procedure 8.

ESI-MS spectrum for the modified histone is shown below.

Calculated mass = 16049 (Bal), 16031 (Bal-H_2O), 16013 (Bal-$2H_2O$)

Observed mass = 16048 (Bal), 16029 (Bal-H_2O), 16012 (Bal-$2H_2O$)

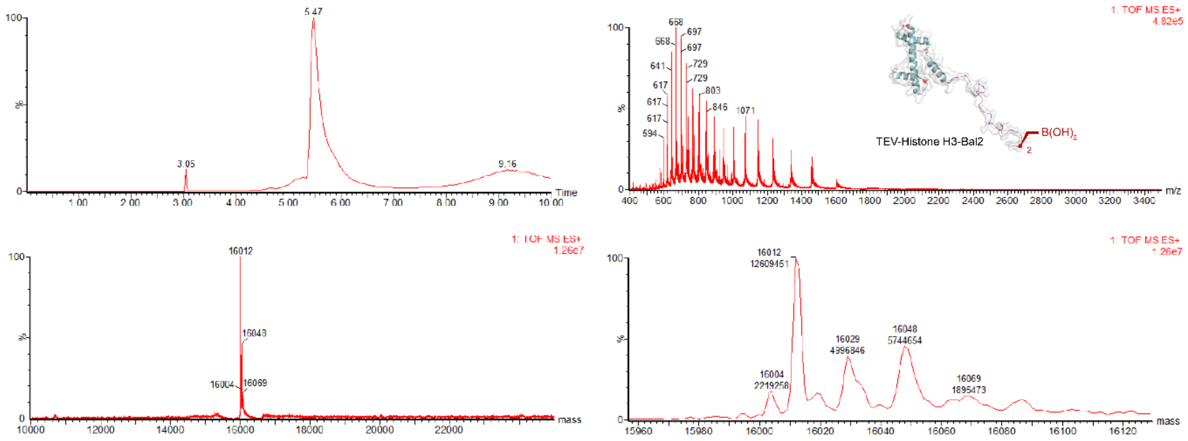

Conversion

TEV-Histone H3-Bal2	TEV-Histone H3-Ala2	TEV-Histone H3-Bal2		
		Bal	Bal-H_2O	Bal-$2H_2O$
0%	0%	23%	22%	55%

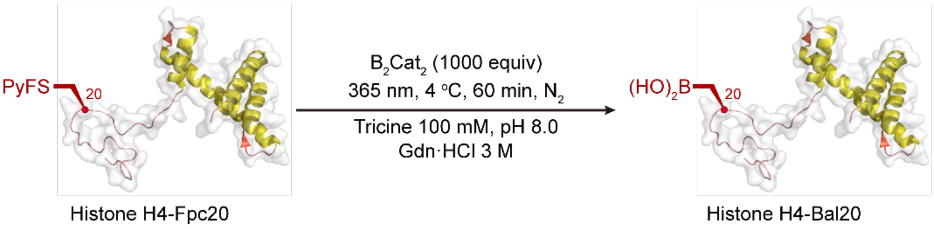

The Histone H4-Bal20 was prepared according to general procedure 8.

ESI-MS spectrum for the modified histone is shown below.

Calculated mass = 11223 (Bal), 11205 (Bal-H_2O)

Observed mass = 11222 (Bal), 11204 (Bal-H_2O)

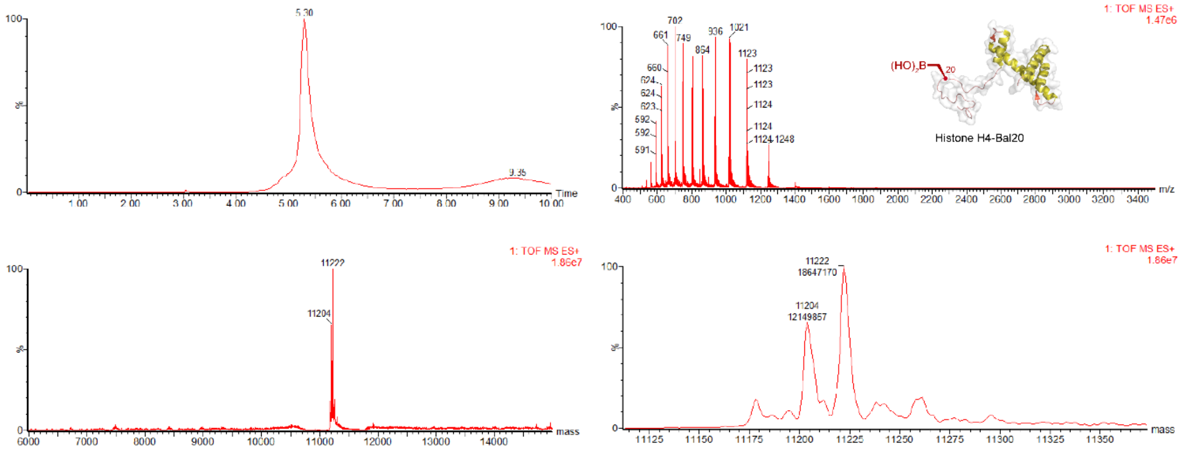

Conversion

Histone H4-Fpc20	Histone H4-Ala20	Histone H4-Bal20		
		Bal	Bal-H_2O	Bal-$2H_2O$
0%	0%	62%	38%	0%

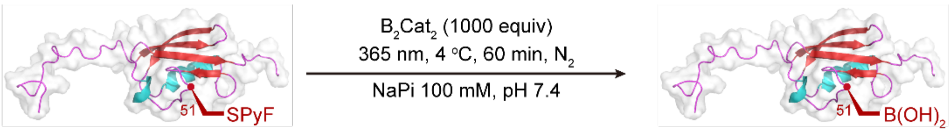

pre-SUMO1-Fpc51 pre-SUMO1-Bal51

The pre-SUMO1-Bal51 was prepared according to general procedure 8.

ESI-MS spectrum for the modified pre-SUMO1 is shown below.

Calculated mass = 12487 (Bal), 12469 (Bal-H_2O)

Observed mass = 12486 (Bal), 12469 (Bal-H_2O)

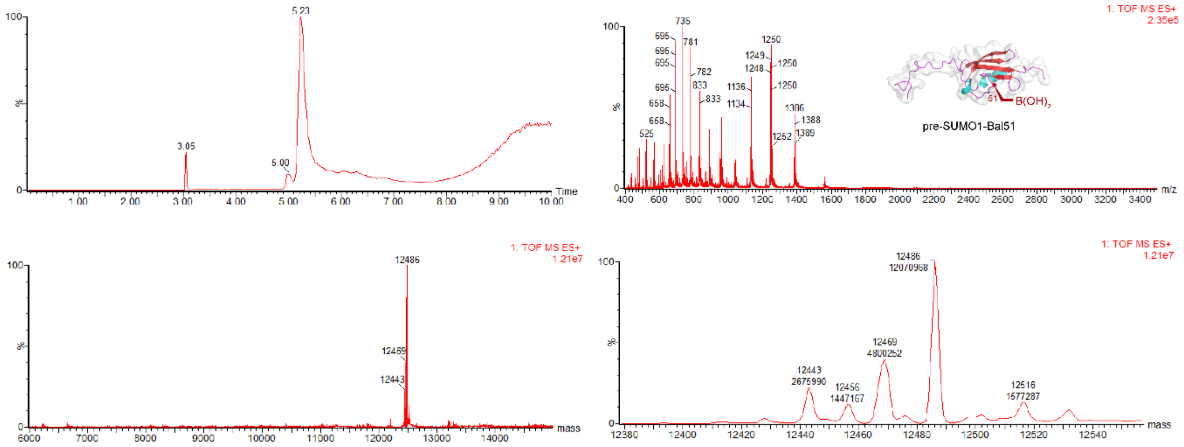

Conversion

pre-SUMO1-Fpc51	pre-SUMO1-A51	pre-SUMO1-Bal51		
		Bal	Bal-H_2O	Bal-$2H_2O$
0%	15%	63%	21%	0%

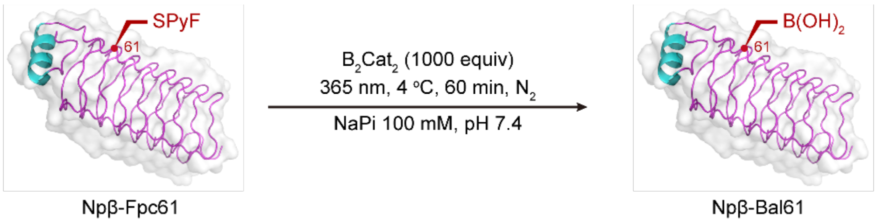

The Npβ-Bal61 was prepared according to general procedure 8.

ESI-MS spectrum for the modified Npβ is shown below.

Calculated mass = 21043 (Bal), 21025 (Bal-H_2O), 21007 (Bal-2H_2O)

Observed mass = 21040 (Bal), 21024 (Bal-H_2O), 21006 (Bal-2H_2O)

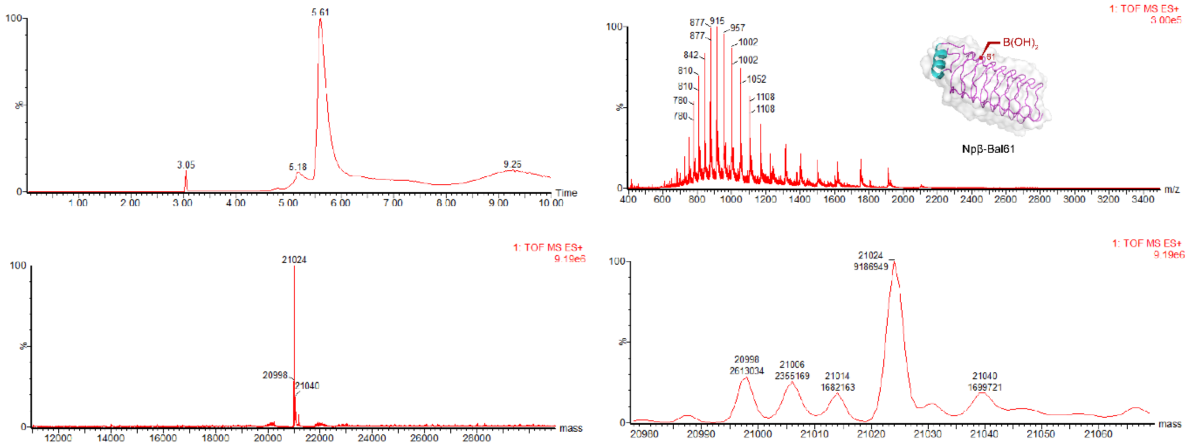

Conversion

Npβ-Fpc61	Npβ-Ala61	Npβ-Bal61		
		Bal	Bal-H_2O	Bal-2H_2O
0%	16%	11%	58%	15%

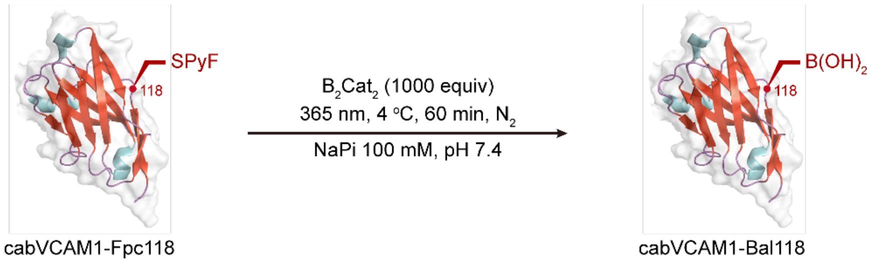

The cAbVCAM1-Bal118 was prepared according to general procedure 8.

ESI-MS spectrum for the modified cAbVCAM1 is shown below.

Calculated mass = 14631 (Bal), 14613 (Bal-H_2O), 14595 (Bal-$2H_2O$)

Observed mass = 14631 (Bal), 14613 (Bal-H_2O), 14595 (Bal-$2H_2O$)

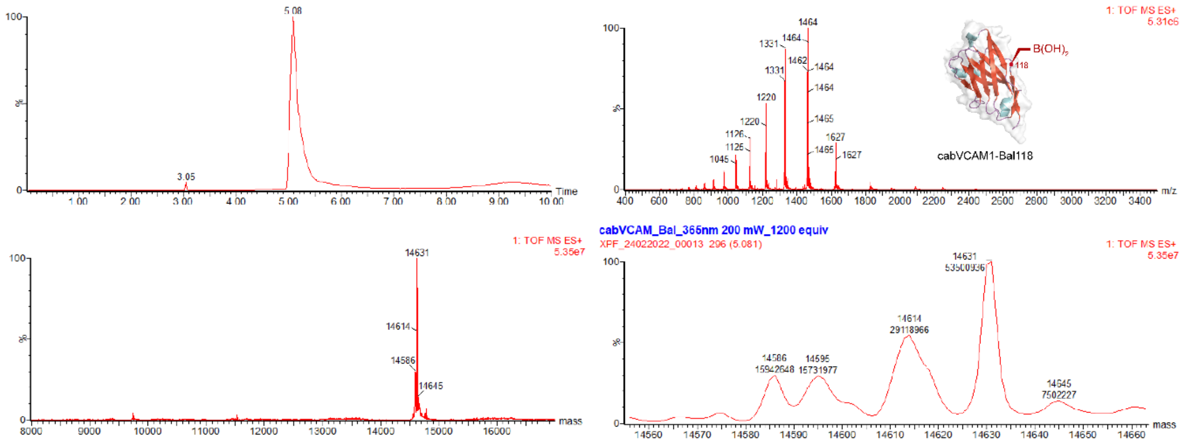

Conversion

cAbVCAM1-Fpc118	cAbVCAM1-Ala118	cAbVCAM1-Bal118		
		Bal	Bal-H_2O	Bal-$2H_2O$
0%	3%	46%	26%	14%

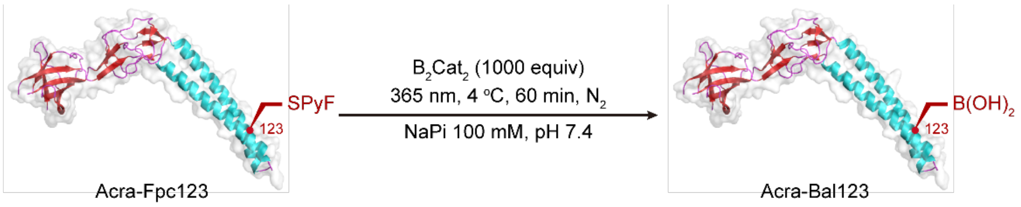

The AcrA-Bal123 was prepared according to general procedure 8.

ESI-MS spectrum for the modified AcrA is shown below.

Calculated mass = 38811 (Bal-H_2O)

Observed mass = 38812 (Bal-H_2O)

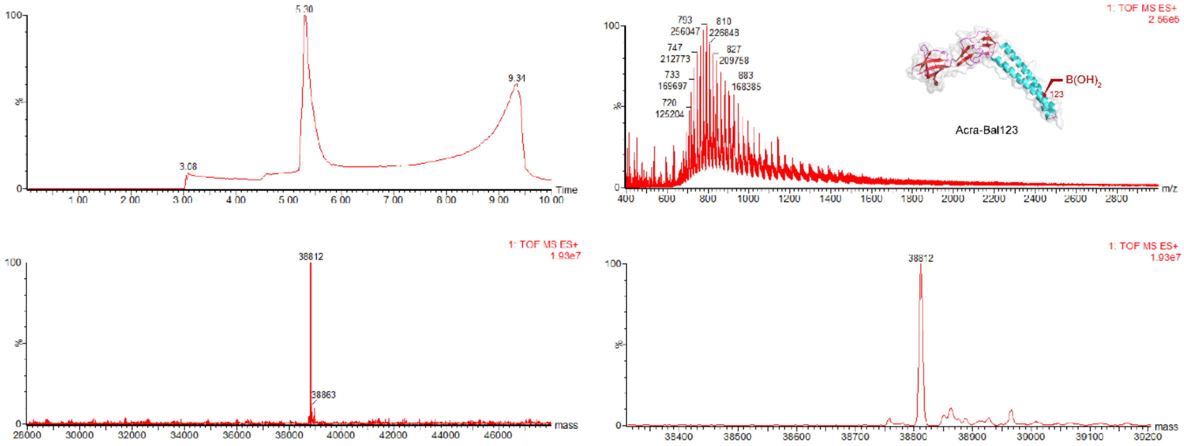

Conversion

AcrA-Fpc123	AcrA-Ala123	AcrA-Bal123		
		Bal	Bal-H_2O	Bal-$2H_2O$
0%	3%	0%	>98% conversion	0%

Characterization of the retention of native L-stereochemistry by protein ^{19}F NMR.

Protein ^{19}F-NMR using shift reagent: Determination of the Histone ^{TEV}H3-L-Bal9. Histone ^{TEV}H3-L-Bal9 was prepared according to general procedure 8 and then it was desalted to binding buffer (40 mM NaPi, 5 M urea, pH 7.0, 10% D_2O) at a final concentration of 97 μM. Then 10 equiv. of chiral shift reagent were added and the sample was vortexed. The sample was transferred to a NMR tube and analyzed on a Bruker AVIII 600 MHz spectrometer equipped with a Prodigy N_2 broadband cryoprobe (3,500 scans, d1 = 2 s).

Protein ^{19}F-NMR using shift reagent of reference epimeric mixture: Determination of the Histone H3-D/L-Bal9. Histone H3-D/L-Bal9 was prepared as reported[15] and then it was desalted to binding buffer (40 mM NaPi, 5 M urea, pH 7.0, 10% D_2O) at a final concentration of 144 μM. Then 10 equiv. of chiral shift reagent were added and the sample was vortexed. The sample was transferred to a NMR tube and analyzed on a Bruker AVIII 600 MHz spectrometer equipped with a Prodigy N_2 broadband cryoprobe (3,500 scans, d1 = 2 s).

Protein ^{19}F-NMR using shift reagent with spiking of Histone H3-D/L-Bal9 in binding buffer (40 mM NaPi, 5 M urea, pH 7.0, 10% D_2O) was mixed with Histone H3-L-Bal9 in binding buffer (40 mM NaPi, 5 M urea, pH 7.0, 10% D_2O), then transferred to a NMR tube and analyzed on a Bruker AVIII 600 MHz spectrometer equipped with a Prodigy N_2 broadband cryoprobe (3,500 scans, d1 = 2 s).

Creation of H3-L-KAc18 and Assessment of Enzymatic Processing as a Substrate using Sirt 2

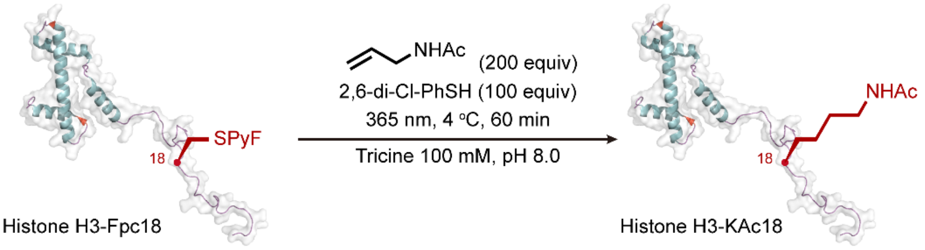

The Histone_H3_18KAc was prepared according to general procedure 5.

ESI-MS spectrum for the modified histone is shown below.

Calculated mass = 15281

Observed mass = 15280

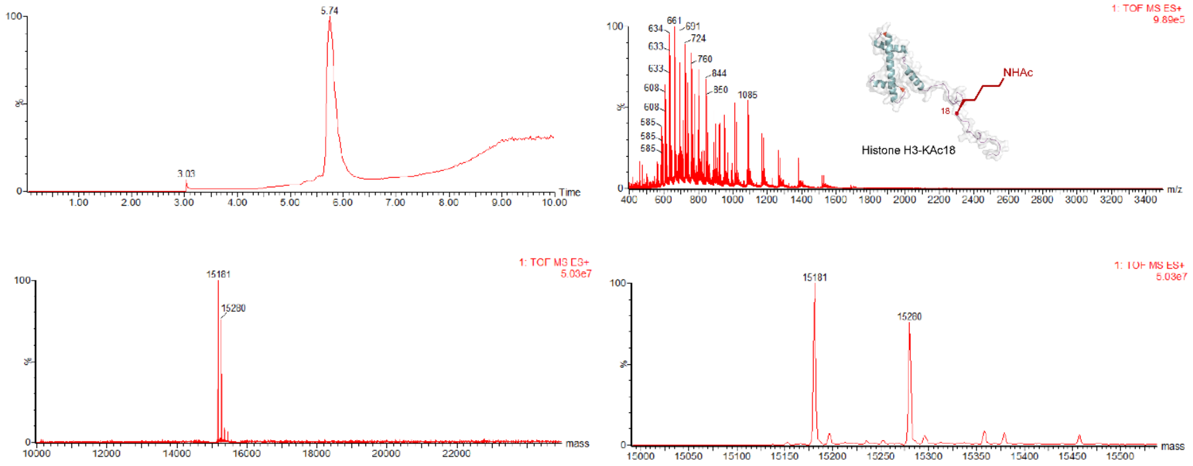

Histone H3-Fpc18	Histone H3-Ala18	Histone H3-KAc18
0%	55%	45%

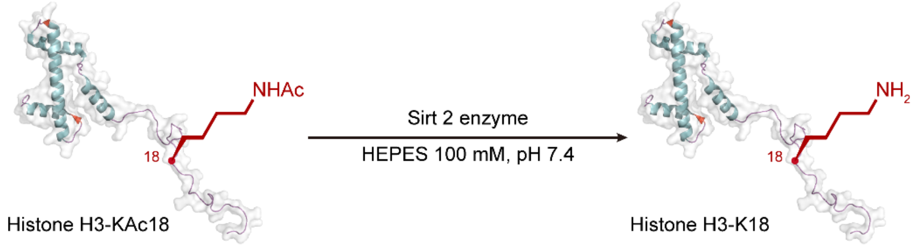

The Histone H3-K18 was prepared according to following procedure.

Histones H3-KAc18 was dialyzed thrice against HEPES buffer (20 mM, pH 7.4), twice for 2 h, once overnight at 4 °C. The solutions were pre-warmed to 37 °C, and Sirt2 (0.5 µg from a stock in the same buffer) and NAD^+ (150 µM final concentration from a 10x stock in buffer) were added to solutions containing either Histone H3-KAc18 (20 µM histone, 50 µL final reaction volume). The reactions were shaken at 37 °C, 600 rpm, with aliquots of the crude reaction mixture taken out at 2 time points (30 min, 15 h), diluted (1:50 in H_2O + 1% formic acid) and immediately analysed via LC-MS.

ESI-MS spectrum for the modified histone is shown below.

Calculated mass = 15238

Observed mass = 15238

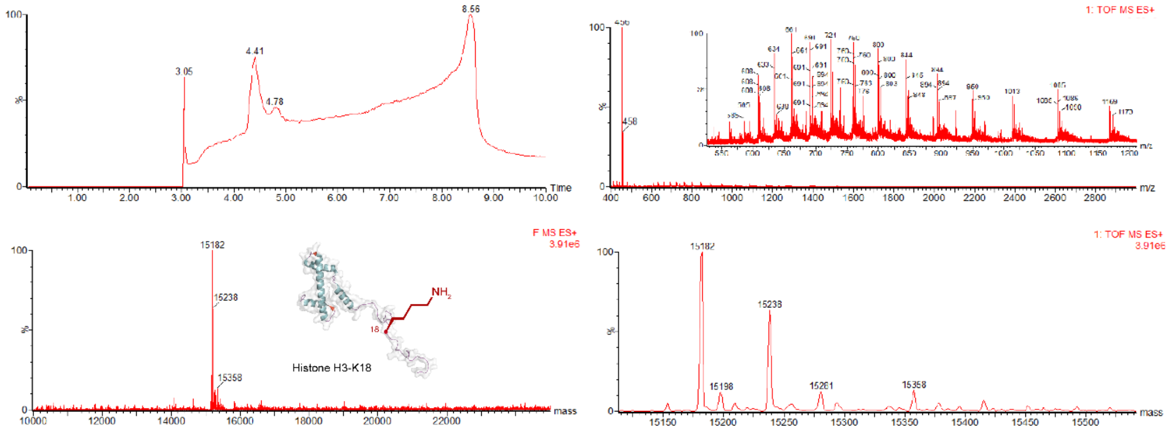

Marfey's Analysis of TEV-HistoneH3-Ser2

TEV-HistoneH3-Ser2 was prepared from TEV-HistoneH3-Fpc2 in the usual way as follows. TEV-HistoneH3-Fpc2 (32 μM, 50 μL) in Tris buffer (20 mM, NaCl 150 mM, pH 8.0) and B_2Cat_2 (50 mM in H_2O, 100 equiv, 32 μL) were mixed and irradiated with 365 nm light at 4 ˚C for 60 min. The reaction mixture was then analysed by LC-MS (calculated mass = 16021, observed mass = 16021, see **Supplementary Figure S17a**). Product TEV-HistoneH3-Ser2 protein was treated with TEV protease and the reaction mixture analysed by LC-MS (calculated mass = 15068, observed mass = 15068 see **Supplementary Figure S17b**).

The resulting N-terminal octapeptide (ASENLYFQ$^{1-8}$, see **Supplementary Figure S17c**) was separated by HPLC. see **Supplementary Figure S17a** An aliquot of the TEV protease digestion mixture (10 μL) was injected onto an Acquity BEH C18 column, 1.7 μm, 2.1 x 50 mm, 45 ˚C, with a 0.8 mL/min, 2.1 min linear gradient from 5% to 40% MeCN/H_2O with 0.1% formic acid. The cleaved peptide was detected by both UV-Vis (diode array) and ESI-MS (1.24 min).

Using the method of Marfey,[16] L-FDAA derivatives of amino acid standards found in the N-terminal peptide were prepared as a standard mixture. 50 mM L- or D- amino acid dissolved in H_2O (50 μL) was treated with 1M $NaHCO_3$ (20 μL) and L- FDAA (1% solution in acetone, 100 μL) at 40 °C for 1 h, after which the reaction was neutralized with 1 M HCl (20 μL), diluted with MeCN (810 μL) and filtered (0.45 μm PTFE) prior to HPLC-DAD-ESIMS analysis. Each L-amino acid component (10 μL) was added to a 1.5 mL Eppendorf and mixed together to make a standard / control mixture (standard / control mixture 1: L-FDAA-L-Ala, L-FDAA-L-Ser, L-FDAA-L-Glu, L-FDAA-L-Asp, L-FDAA-L-Leu, L-FDAA-L-Phe, L-FDAA-L-Tyr; standard / control mixture 2: L-FDAA-L-Ala, L-FDAA-L-Ser, L-FDAA-D-ser, L-FDAA-L-Glu, L-FDAA-L-Asp, L-FDAA-L-Leu, L-FDAA-L-Phe, L-FDAA-L-Tyr).

An aliquot of analyte (5 μL) was injected onto an Acquity BEH C18 column, 1.7 μm, 2.1 x 50 mm, 60 °C, with a 0.8 mL/min, 7.5 min linear gradient from 5% to 95% MeCN/H_2O with 0.1% formic acid. The mixture was assessed by UV (diode array) and ESI-MS (see **Supplementary Figure S17d**). Retention times observed: L-FDAA-L-Ser 3.32 min; L-FDAA-L-Asp 3.54 min; L-FDAA-D-ser 3.59 min; L-FDAA-L-Glu 4.08 min; L-FDAA 4.30; L-FDAA-L-Ala 4.54 min, L-FDAA-L-Tyr 5.56 min; L-FDAA-L-Leu and L-FDAA-L-Phe ~6.73 min. Importantly, L-FDAA-L-Ser (retention time 3.32 min) can be separated from L-

FDAA-D-ser (retention time 3.54); L-FDAA-L-Asp (retention time 3.59 min) is an overlapped shoulder but additional use of EIC allowed clear distinction.

For analysis, a sample of the N-terminal peptide (242 µg, calculated based on 4 mg of starting protein) in 6 M HCl (200 µL) was heated at 100 °C in a sealed vial overnight, after which the hydrolysate was concentrated to dryness at 40 °C under a stream of dry N_2. The hydrolysate was then treated with 1 M $NaHCO_3$ (20 µL) and L-FDAA (1% solution in acetone, 40 µL) at 40 °C for 1 h, after which the reaction was neutralised with 1 M HCl (20 µL), then diluted with 100 µL CH_3CN and centrifuged prior to MS analysis. If necessary, the mixture could be evaporated again, and dissolved in 1 M $NaHCO_3$ (100 µL), washed with ether acetate (× 3, 100 µL), neutralised with 1 M HCl (100 µL), then used for MS analysis. An aliquot of analyte (5 µL) was injected onto an Acquity BEH C18 column, 1.7 µm, 2.1 × 50 mm, 60 °C, with a 0.8 mL/min, 7.5 min linear gradient from 5% to 95% MeCN/H_2O with 0.1% formic acid. The mixture was assessed by UV (diode array) and ESI-MS (see **Supplementary Figure 17**). No L-FDAA-D-Ser could be detected.

DFT Calculations for Model PyF Containing Systems

QM calculations were performed using the basis sets shown below using GAUSSIAN 16. Molecular systems with different electron withdrawing substituents on electron acceptor were used as shown.

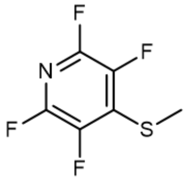

2,3,5,6-tetrafluoro-4-(methylthio)pyridine

P_1

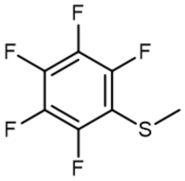

methyl(perfluorophenyl)sulfane

P_2

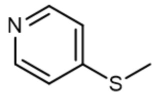

4-(methylthio)pyridine

P_3

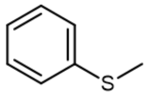

methyl(phenyl)sulfane

P_4

LUMO-SOMO gap (eV) for electron acceptors' radical anions:

	**B3LYP/TZVP**	**B3LYP/631G(d,p)**	**B3LYP/6311G(d,p)**
**P1**	3.374	3.488	3.343
**P2**	3.466	3.671	3.349
**P3**	2.620	2.826	2.731
**P4**	2.244	2.449	2.363

	**B3LYP/TZVP**	**B3LYP/631G(d,p)**	**B3LYP/6311G(d,p)**
**P1**	-2845319.162	-2844573.742	-2845179.181
**P2**	-3063909.714	-3063059.803	-3063739.618
**P3**	-1801974.043	-1801696.223	-1801958.628
**P4**	-1759773.025	-1759518.792	-1759774.610

Optimized energy (kJ/mol) for electron acceptors' radical anions:

S-C(sp3) bond length (Å) comparison for electron acceptors: neutral vs radical anions:

	**B3LYP/TZVP**		**B3LYP/631G(d,p)**		**B3LYP/6311G(d,p)**	
	**Neutral**	**Radical Anion**	**Neutral**	**Radical Anion**	**Neutral**	**Radical Anion**
**P1**	1.83792	1.87942	1.83225	1.89356	1.83315	1.89074
**P2**	1.83938	1.84511	1.83489	1.84455	1.83458	1.84248
**P3**	1.826	1.93578	1.82269	1.92979	1.82122	1.92908
**P4**	1.8388	2.0158	1.83608	1.99276	1.83542	1.99583

S-C(Ar) bond length (Å) comparison for electron acceptors: neutral vs radical anions:

	**B3LYP/TZVP**		**B3LYP/631G(d,p)**		**B3LYP/6311G(d,p)**	
	**Neutral**	**Radical Anion**	**Neutral**	**Radical Anion**	**Neutral**	**Radical Anion**
**P1**	1.77548	1.77987	1.76877	1.74407	1.77121	1.75031
**P2**	1.78676	1.78593	1.78047	1.77401	1.78101	1.77767
**P3**	1.7778	1.74722	1.77506	1.74119	1.77432	1.74197
**P4**	1.80412	1.74297	1.79954	1.73881	1.79989	1.74001

	**B3LYP/TZVP**		**B3LYP/631G(d,p)**		**B3LYP/6311G(d,p)**	
	**HOMO (eV)**	**LUMO (eV)**	**HOMO (eV)**	**LUMO (eV)**	**HOMO (eV)**	**LUMO (eV)**
**P1**	-7.198	-1.984	-6.835	-1.439	-7.121	-1.862
**P2**	-6.886	-1.583	-6.535	-1.117	-6.811	-1.468
**P3**	-6.274	-0.833	-6.229	-0.583	-6.411	-0.858
**P4**	-6.441	-0.959	-6.095	-0.476	-6.298	-0.781

Optimized energy levels for neutral electron acceptors:

Supplementary References

(1) Walling, C.; Rabinowitz, R. THE REACTION OF THIYL RADICALS WITH TRIALKYL PHOSPHITES 1. *Journal of the American Chemical Society* **1957**, *79* (19), 5326-5326. DOI: 10.1021/ja01576a077.

(2) Walling, C.; Basedow, O. H.; Savas, E. S. Some Extensions of the Reaction of Trivalent Phosphorus Derivatives with Alkoxy and Thiyl Radicals; a New Synthesis of Thioesters 1. *Journal of the American Chemical Society* **1960**, *82* (9), 2181-2184. DOI: 10.1021/ja01494a023

(3) Wan, Q.; Danishefsky, S. J. Free-Radical-Based, Specific Desulfurization of Cysteine: A Powerful Advance in the Synthesis of Polypeptides and Glycopolypeptides. *Angewandte Chemie International Edition* **2007**, *46* (48), 9248-9252. DOI: 10.1002/anie.200704195.

(4) Haase, C.; Rohde, H.; Seitz, O. Native Chemical Ligation at Valine. *Angewandte Chemie International Edition* **2008**, *47* (36), 6807-6810. DOI: 10.1002/anie.200801590.

(5) Rohde, H.; Seitz, O. Ligation—Desulfurization: A powerful combination in the synthesis of peptides and glycopeptides. *Peptide Science* **2010**, *94* (4), 551-559. DOI: 10.1002/bip.21442.

(6) Griffiths, R. C.; Smith, F. R.; Long, J. E.; Williams, H. E. L.; Layfield, R.; Mitchell, N. J. Site-Selective Modification of Peptides and Proteins via Interception of Free-Radical-Mediated Dechalcogenation. *Angewandte Chemie International Edition* **2020**, *59* (52), 23659-23667. DOI: 10.1002/anie.202006260.

(7) Griffiths, R. C.; Smith, F. R.; Long, J. E.; Scott, D.; Williams, H. E. L.; Oldham, N. J.; Layfield, R.; Mitchell, N. J. Site-Selective Installation of Nϵ-Modified Sidechains into Peptide and Protein Scaffolds via Visible-Light-Mediated Desulfurative C–C Bond Formation. *Angewandte Chemie International Edition* **2022**, *61* (2), e202110223. DOI: 10.1002/anie.202110223.

(8) Metanis, N.; Keinan, E.; Dawson, P. E. Traceless Ligation of Cysteine Peptides Using Selective Deselenization. *Angewandte Chemie International Edition* **2010**, *49* (39), 7049-7053. DOI: 10.1002/anie.201001900.

(9) Bernardes, G. J. L.; Grayson, E. J.; Thompson, S.; Chalker, J. M.; Errey, J. C.; El Oualid, F.; Claridge, T. D. W.; Davis, B. G. From Disulfide- to Thioether-Linked Glycoproteins. *Angewandte Chemie International Edition* **2008**, *47* (12), 2244-2247. DOI: 10.1002/anie.200704381.

(10) Josephson, B.; Fehl, C.; Isenegger, P. G.; Nadal, S.; Wright, T. H.; Poh, A. W. J.; Bower, B. J.; Giltrap, A. M.; Chen, L.; Batchelor-McAuley, C.; et al. Light-driven post-translational installation of reactive protein side chains. *Nature* **2020**, *585* (7826), 530-537. DOI: 10.1038/s41586-020-2733-7.

(11) Fleming, S. A.; Jensen, A. W. Photocleavage of benzyl-sulfide bonds. *The Journal of Organic Chemistry* **1993**, *58* (25), 7135-7137. DOI: 10.1021/jo00077a041.

(12) Maslak, P.; Theroff, J. Intrinsic Barriers of the Alternative Modes of Mesolytic Fragmentations of C−S Bonds. *Journal of the American Chemical Society* **1996**, *118* (30), 7235-7236. DOI: 10.1021/ja960735x.

(13) Kong, A. T.; Leprevost, F. V.; Avtonomov, A. M.; Mellacheruvu, D.; Nesvizhskii, A. I., MSFragger: ultrafast and comprehensive peptide identification in mass spectrometry-based proteomics. *Nat. Methods* **2017**, 14, 513-520.

(14) Wright, T. H.; Bower, B. J.; Chalker, J. M.; Bernardes, G. J. L.; Wiewiora, R.; Ng, W.-L.; Raj, R.; Faulkner, S.; Vallée, M. R.J.; Phanumartwiwath, A.; Coleman, O. D.; Thézénas, M.-L.; Khan, M.; Galan, S. R. G.; Lercher, L.; Schombs, M. W.; Gerstberger, S.; Palm-Espling, M. E.; Baldwin, A. J.; Kessler, B. M.; Claridge, T. D. W.; Mohammed, S.; Davis, B. G., Posttranslational mutagenesis: a chemical strategy for exploring protein side-chain diversity. *Science* **2016**, *354*, aag1465.

(15) Mollner, T. A.; Isenegger, Josephson, P.G.; B.; Buchanan, C.; Lercher, L.; Oehlrich, D.; Hansen, D.F.; Mohammed, S.; Baldwin, A.J.; Gouverneur, V.; Davis, B.G., Post-translational Insertion of Boron in Proteins to Probe and Modulate Function. *Nat. Chem. Biol.* **2021**, *184*, 2680-2695.

(16) Marfey, P., Determination of D-amino acids. II. Use of a bifunctional reagent, 1,5-difluoro-2,4-dinitrobenzene. *Carlsberg Research Communications*, **1984**, *49*, 591.
