# Supplementary material for: Stereoretentive Post-Translational Protein Editing
Source: ACS Cent Sci. 2023 Feb 24;9(3):405–16. doi: 10.1021/acscentsci.2c00991 (PMC10037454; doi:10.1021/acscentsci.2c00991)
Supplement: Supplementary file 2 — oc2c00991_si_002.pdf [file oc2c00991_si_002.pdf]

oc-2022-00991v.R1

Name: Peer Review Information for "Stereoretentive Post-Translational Protein Editing"

First Round of Reviewer Comments

Reviewer: 1

Comments to the Author

The manuscript by Davis and coworkers establishes a method for the generation and functionalization of "on protein" radicals. Specifically, carefully designed fluoroaryl-Cys precursors are prepared using a Cys-selective arylation, and subsequent light-mediated C–S bond cleavage generates alanyl radicals poised for further modification. The protocol is demonstrated on a range of proteins and various photochemical initiation conditions are examined to enable programmable conversion of Cys to Ala as well as the establishment of C–B, C–O, C–Se, and C–C bonds. Importantly, careful analysis of the stereochemistry of the modified amino acid residues was also undertaken on three separate examples using different analytical tools/assays, providing convincing evidence that L-configuration is retained during the reaction sequence. Consequently, the present strategy overcomes a key limitation of existing Dha-based methods for protein functionalization and is an exciting development. In my opinion the work will be of broad interest and therefore warrants publication in ACS Central Science.

I would suggest the following revisions prior to publication:

- A point is made early on in the rationale for the work (e.g. bottom of p. 6) that TCEP-based desulfurative methods lead to the disruption of disulfides and hence have limited application on larger/folded protein examples. In the present work, the authors do not appear to have tested their light-mediated reductive approach on protein examples containing disulfides. The authors should examine the compatibility of the method in the presence of disulfides (if not on a protein, then on a polypeptide example).

- On p. 6: "In peptidic systems…limited to a peptide scaffold". The phrase "limited to a peptide scaffold" seems redundant in this sentence. I would suggest removing this or rephrasing.

- In the captions to Figures 2 and 3, it should be more clearly specified whether reported values represent % yield or % conversion.

- In reference to the sentence on p. 19, lines 43-47: "Interestingly…leads to concomitant dimerization", it is unclear to me how the dimerization process occurs. Can the authors comment in more detail?

- The final sentence on p. 23 ("This arises from cleavage…") is lengthy and difficult to follow. I would suggest rephrasing for clarity.

Reviewer: 2

Comments to the Author

The manuscript by Fu et al. focuses on a multistep route to chemical edit cysteine residues within proteins to other amino acid sidechains. The cysteines are first arylated with a 100-fold excess of perfluoropyridine. Next, using photoredox, the C-S bond is homolytically cleaved generating effectively an alanine radical, with the radical at the beta position. Given an excess of a suitable radical acceptor (one example used a 1000-fold excess of B2cat2 as both photoredox agent and the radical acceptor), one can generate various adducts to the alanyl radical. The process is demonstrated on multiple different proteins, and C-B, C-C, C-Se, and C-O bonds can be formed with varying success. Perhaps the most exciting aspect of the work is that the chemistry appears to occur without any epimerization at the alpha position. As such, this technique is much more useful than previous routes from the Davis group that relied on the formation of dehydroalaine, leading to epimerized mixtures. The major limitation of the chemistry is that it must be carried out in a glovebox (less than 10 ppm O2), or the alanyl radicals will just go to serine. The reactions also have to be driven by UV light and with large excesses of the arylation reagent and radical capture agents. These limitations will probably limit the use of this technology by bench biochemists, especially since many of the unnatural amino acids investigated can be accessed using sense or nonsense codon suppression. All of this being said, this is still an interesting finding, especially the ability to retain stereochemistry, and I would support publication if the other reviewers are more enthusiastic about the applications. I have several concerns/suggestions listed below in the order in which they appear in the manuscript.

Page 3: abstract, the word SOMOphile is not defined, and more generally the abstract is overly jargony, relying on the readers to know a lot about the authors' previous work on dehydroalanine. Would suggest focusing on the novelty of what was found in the current work rather than a comparison to previous work.

Page 4: a little nitpicky, but it is stated that all amino acids form Cbeta-X bonds, but this is not true for Gly

Page 4: the passage starting with "utilizing Cbeta to catenate…" is jargony and confusing, in part because it's not clear what a "protein synthon" is

Page 4: define d.r.s

Page 5: what is meant by a "ready" stereoretentive method?

Page 6: define "Fpc" at first use

Page 7-8: when discussing the strategy, (2nd half of pg 7 and most of pg 8) there are no figures or SI figures to help explain this very technical section.

Page 9: when mentioning the DFT calculations, the paper should direct the reader to a specific SI figure, not just the entire SI

Page 10: when describing the reaction conditions in the text, the time of the reaction should be including as well as the atmosphere (ie did O2 have to be excluded for the arylation step)

Page 14: when discussing the oxidative damage to protein when using the iridium compound, there is nothing pointing to either the SI or the literature. Since this type of compound is being used more frequently in biocatalysis applications, this information is crucial to add to the paper.

Page 15: The abbreviation Bal appears on this page but hasn't been defined (it is defined later, but should be defined at the first usage)

Page 16: I believe this is the first point in the text that actually discloses that the reactions are being done in a glovebox. It is my opinion that the authors should be more upfront about this requirement.

Page 22: Figure 4a, there is really no reason to show the protein structure over and over again. Suggest converting this figure into a more conventional substrate scope table that shows explicitly the radical acceptor, since this is not immediately obvious from the structures of the products.

Page 23: in reading this caption for figure 4b, it was not immediately obvious where the "polymerization" was. Suggest adding the word polymerization to Figure 4b or alternatively mentioning the specific phospho radical acceptor in the caption to make a better connection between the figure and the caption.

Page 26: While the three methods used are pretty convincing that the L-enantiomer is retained, I wonder why the standard methodology of Marfey's analysis was not carried out instead or in addition to these methods.

Author's Response to Peer Review Comments:

Prof. B. G. Davis
Telephone: +44(0)1235 395008 or **+44(0)1865 275652**
Electronic-mail: Ben.Davis@rfi.ac.uk or **Ben.Davis@chem.ox.ac.uk**

Tuesday, January 3, 2023

Prof. Editor
Senior Editor
ACS Central Science

Re: oc-2022-00991v

Editor,

Thanks indeed again for the invaluable feedback from you and the referees in your email of December 13.

As requested, we have used the intervening 3 weeks to address the points raised. We are pleased to say that this has prompted productive experiments and we now attach a revised, highlighted manuscript and associated files.

In particular, new experiments have allowed us to, as requested:
• demonstrate the efficacy of the reaction in protein scaffolds containing disulfides, specifically in the application to a single-domain antibody system. Moreover, as well as showing the benign nature of the protein edits that we describe here, we were also able to demonstrate the disruptive effect of TCEP in such systems;
• test the efficacy of the classical Marfey's reagent system (often used for peptides and natural products) in the context of protein residue configuration analysis. This further supports the stereo-retentive nature of our edits.

We also address all of the valuable issues raised, point-by-point in the referees' and the editorial / formatting comments listed below.

Referee 1

The manuscript by Davis and coworkers establishes a method for the generation and functionalization of "on protein" radicals. Specifically, carefully designed fluoroaryl-Cys precursors are prepared using a Cys-selective arylation, and subsequent light-mediated C–S bond cleavage generates alanyl radicals poised for further modification. The protocol is demonstrated on a range of proteins and various photochemical initiation conditions are examined to enable programmable conversion of Cys to Ala as well as the establishment of C–B, C–O, C–Se, and C–C bonds. Importantly, careful analysis of the stereochemistry of the modified amino acid residues was

also undertaken on three separate examples using different analytical tools/assays, providing convincing evidence that L-configuration is retained during the reaction sequence. Consequently, the present strategy overcomes a key limitation of existing Dha-based methods for protein functionalization and is an exciting development. In my opinion the work will be of broad interest and therefore warrants publication in ACS Central Science.

• Thank you.

I would suggest the following revisions prior to publication:
- A point is made early on in the rationale for the work (e.g. bottom of p. 6) that TCEP-based desulfurative methods lead to the disruption of disulfides and hence have limited application on larger/folded protein examples. In the present work, the authors do not appear to have tested their light-mediated reductive approach on protein examples containing disulfides. The authors should examine the compatibility of the method in the presence of disulfides (if not on a protein, then on a polypeptide example).

• As requested, the revised manuscript now contains the example of tested editing and free thiol analysis in a disulfide-containing single-domain antibody (nanobody), specifically cabVCAM. Full retention of the disulfide is observed (Supplementary Figure S16).
• Moreover, prompted by the referee's questions we tested the comparable effects of TCEP in the same scaffold. TCEP is fully disruptive as anticipated.
• Thank you for these suggestions.

- On p. 6: "In peptidic systems...limited to a peptide scaffold". The phrase "limited to a peptide scaffold" seems redundant in this sentence. I would suggest removing this or rephrasing.

• Thank you – now corrected.

- In the captions to Figures 2 and 3, it should be more clearly specified whether reported values represent % yield or % conversion.

• Thank you – now added.

- In reference to the sentence on p. 19, lines 43-47: "Interestingly...leads to concomitant dimerization", it is unclear to me how the dimerization process occurs. Can the authors comment in more detail?

• Thank you for this question – our phrasing was indeed unclear. We presume that this occurs through trapping of the radical intermediate by a second equivalent of allylacetamide leading to oligomerization and have now revised the text to add this detail.

- The final sentence on p. 23 ("This arises from cleavage...") is lengthy and difficult to follow. I would suggest rephrasing for clarity.

• Thank you for spotting this – this was indeed clumsy and has now been rewritten.

<u>Reviewer 2</u>

The manuscript by Fu et al. focuses on a multistep route to chemical edit cysteine residues within proteins to other amino acid sidechains. The cysteines are first arylated with a 100-fold excess of perfluoropyridine. Next, using photoredox, the C-S bond is homolytically cleaved generating effectively an alanine radical, with the radical at the beta position. Given an excess of a suitable radical acceptor (one example used a 1000-fold excess of B2cat2 as both photoredox agent and the radical acceptor), one can generate various adducts to the alanyl radical. The process is demonstrated on multiple different proteins, and C-B, C-C, C-Se, and C-O bonds can be formed with varying success. Perhaps the most exciting aspect of the work is that the chemistry appears to occur without any epimerization at the alpha position. As such, this technique is much more useful than previous routes from the Davis group that relied on the formation of dehydroalaine, leading to epimerized mixtures. The major limitation of the chemistry is that it must be carried out in a glovebox (less than 10 ppm O2), or the alanyl radicals will just go to serine. The reactions also have to be driven by UV light and with large excesses of the arylation reagent and radical capture agents.

• Thank you.

These limitations will probably limit the use of this technology by bench biochemists, especially since many of the unnatural amino acids investigated can be accessed using sense or nonsense codon suppression.

• Although surprising, this is in fact not the case. Of the unnatural amino acid examples we demonstrate here, only three [SecPh and KAc (via nonsense systems) and Hag (via sense)] are known.
• We believe that these is ample room for complementary methods and in fact in this case the expansion of the scope of uAAs that can now be placed into proteins through post-translational editing moves beyond co-translational methods.

All of this being said, this is still an interesting finding, especially the ability to retain stereochemistry, and I would support publication if the other reviewers are more enthusiastic about the applications.

• Thank you.

I have several concerns/suggestions listed below in the order in which they appear in the manuscript.
Page 3: abstract, the word SOMOphile is not defined, and more generally the abstract is overly jargony, relying on the readers to know a lot about the authors' previous work on dehydroalanine. Would suggest focusing on the novelty of what was found in the current work rather than a comparison to previous work.

• Thank you for this advice – we have tried to adjust / reword the abstract appropriately, but, as both referees point out, prior methods for post-translational editing represent the appropriate the background.
• We hope we have now been able to strike a balance between the strategic basis for this new work without restating too much past work, as requested.

Page 4: a little nitpicky, but it is stated that all amino acids form Cbeta-X bonds, but this is not true for Gly

• Fair point – not at all nitpicky – thank you.
• We had hoped to be accurate in saying "which is present in all amino acid side chains" – is that sufficient in that Gly has no side chain? Happy to reword if not but we hope this covers it accurately.

Page 4: the passage starting with "utilizing Cbeta to catenate…" is jargony and confusing, in part because it's not clear what a "protein synthon" is

• Thank you for this advice – we have now removed the word catenate and tried to rephrase things in a less dense manner.
• With the regard to 'synthon', we had hoped that the extension of quite well-understood retrosynthetic analyses to protein chemistry might offer a useful perspective for the uninitiated. We had hoped by starting the sentence "*From a retrosynthetic viewpoint,ref 12* …" we could point the reader to appropriate citations where this idea is further developed. We would like to retain this idea if we may.

Page 4: define d.r.s

• Thank you – now done.

Page 5: what is meant by a "ready" stereoretentive method?

• Thank you – we have now altered this colloquialism !

Page 6: define "Fpc" at first use

• Thank you – now done.

Page 7-8: when discussing the strategy, (2nd half of pg 7 and most of pg 8) there are no figures or SI figures to help explain this very technical section.

• We have now added a figure (Supplementary Figure S18) to help frame this section. Thank you for this suggestion.

Page 9: when mentioning the DFT calculations, the paper should direct the reader to a specific SI figure, not just the entire SI

• Thank you - now added.

Page 10: when describing the reaction conditions in the text, the time of the reaction should be including as well as the atmosphere (ie did O2 have to be excluded for the arylation step)

• Thank you – now added.
• The typical reaction time for arylation was just 30 min and could be conducted under ambient conditions without need for oxygen exclusion.

Page 14: when discussing the oxidative damage to protein when using the iridium compound, there is nothing pointing to either the SI or the literature. Since this type of compound is being used more frequently in biocatalysis applications, this information is crucial to add to the paper.

• Thank you – a citation to our previous (quite extensive) analyses [Josephson 2020] of the limits of these types of photocatalysts has now been added.

Page 15: The abbreviation Bal appears on this page but hasn't been defined (it is defined later, but should be defined at the first usage)

• Thank you – now added.

Page 16: I believe this is the first point in the text that actually discloses that the reactions are being done in a glovebox. It is my opinion that the authors should be more upfront about this requirement.

• Thank you – now added.
• Please note that the use of a glovebox primarily allows equilibration of buffer oxygen levels in a convenient way and there is not a stringent requirement for rigorous oxygen exclusion.

Page 22: Figure 4a, there is really no reason to show the protein structure over and over again. Suggest converting this figure into a more conventional substrate scope table that shows explicitly the radical acceptor, since this is not immediately obvious from the structures of the products.

• We do understand the point made but with permission, we would like to retain this format as it remains consistent with the other papers in this format and places an emphasis, we believe appropriately, on the nature of the edited protein product.
• This is particularly pertinent as the identities of the products (i.e. oligomer states) are crucially different from acceptor to acceptor, somewhat different from typical substrate scope tables in small molecule studies.

• To avoid any doubt, the acceptors have been described in detail in the Supplementary Methods with individual schemes and structures for each and we have now added a note to that effect to the manuscript, which we hope will help.
• We did try to draft a variant that combined both but, as might be understood, this lost the intended immediacy of such a 'summary table' format.

Page 23: in reading this caption for figure 4b, it was not immediately obvious where the "polymerization" was. Suggest adding the word polymerization to Figure 4b or alternatively mentioning the specific phospho radical acceptor in the caption to make a better connection between the figure and the caption.

• Thank you for this suggestion – yes – the caption is unhelpfully sparse and we have now added more detail and rewritten.

Page 26: While the three methods used are pretty convincing that the L-enantiomer is retained, I wonder why the standard methodology of Marfey's analysis was not carried out instead or in addition to these methods.

• Thank you for the support of the various analytical methods that we had used to test retention of L-configuration. The referee's question is an excellent one and the use of these methods was driven by the higher sensitivity that they typically afford over other methods (such as chromophoric Marfey/Sanger-type methods), important with the often lower concentrations and amounts of material.
• That said the use of Marfey's is certainly an interesting (and challenging !) suggestion that we decided to tested here. Although to our knowledge essentially limited to peptidic and natural products systems, there is no reason in principle, as the referee points out, that this type of analysis might not be applied to proteins, should sufficient abundance and sensitivity prove possible.
• We chose to test this in a system where we could gauge utility on an intact edited protein either via direct application or through sequential peptide cleavage and then Marfey's procedure.
• In short it is possible to access analyses in this way, demonstrating here again that we observe only an L-configuration in the chosen H3 system, but it does require a relatively large amount of material [of the order of several mg of protein] to gain sufficient sensitivity. Full details are given in Supplementary Figure S17 and the additional methods for these experiments.
• We thank the referee for this suggestion. The data suggest that in certain cases Marfey's analysis might prove to be another complementary method when samples are sufficiently abundant.

Editorial Revision Checklist

SI PARAGRAPH: If the manuscript is accompanied by any supporting information for publication, a brief description of the supplementary material is required in the manuscript. The appropriate format is: Supporting Information. Brief statement in non-sentence format listing the contents of the material supplied as Supporting Information.

• Thank you – now added.

SYNOPSIS MISSING: The synopsis should be no more than 200 characters (including spaces) and should reasonably correlate with the TOC graphic. The synopsis is intended to explain the importance of the article to a broader readership across the sciences. Please place your synopsis in the manuscript file after the TOC graphic.

• Thank you – now added.

SI HEADER: The supporting information should be formatted with a cover sheet listing authors, author affiliations, corresponding author email, manuscript title, and the number of pages, figures, and tables. The Author affiliations must match the MS.

• Thank you – now added.

SI LABELS: Tables, Figures and Equations should be numbered Table S1-Sn, Figure S1-Sn and Equation S1-Sn.

• Thank you – now added.

SI PG#S: The supporting information pages must be numbered consecutively, starting with page S1.

• Thank you – now added.

We look forward to hearing from you.

Yours sincerely,

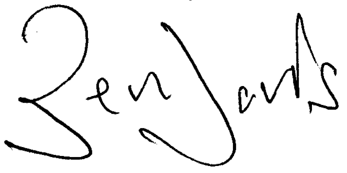

Ben Davis,
on behalf of all authors.

oc-2022-00991v.R2

Name: Peer Review Information for "Stereoretentive Post-Translational Protein Editing"

Second Round of Reviewer Comments

Reviewer: 2

Comments to the Author

The authors have addressed most of my concerns. Two lingering issues from the previous round are

1) Are there other ways of removing oxygen from the solvent besides carrying out the chemistry in a glovebox? Methods like freeze/pump/thaw will likely disrupt proteins. Did the authors carry out any experiments in open atmosphere after sparging their solutions? Is that sufficient to remove the oxygen? Again, my goal here is be able to have bench biochemists have access to this technique, and most do not have easy glovebox access!

2) I still think Figure 4 with the repetitive use of the protein structure (which is too small to see the details) could be condensed or used to show the radical donors. I appreciate that the authors have included the radical donors in the SI, though these schemes are unnumbered and require to reader to search through the 90 page SI to find the information.

Finally, the addition of Figure S18 is appreciated, but it could be improved with a more substantial caption.

Author's Response to Peer Review Comments:

Prof. B. G. Davis
Telephone: +44(0)1235 395008 or **+44(0)1865 275652**
Electronic-mail: Ben.Davis@rfi.ac.uk or **Ben.Davis@chem.ox.ac.uk**

Saturday, February 4, 2023

Prof. Editor
Senior Editor
ACS Central Science

Re: oc-2022-00991v.R1

Editor,

Thanks for your email of January 30th and the great news of the provisional acceptance.

As requested, we have made the requesting formatting changes.

We have also made the following changes in direct response to Reviewer 2's very useful additional comments:
• an additional sentence that describes how removal of oxygen may be considered;
• graphical edits to clarify figure 4;
• a more substantial caption for figure S 18.

Yours sincerely,

Ben Davis,
on behalf of all authors.
